# A novel vaccine based on SARS-CoV-2 CD4^+^ and CD8^+^ T cell conserved epitopes from variants Alpha to Omicron

**DOI:** 10.1038/s41598-022-21207-2

**Published:** 2022-10-06

**Authors:** Iam Palatnik-de-Sousa, Zachary S. Wallace, Stephany Christiny Cavalcante, Maria Paula Fonseca Ribeiro, João Antônio Barbosa Martins Silva, Rafael Ciro Cavalcante, Richard H. Scheuermann, Clarisa Beatriz Palatnik-de-Sousa

**Affiliations:** 1 Department of Electrical Engeneering, Pontifical Catholic University of Rio de Janeiro, Rio de Janeiro, Brazil; 2 Department of Informatics, J. Craig Venter Institute, La Jolla, CA USA; 3 Department of Computer Science and Engineering, University of California, San Diego, CA USA; 4 Department of General Microbiology, Institute of Microbiology Paulo de Góes, Federal University of Rio de Janeiro, Rio de Janeiro, Brazil; 5 Department of Pharmacy, Campus Professor Antônio Garcia Filho, Federal University of Sergipe, Lagarto, Sergipe Brazil; 6 Department of Pathology, University of California, San Diego, CA USA; 7 Division of Vaccine Discovery, La Jolla Institute for Immunology, La Jolla, CA USA; 8 Global Virus Network, Baltimore, MD USA; 9 Institute for Immunological Investigation (III), INCT, National Council for Scientific and Technological Development (CNPq), São Paulo, Brazil

**Keywords:** Vaccines, Protein design

## Abstract

COVID-19 caused, as of September, 1rst, 2022, 599,825,400 confirmed cases, including 6,469,458 deaths. Currently used vaccines reduced severity and mortality but not virus transmission or reinfection by different strains. They are based on the Spike protein of the Wuhan reference virus, which although highly antigenic suffered many mutations in SARS-CoV-2 variants, escaping vaccine-generated immune responses. Multiepitope vaccines based on 100% conserved epitopes of multiple proteins of all SARS-CoV-2 variants, rather than a single highly mutating antigen, could offer more long-lasting protection. In this study, a multiepitope multivariant vaccine was designed using immunoinformatics and in silico approaches. It is composed of highly promiscuous and strong HLA binding CD4^+^ and CD8^+^ T cell epitopes of the S, M, N, E, ORF1ab, ORF 6 and ORF8 proteins. Based on the analysis of one genome per WHO clade, the epitopes were 100% conserved among the Wuhan-Hu1, Alpha, Beta, Gamma, Delta, Omicron, Mµ, Zeta, Lambda and R1 variants. An extended epitope-conservancy analysis performed using GISAID metadata of 3,630,666 SARS-CoV-2 genomes of these variants and the additional genomes of the Epsilon, Lota, Theta, Eta, Kappa and GH490 R clades, confirmed the high conservancy of the epitopes. All but one of the CD4 peptides showed a level of conservation greater than 97% among all genomes. All but one of the CD8 epitopes showed a level of conservation greater than 96% among all genomes, with the vast majority greater than 99%. A multiepitope and multivariant recombinant vaccine was designed and it was stable, mildly hydrophobic and non-toxic. The vaccine has good molecular docking with TLR4 and promoted, without adjuvant, strong B and Th1 memory immune responses and secretion of high levels of IL-2, IFN-γ, lower levels of IL-12, TGF-β and IL-10, and no IL-6. Experimental in vivo studies should validate the vaccine’s further use as preventive tool with cross-protective properties.

## Introduction

COVID-19 is a severe acute respiratory syndrome caused by the SARS-CoV-2 coronavirus, which arose in December 2019 and it is affecting all the continents ever since. As of September 1rst, 2022, there have been 599,825,400 confirmed cases of COVID-19, including 6,469,458 deaths^1^. The Wuhan Hu1 is the ancestral reference virus, but variants Gamma, Delta, Beta and Omicron emerged, respectively, in Brazil, India and South Africa, which are countries with dense populations, that had at that time a low anti-COVID-19 vaccination coverage. In Brazil, for instance, the Gamma variant was first described on January 21th, 2021, just before the start of the vaccination campaign^2^.

According to their increased transmissibility, increased hospitalizations and deaths, reduced neutralization by antibodies generated by previous infection or vaccination, reduced response to treatment or diagnostic failures some of the mutants are considered Variants of Concern (VOC): Alpha (B.1.1.7), Beta (B.1.351), Gamma or P1 (B.1.1.28.1), Delta (lineage B.1.617.2) and Omicron (B.1.1.529), etc.^3^. In contrast, mutants that show changes to receptor binding, but not increased hospitalizations and deaths, are Variants of Interest (VOI): Mµ (B.1.621), Zeta or P.2 (B.1.1.28.2)^3^, Lambda^4^ and R1 (B.1.427/B.1.429) and others^5^. While the ancestral Wuhan Hu1 was worldwide predominant until September 2020, new variants emerged since December 2020 at a much higher rate, consistent with the accumulation of two mutations per month, and to a strong selective pressure on the immunologically important SARS-CoV-2 genes^6^.

Despite the immunological escape of the SARS-CoV-2 through the rapid rise of new mutants, most of the COVID-19 first generation vaccines, are composed only by the highly variable surface Spike antigen^7^, either expressed by recombinant non-replicating adenovirus^8–10^ or by messenger RNA^11,12^. Confirming that, nearly 56% of the 10 billion doses of vaccine delivered, correspond to the S spike protein non-replicating adenovirus and mRNA, while the whole virion inactivated (WVI) vaccines^13–15^, correspond to 44% of the delivered doses^7^.

The S protein is a surface predominant antigen involved in penetration of the virus into the host cell, therefore, it is the target of vaccine neutralizing antibodies^6^. However, its high mutation rate jeopardized the vaccine cross-efficacy against other SARS-CoV-2 mutants. Vaccination of large populations is time-consuming, laborious and expensive. While a vaccine against a new mutant is under scaling-up, clinical trial and registration, a new variant can emerge. Therefore, vaccines against coronavirus should be focused not only in the S highly variable antigen, but in other conserved antigens instead, that suffer less mutations, are present in all mutants and are thus capable of inducing cross-protection^16,17^. Once the virus is inside an APC, all its components, whether structural or not, have the same probability to be digested and presented to lymphocytes in order to generate an immune response. Thus, it is reasonable to suppose, that non-structural genes, which codify for vital enzymes responsible for the virus replication, would be more conserved and less variable than other proteins of the same virus or of the coronavirus family^17–20^. Conserved antigens should be therefore, the basis of second generation anti-COVID-19 vaccines^17–20^ as they could cross-protect against SARS, MERS, all the prevalent and even future SARS-CoV-2 variants.

Multiepitope protein vaccines optimize vaccine efficacy by joining the most immunogenic sequences of one or several antigens of a pathogen. A multivariant multiepitope vaccine expresses the most immunogenic conserved epitopes present in all variants of a pathogen, aiming to optimize their cross-efficacy. However, while several vaccines contain the Spike antigen^21–25^, fewer formulations combine epitopes of different antigens of the Wuhan Hu1 strain^26–28^ and, multiepitope multivariant vaccines using conserved epitopes of all variants have been less explored^7^.

In this investigation, we predicted in silico CD4^+^ and CD8^+^ T cell epitopes of the S, N, E and M structural proteins, and of the ORF1ab, ORF3a, ORF6, ORF7a, ORF7b ORF8 and ORF10 non-structural proteins of ten SARS-CoV-2 virus variants. Among them, we selected the epitopes that were: (1) promiscuous and had the highest binding affinity to HLA Class I and Class II molecules; (2) conserved in the Wuhan Hu1, Alpha, Beta, Gamma, Delta, and Omicron VOCs, and the Mµ, Zeta, Lambda, and R1 VOIs; and (3) that had higher population coverage. With these peptides, we designed a multiepitope universal vaccine potentially capable of generating cross-protection against all the human variants of SARS-CoV-2. In vivo vaccination tests will be needed in order to confirm this hypothesis.

## Results

### The Omicron VOC has an increased number of mutations, mainly in the S surface antigen

Mutations and deletions of the S, M, N, E, ORF1ab, ORF3a, ORF 6, ORF7a, ORF7b, ORF8 and ORF10 proteins, of the 9 SARS-CoV-2 variants were compared to the Wuhan-Hu1 strain (Table S1). S protein mutations represented 25%, 9%, 11%, 7%, 4%, 7%, 4% and 69% of the total mutations of variants Alpha, Beta, Gamma, Delta, Mu, Zeta, Lambda, R1 and Omicron, respectively. S protein deletions represented 100%, 40%, 0%, 0%, 0%, 0%, 100%, 70% and 6% of total deletions of variants Alpha, Beta, Gamma, Delta, Zeta and R1, Mu, Lambda and Omicron, respectively.

### Prediction and selection of the totally conserved and highly promiscuous CD4^+^ T cell epitopes of SARS-CoV-2 virus

For class II, the majority of endogenously bound ligands and epitopes are 12–20 residues in length, with 15-mers being generally the most common and most studied^20,21,24,25,29^. In the case of class II, because the binding groove is open, and the ligand can slide to assume optimal conformation, an exact size is not essential^30,31^.

The majority of class I ligands, to include both known T cell epitopes and endogenously bound peptides, are 9-mers.

CD4^+^ T cell epitopes were predicted from the sequences of the 109 proteins of all variants, using the IEDB Class II 27 alleles, considering only epitopes that bind to at least 10 alleles. The 100% conserved epitopes were then sorted according to a final score, described on “Methods” section. This final score is based on the number of bound alleles and on the percentile rank of the peptides. The higher the number of bound alleles and the lower the value of percentile rank, the strongest final score is and the epitope is a better HLA binder.

**Figure 1:**
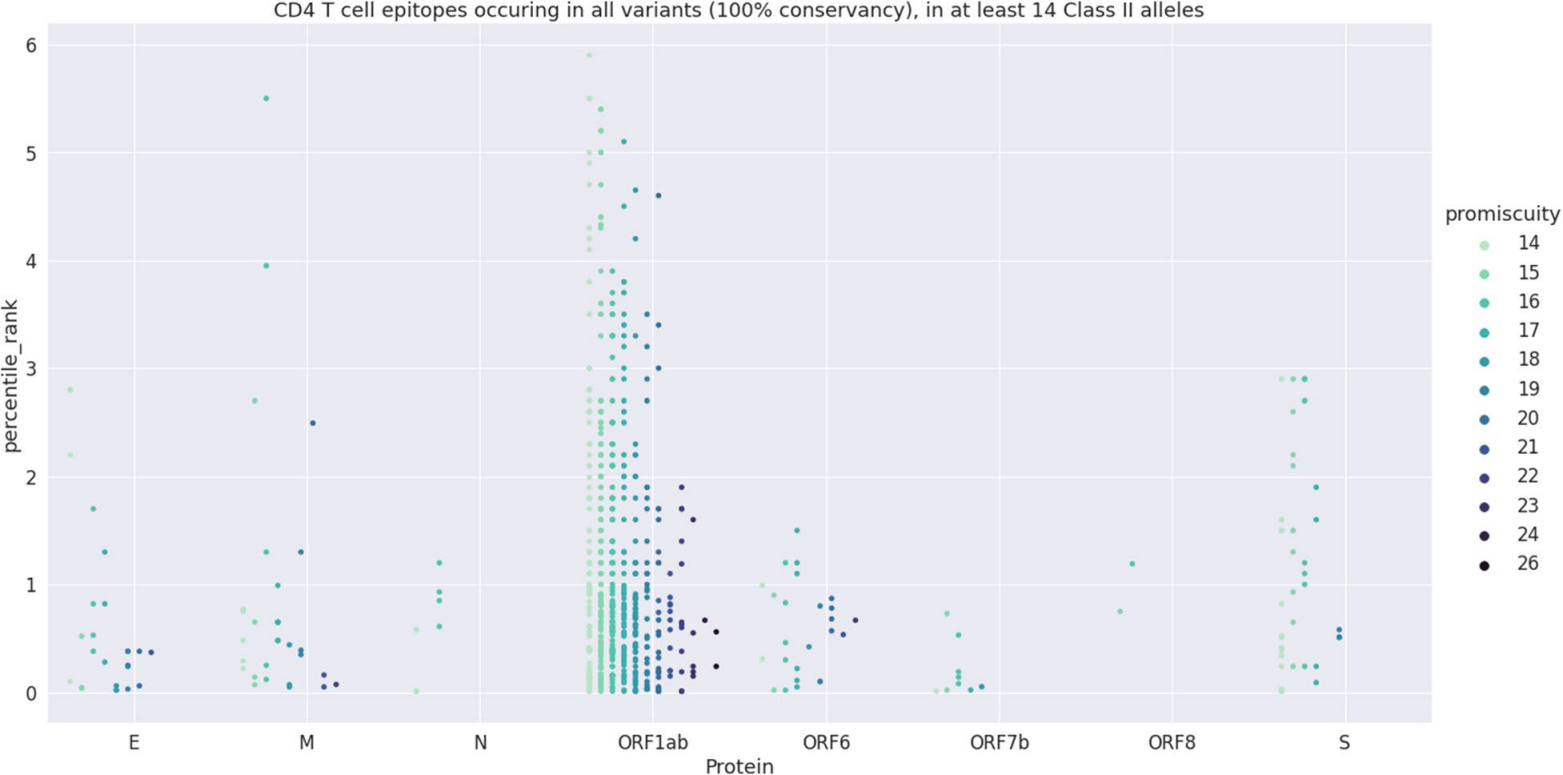
Prediction of total CD4^+^ epitopes. A prediction of 15-mers CD4 epitopes of the 109 proteins of all variants was carried out using the full set alleles of IEDB MHC Class II (27 alleles) and considering only the epitopes that bind to 10 or more alleles. We excluded from the list: (1) the non promiscuous epitopes that bind to less than 14 alleles, (2) the epitopes that were not conserved with 100% of identity among the 10 virus strains, and (3) the epitopes that were duplicated. These filters retrieved 676 CD4^+^ epitopes (Table S2) represented in this Category-plot which shows in the *y* axis the percentile rank values of all proteins that hold conserved epitopes and, in the *x* axis, the level of their promiscuity within a color-scale distribution. For each protein, the level of promiscuity increases from 14 to 26 HLA Class II alleles from left to right, along with the variation of colors from light green to dark violet color.

Bound alleles correlated negatively to percentile ranks (R = − 0.108; p = 0.000). Non-promiscuous epitopes that bind to less than 14 alleles; epitopes that were not conserved with 100% of identity among the 10 virus strains, and epitopes that were duplicated, were excluded from the list (Fig. 1). Percentile rank was negatively correlated to promiscuity (R = − 0.184; p = 1.343 × 10^–6^) and final score (R = − 0.8629; p < 0.0001). Final score and promiscuity correlated positively (R = 0.6739; p < 0.0001). No conserved promiscuous epitopes were predicted for ORF3a, ORF7a and ORF10. ORF1ab holds 537 epitopes that bind to a maximum of 26 among the 27 alleles (Table 1). ORF6 and M epitopes bind to a maximum of 22 alleles, and E epitopes bind to a maximum of 21 alleles. ORF8 and ORF7b contributed with less epitopes, which bind to a maximum of 18 and 16 alleles, respectively. Despite its high immunogenicity, 6.1% of the epitopes corresponded to the S protein, and bind to a maximum of only 19 alleles (Table 1).

**Table 1:** Predicted, promiscuous and totally conserved CD4^+^ and CD8^+^ epitopes of highest affinity.

Protein	Amino acids	CD4^+^ total epitopes			CD4^+^ overlapping epitopes			CD8^+^ total epitopes			
		Predicted	%	Alleles bound	Predicted	%	Alleles bound	Predicted	%	Alleles bound PR < 1%	Alleles bound PR < 0.5%
E	75	25	3.7	14–21	6	4.4	14–19	2	4.3	10	7
M	222	30	4.4	14–22	5	3.7	14–22	1	2.1	10	9
N	419	7	1.0	14–16	2	1.5	14–16	2	4.3	10–13	6–9
ORF1ab	7096	537	79.4	14–26	100	74.1	14–23	36	76.6	10–15	4–11
ORF6	61	25	3.7	14–22	7	5.2	14–20	0	0.0	–	–
ORF7b	43	9	1.3	14–18	2	1.5	15–16	0	0.0	–	–
ORF8	121	2	0.3	15–16	1	0.7	15	1	2.1	10	7
S	1273	41	6.1	14–19	12	8.9	14–19	5	10.6	11–12	2–8

Number of epitopes and number amino acids of each protein were correlated (p < 0.0001, r = 0.9902, r^2^ = 0.9806 (Table 1). The longest ORF1ab protein holds more epitopes. However, if normalized by the number of amino acids (right hand axis of Fig. S1a), proteins ORF6, E, ORF7b and M showed proportionally higher CD4^+^ epitopes densities.

The 676 CD4^+^ epitopes of the list were further filtered using the final scores in decreasing order (Table S3). Alternatively, filtering for 15-mers epitopes with overlapping of 10 amino acids retrieved 135 epitopes (Fig. S2 and Table S3). Promiscuity and percentile rank correlated negatively but, with no significance (R = − 0.079; p value = 0.363). The maximum number of alleles bound by the overlapping epitopes of E, ORF1ab, ORF6, ORF7b and ORF8 proteins (15–23) was also lower than those bound by total epitopes (Table 1) indicating that the prediction of total epitopes (Fig. 1), gave more robust results.

Epitope of 676 final-score filtered list that shared the same core, were combined into a longer final epitope that would include them all. Sixteen CD4^+^ novel epitopes, which combine sequences ranking up to position 84 of the list, with final scores ranging from 0.666 to 0.938, were selected (Table S4). Twelve of them belong to ORF1ab, two to E, one to M and one to ORF6 proteins. Their final scores ranged from 0.938 to 0.666, promiscuity from 26 to 19 alleles and percentile ranks from 0.01 to 1.19 (Table S4). A final prediction was ran for these 16 epitopes in the IEDB platform considering the full set Class II alleles (Table S5). A color-scale range from strong red color for high affinity binding (PR < 1%) to light pink for low affinity values (PR values close to 20%). The epitopes showed outstanding promiscuity values (16–22 alleles) and remarkably high affinity-binding values. Bindings with percentile ranks below 10% were observed for 71% of the epitope-allele combinations (Table S5). Sixteen of the 27 alleles (59%) were bound by at least 10 epitopes. The DPA1*02:01/DPB1*01:01, DRB1*01:01 and DRB1*04:05 alleles showed the maximal binding to all the 16 epitopes. DR, DP and DQ alleles of higher World population prevalence^32^, showed good affinity-binding capabilities with percentile rank values < 10% (Table S6).

### Prediction and selection of the totally conserved, promiscuous, strong and weak binders CD8^+^ T cell epitopes of SARS-CoV-2 virus

**Figure 2:**
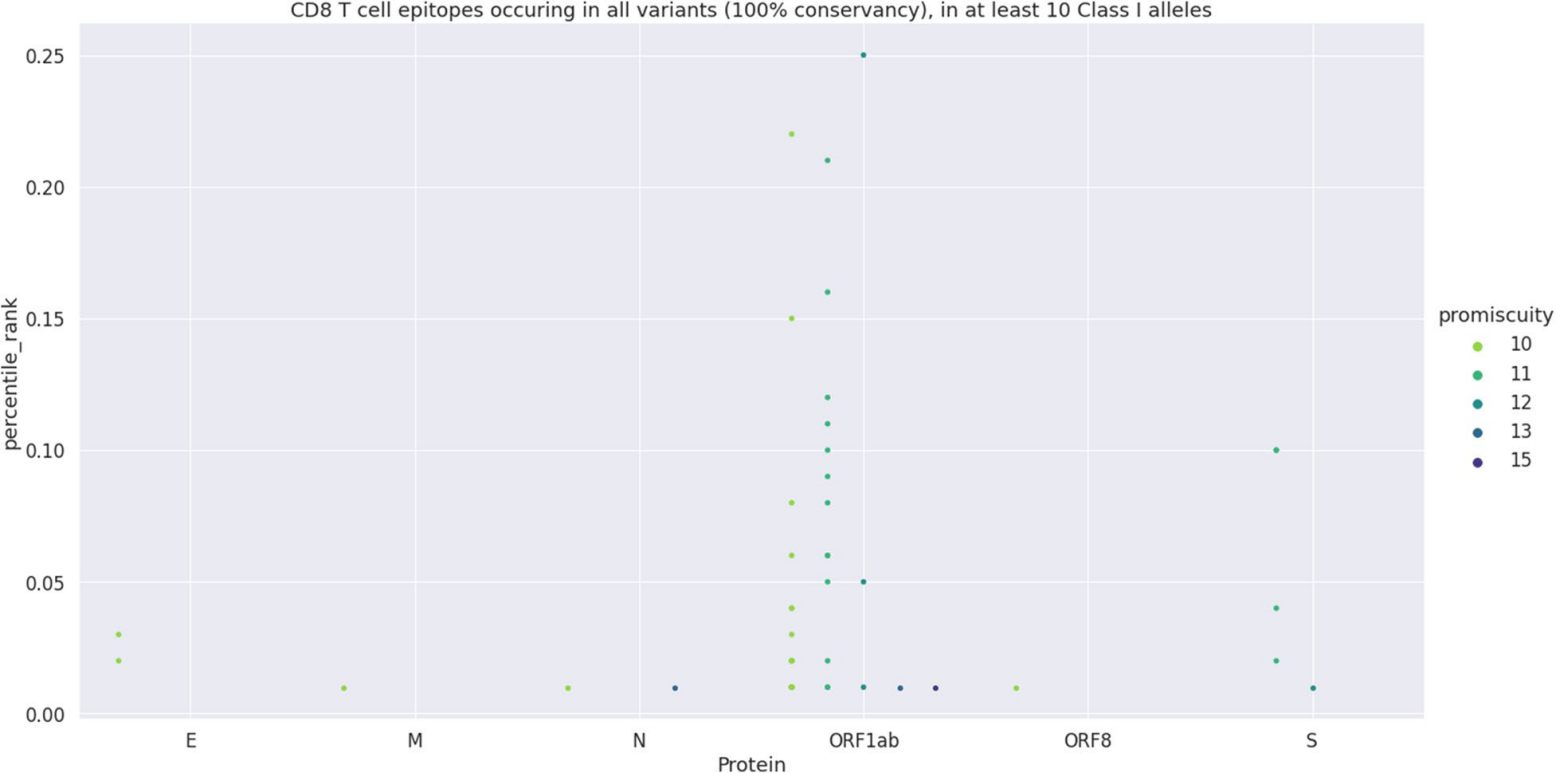
Prediction of total CD8^+^ epitopes. Category plot showing all predicted 9-mer CD8^+^ T cell epitopes (100% conserved, promiscuity of at least 10). Each dot corresponds to an epitope. The *y* axis shows the percentile rank values of all proteins that hold conserved epitopes and, in the *x* axis, the level of their promiscuity within a color-scale distribution. For each protein, the level of promiscuity increases from 10 to 15 HLA Class I alleles.

MHC Class I epitopes have a single alpha chain that impacts binding and because the binding grove is closed it can only accommodate short peptides (8–14 amino acids). The majority of class I ligands, to include both known T cell epitopes and endogenously bound peptides, are 9-mers^30,31^. In this investigation we used the default length of IEDB Class I predicting tool = 9 mer^20,21,24,25,29,33^. Epitopes were predicted from the sequences of 109 proteins of all variants, using the IEDB Class I 27 alleles. Epitopes that bind with percentile rank < 1%, to 10 or more alleles, are conserved with 100% of identity among the 10 virus strains, and are not duplicated, were selected. Forty-seven conserved epitopes that bind to 10–15 alleles with percentile rank < 1% were retrieved (Fig. 2). The 47 epitopes are considered weak binders of 10–15 alleles, for binding to them with PR < 1% but they also are strong binders to 2–11 alleles, with PR < 0.5% (Table S7). Results are shown in decreasing order of final scores and of number of alleles bound by the strong binders (PR < 0.5%). This method thus favored the strong binders (PR < 0.5%). Accordingly, final score correlated more to the promiscuity of strong binders (R = 0.9957; p < 0.0001) than of weak binders (R = 0.3088, p = 0.0347) and correlated negatively to percentile rank (R = − 0.3088, p = 0.3085). As such, peptide VVYRGTTTY, the first of the rank binds weakly to 12, but strongly to 11 alleles. In contrast, peptide KLFDRYFKY, the second in rank binds weakly to 15 and strongly to 10 alleles (Table S7). If two peptides have the same promiscuity (PR < 0.5%) we can tiebreak using the promiscuity (PR < 1%).

Most of the 47 epitopes belong to ORF1ab antigen (Table 1). S protein contributed with less CD8^+^ than CD4^+^ epitopes, and it was followed by the E and N and by the M and ORF8 antigens. Numbers of CD8^+^ epitopes and of amino acids of each protein (p < 0.0001, r = 0.9976, r^2^ = 0.9953) were correlated (Table 1). ORF1ab showed more CD8^+^ epitopes (Table 1). However, if normalized by the number of amino acids, protein E showed proportionally higher CD8^+^ epitope density (right hand axis of Fig. S1b).

**Figure 3:**
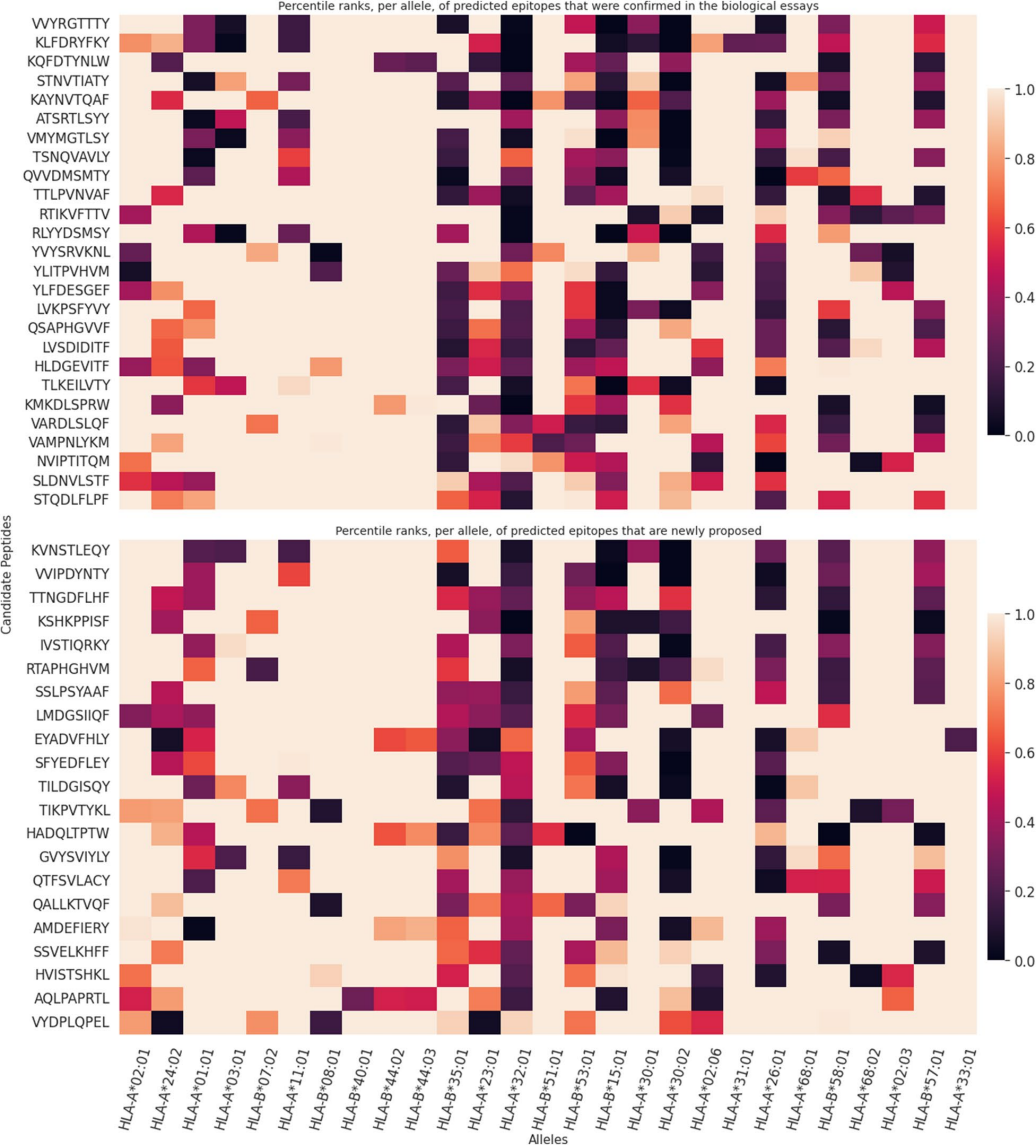
Heatmap of predicted candidate epitopes for CD8 T cells. Each peptide is represented in the y-axis. The values displayed in the heatmaps are the percentile ranks for each peptide, when binding each of the 27 most common Class I alleles (x-axis). Lower percentile ranks values, closer to zero, corresponding to darker colors ranging from light purple to dark purple/black, show stronger binder epitopes (PR < 0.5) for the purpose of this study. The values in light pink close to PR = 1.0, in contrast, are considered weak binder or non-binders epitopes. In addition, the alleles in x axis are sorted by their frequencies in Word Population in decreasing order from left to right. Furthermore, each row informs the degree of promiscuity of each respective epitope**.** Top heatmap includes the predicted epitopes that have already been confirmed by biological assays, and bottom heat map includes the predicted epitopes that are novel.

Figure 3 contains the heatmaps for the 47 predicted CD8^+^epitopes generated by the algorithm described in “Methods” section. In both heatmaps the epitopes are sorted by decreasing final score. The top heatmap shows 26 candidate peptides that have already been confirmed by immunological assays (12th column of the Table S7) as restricted to one to three different alleles. Eight of those 26 were considered dominant (epitopes labeled with yes in the 13th column of the Table S7)^34^. The bottom heatmap contains the other 21 novel candidate epitopes (Fig. 3).

The heatmaps allowed us to identify, 39 of the 47 Class I predicted sequences (Table S7), which are strong binders and link to the most frequent World Population alleles HLA-A*02:01(25.2%), HLA-A*24:02 (16.8%), HLA-A*01:01 (16.2%), HLA-A*03:01 (15.4%), HLA-A*07:02 (13.3%), HLA-A*11:01 (12.9%) and HLA-A*08:01 (11.5%)^32^ (Fig. 3). They show PR ranging from 0 to 0.5% in both, the top and bottom heatmaps, with colors ranging from light purple to purple/black in at least one epitope/allele combination (Fig. 3). In contrast, 7 epitopes from the top heatmap (LVKPSFYVY, LVSDIDITF, QSAPHGVVF, VARDLSLQF, VAMPNLYKM, NVIPTITQM and STQDLFLPF), and one epitope of the bottom heatmap (HVISTSHKL) were weak binders to the most frequent alleles, with PR values between 0.5 and 1% (Table S7) and colors ranging from red to light pink. For this reason, we excluded them from the list of candidates to compose the vaccine.

Among the 39 Class I strong binder epitopes listed according to their decreased final score, 20 of them are novel and 19 were described before (Table S8 epitopes with an asterisk). Eight of these 19 were considered as dominant (Table S7) by previous literature (revised in^34^. Furthermore, while no confirmed epitope was predicted to bind to the HLA-B*40:01 and HLA-A*33:01 alleles, the EYADVFHLY and AQLPAPRTL novel epitopes, in fact, did.

### World population coverage of the T cell epitopes

The coverage value for the whole Class II epitopes is 99.45%. The average number of epitope hits/HLA combinations recognized by the population (average hit) is 27.51 and the minimum number of epitope hits/HLA combinations recognized by the population (Pc 90^C^) is 12.99 (Fig. S3). Remarkably, the individual World population coverage percent for each epitope ranged from 95.75 to 62.88% (Table S4). Ten among the 16 epitopes show coverages above 90%, 3 above 86%, 2 above 70% and one, above 60%. Moreover, considering a percentile rank < 0.5%, the population coverage for the Class I strong binders was 98.09%, the average hit = 24.96% and the pc90^c^ = 6.79 (Fig. S4a). Considering a percentile rank < 1%, the population coverage for the weak binders was 98.55%, the average hit = 40.77% the pc90^c^ = 14.55 (Fig. S4b). Population coverages of strong binder epitopes (PR = 0.5%) (Table S7) are correlated with the final scores (p < 0.0001, r = 0.6712, r^2^ = 0.4505) and with the number of bound alleles (p < 0.0001, r = 0.6870, r^2^ = 0.4720).

### Epitope-conservancy analysis

The epitope conservation analysis generated two types of data files, each for CD4 and CD8, which are included in the Supplementary Materials. One of the data file types was a table providing the conservation ratio of each epitope among all genomes in the dataset and among all genomes of each variant clade, with values ranging between 0 and 1 after applying formula (1) in columns 6 and (2) in columns 7 to 20 of Tables S9 and S10, respectively. The second data file type provides the raw fraction, total number of conserved genomes/total number of genomes, for each epitope among the entire collection of genomes (columns 20) and among each clade (columns 6 to 19) of Tables S11 and S12, respectively, thus capturing how many genomes made up a WHO clade in the denominator. WHO clades such as Omicron and Delta comprised of far more genomes to the total genome analysis than clades such as Eta and Kappa; hence, a raw fraction of conservation offered an alternative perspective regarding the level of conservation.

**Figure 4:**
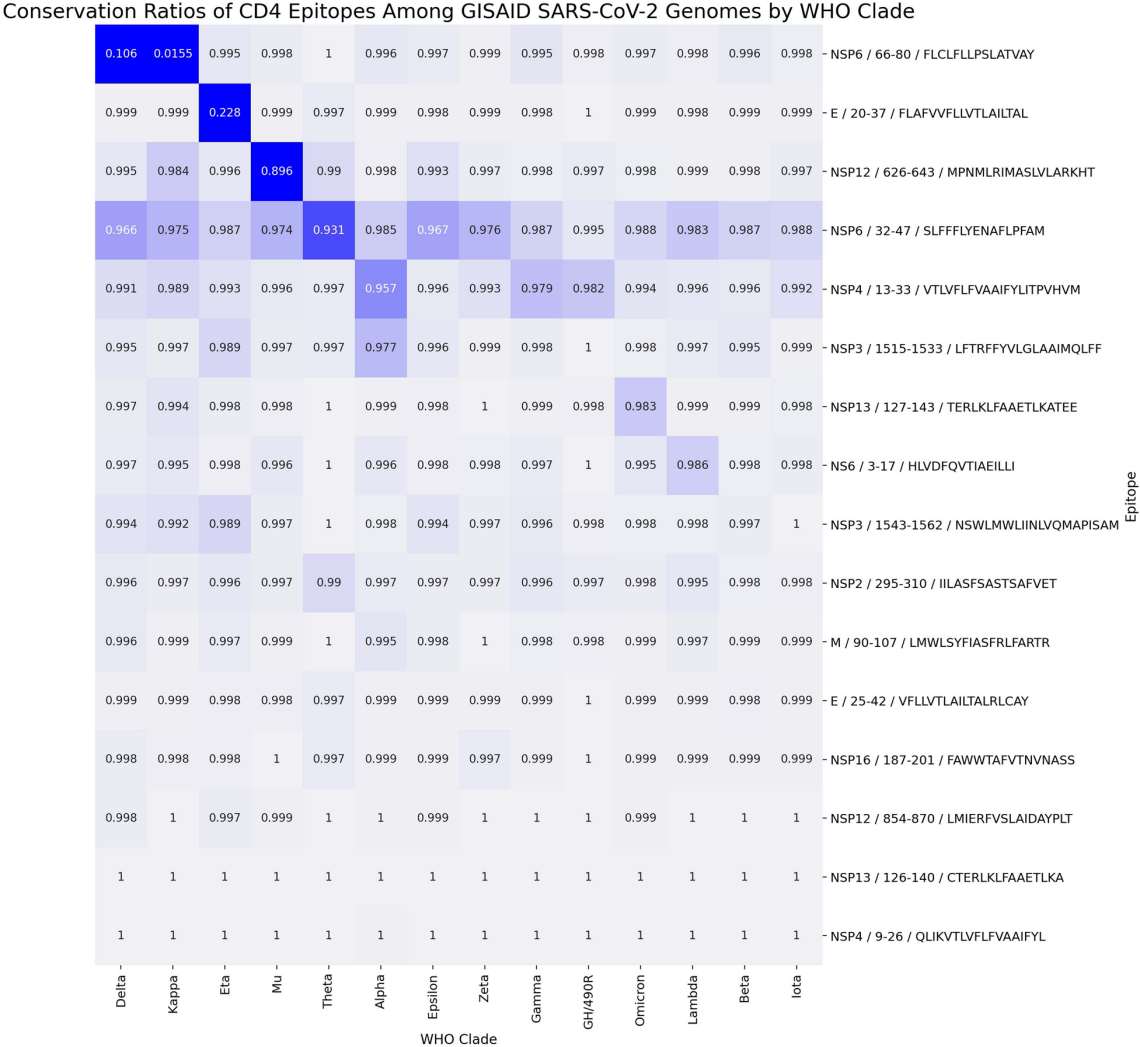
CD4 T cell epitope conservation. The conservation of SARS-CoV-2 CD4 epitopes broken down by WHO clade rounded to the third significant digit is shown. The color scale is cutoff at a 0.90 conservation ratio such that everything less than 0.90 is assigned the same dark color and everything above 0.90 scaled with different colors. Each epitope on the y-axis is labeled as [protein]/[amino acid start position]-[amino acid end position]/[epitope sequence]. The order of WHO clades was determined by hierarchical clustering.

All but one of the CD4 epitopes showed a level of conservation greater than 97% among all genomes (Fig. 4 and Tables S9–S11). The one epitope with lower conservation ratio (62% conserved among all genomes) was from NSP6 position 66–80 (FLCLFLLPSLATVAY). This NSP6: 66–80 epitope was only 11% conserved among all Delta genomes and 1.5% conserved among all Kappa genomes due to the T77A susbtitution; all other clades showed greater than 99% conservation at this epitope. The E: 20–37 CD4 epitope (FLAFVVFLLVTLAILTAL) was only 23% conserved in the Eta clade but was greater than 99% conserved overall.

**Figure 5:**
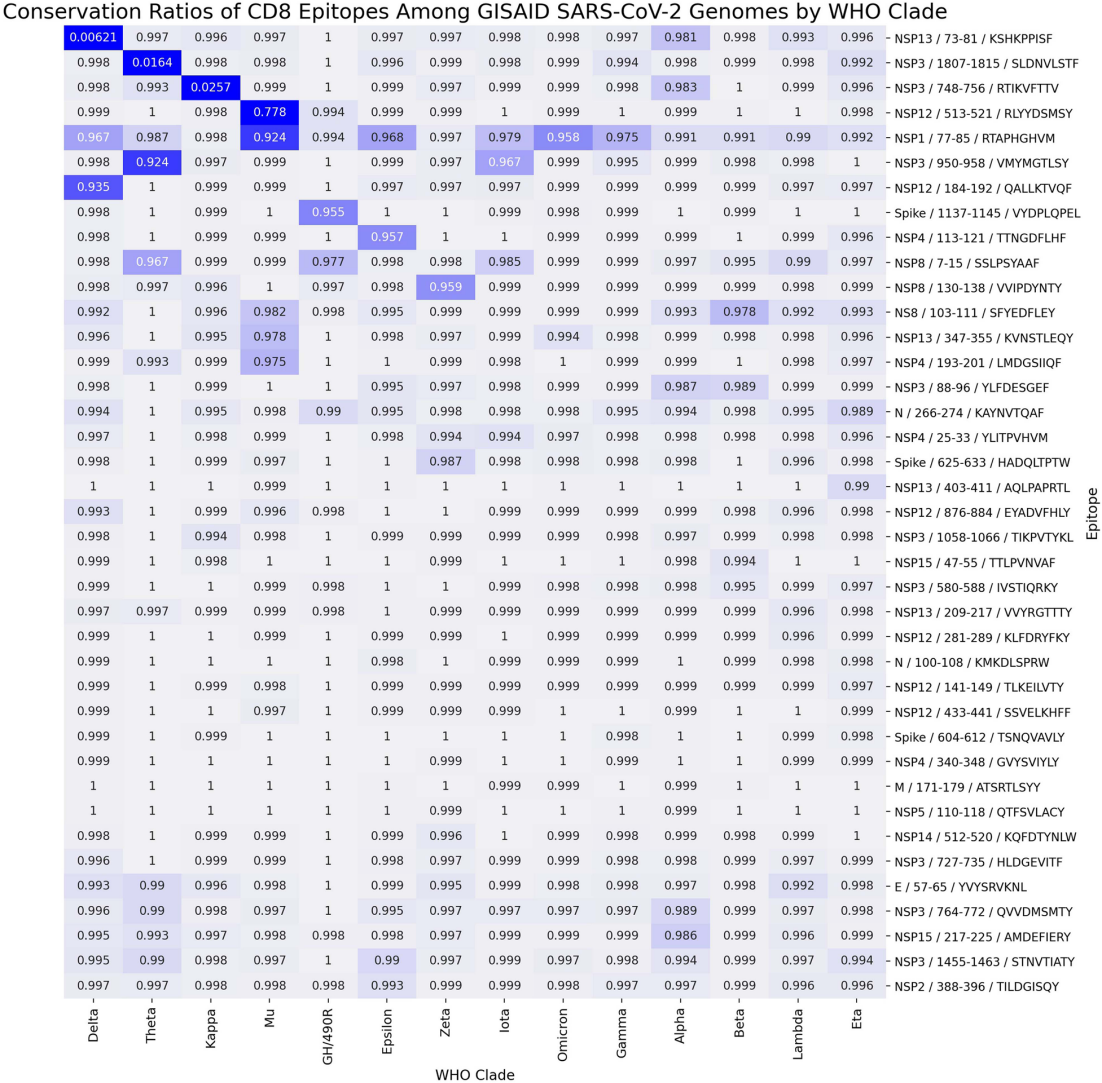
CD8 T cell epitope conservation. The conservation of SARS-CoV-2 CD8 epitopes broken down by WHO clade rounded to the third significant digit is shown. The color scale is cutoff at a 0.90 conservation ratio such that everything less than 0.90 is assigned the same dark color and everything above 0.90 scaled with different colors. Each epitope on the y-axis is labeled as [protein]/[amino acid start position]-[amino acid end position]/[epitope sequence]. The order of WHO clades was determined by hierarchical clustering.

All but one of the CD8 epitopes showed a level of conservation greater than 96% among all genomes (Fig. 5 and Tables S10–S12) and the vast majority greater than 99%. The one epitope with lower conservation ratio (58% conserved among all genomes) was from NSP13 (helicase) position 73–81 (KSHKPPISF), largely due to the presence of the mutation P77L substitution resulting in a 0.6% conservation level in the Delta clade. This epitope was greater than 98% conserved in all other clades. Other epitopes with clade-specific lower conservation values are NSP3: 1807–1815 (SLDNVLSTF) with only 1.6% conservation in Theta, NSP3: 748–756 (RTIKVFTTV) with only 2.6% conservancy in Kappa, and NSP12: 513–521 (RLYYDSMSY) with 78% conservation in Mu.

### Composition, physicochemical and absence of toxicity of the multiepitope vaccine

**Figure 6:**
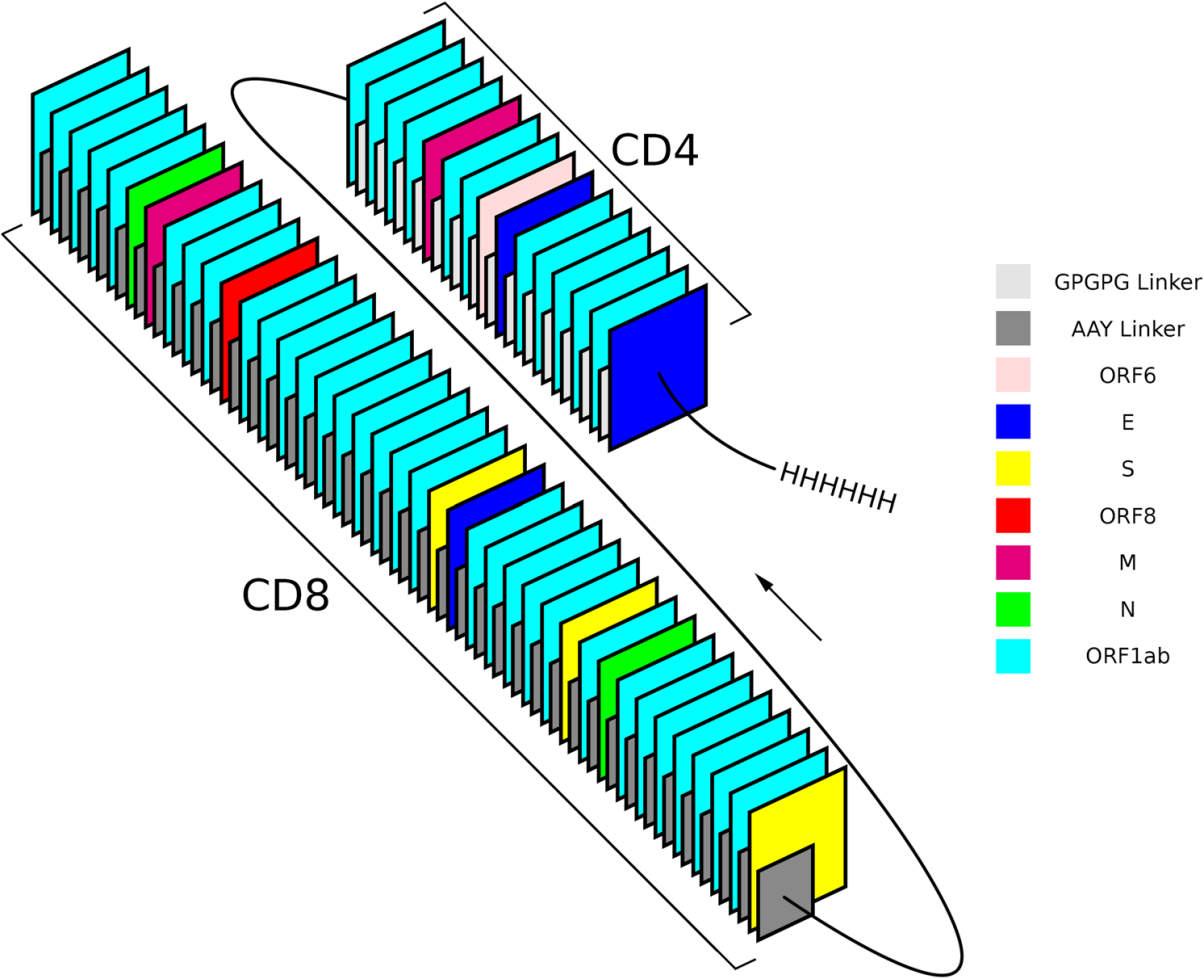
Combination of CD4^+^, CD8^+^ conserved T cell epitopes and linkers in the multiepitope multivariant vaccine against SARS-CoV-2. 819 amino-acid residues organized in 39 CD8^+^ epitopes, joined by AAY linkers (dark grey), at the N-terminal region, followed by 16 CD4^+^ epitopes joined by GPGPG linkers (light grey), at the C-terminal, ended by a 6-HIS tail. The AAY and GPGPG spacers are included to avoid the formation of junctional epitopes^35^. The epitopes belong to the ORF6 (pink), E (blue), S (yellow), ORF8 (red), M (magenta), N (green) and ORF1ab (cyan) SARS-CoV-2 proteins.

The multiepitope vaccine holds at its N terminal, 39 Class I strong binder epitopes (Table S8), joined by AAY sequences (Fig. 6), followed by the 16 Class II epitopes (Table S4) joined by GPGPG linkers^35^ and ends with a 6 HIS-tail at its C-terminal which will allow one-step purification of the multiepitope vaccine by Ni^2+^ affinity columns. The inclusion of the repetitive regions, called spacers or linkers, intends to avoid the formation of faulty neoepitopes or junctional epitopes. Even if the epitopes of the pathogen are very well predicted and synthetized, the hydrolytic enzymes of dendritic cells or macrophages cells that ingest the vaccine, may not cut the protein exactly on their flanking amino acids in order to release the predicted sequences. Instead, the cuts between wrong amino acids will release disrupted epitopes with destroyed immunogenicity, i.e. junctional epitopes or neoepitopes^35^. As the G and P residues are not normally found in anchor positions, the introduction of a GPGPG spacer between CD4 epitopes would have a higher probability of preventing the generation of junctional epitopes^35^. Simultaneously, it was observed that the addition of the AAY spacers improved the responses to Class I epitopes of multiepitope vaccine against tuberculosis^36^, tumors^37^ and *Staphylococcus aureus*^38^. Since then, all the designs of multiepitope vaccines use the GPGPG spacers between CD4 sequences and the AAA, AAY spacer for CD8 sequences^21,25,27,37,39,40^.

Epitopes in the SARS-CoV-2 multiepitope vaccine are organized in decreasing order of final scores. Furthermore, the expression of a 6× His-tag tail on the C-terminal of the multiepitope protein will allow its one-step purification by Ni^2+^ affinity columns^41^.

The protein has 825 amino acids, a molecular weight of 90,840.18 D and is basic (pI = 8.2). Its instability index of 27.72 (below 40) classifies it as stable. It has 100 h of estimated half-life in mammalian reticulocytes in vitro, more than 20 h in yeast in vivo and more than 10 h in *Escherichia coli *in vivo. Its aliphatic index is 93.24. Its average hydropathic index (GRAVY) of 0.446 classifies it as mildly hydrophobic. The multiepitope vaccine was predicted to be non-toxic by the PSI-BLAST BTXpred Server tool (https://webs.iiitd.edu.in/cgibin/btxpred/btx_main.pl).

### Secondary structure prediction

PSIPRED tool revealed in the multiepitope vaccine an estimated 63% of α-helix, 9% of β-strand and 27% coil (Fig. S5). One amino acid (K 361) is predicted as a putative domain boundary. Raptor X tool disclosed that, regarding the solvent accessibility of the protein, 28% of its residues are in an exposed region, 24% are in a moderately exposed region, 47% are buried and only 2% are predicted as in a disordered position.

### Tertiary structure modeling and interactions with the TLR4 receptor

**Figure 7:**
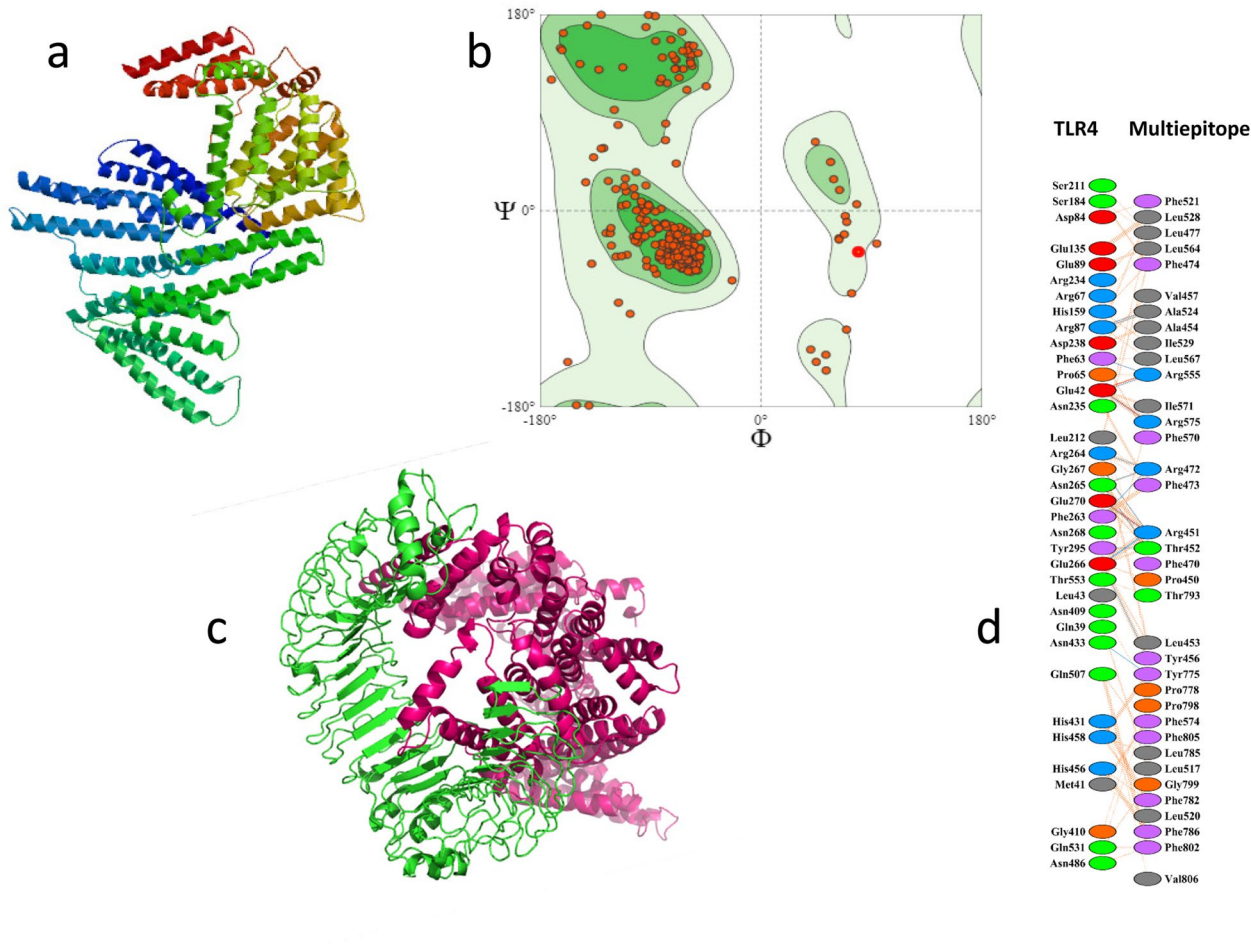
Tertiary structure of the multiepitope vaccine and its interactions with the TLR4 human receptor. (**a**) Three dimensional structure of the multiepitope conserved SARS-CoV-2 vaccine obtained with the Robetta software. Model refinement by Swiss Model and PROCHEK-PDBSUM indicated the Robetta´s model 4, for its best scores and good agreement with the usual protein models. (**b**) Swiss Model Ramachandran plot validation of the vaccine model. (**c**) interaction of the vaccine model (Pink) with the TLR4 (Lemon green). (**d**) interactions of the TLR4 (PDB ID: 2Z63) and the ClusPro 2.0 model 2 of the multiepitope vaccine as disclosed by the PDBSUM tool.

Robetta tool provided five 3D models of the vaccine. Model refinement by Swiss Model and PROCHEK-PDBSUM indicated model 4, for its best scores and good agreement with the usual protein models (Fig. 7a). In fact, Model 4 (Fig. 7a) received the best Q mean Z score (− 1.90), the highest Qmean Disco Global (0.39 ± 0.05), the highest GMQE (0.34) and the solvation value = 1.06 of the Swiss Model tool. QMEAN of the Swiss Model tool is an estimator known as z-score. When the value is close to 0, the model is reliable and, therefore, there is a good agreement between the model and the experimental structures. Furthermore, the model 4 also received the highest G factor = 0.55 of the PROCHEK-PDBSUM tool. The G factor gives an estimate of how unusual the model is (− 0.5 unusual, − 1 highly unusual). Furthermore, the model 4 also received the highest G factor = 0.55 of the PROCHEK- PDBSUM tool (Fig. 7a). Model 4 Ramachandran graph (Fig. 7b) disclosed 94.97% of the residues in a favored position, 0.74% outliers and 0.47% rotamers and was used for molecular docking studies. Clus Pro 2.0 indicated model 2 of the vaccine complex with TLR4 (Fig. 7c) as the best docked, showing cluster size = 33 and lowest interaction energy scores of − 1526.2 (Table S13). PDBSUM disclosed 36 residues of the TLR4, which interact with 36 residues of the vaccine through 3 salt bridges, 16 hydrogen bonds and 270 non-bonded contacts (Fig. 7d). The contact interface area was 2095 Å^2^ of the TLR4 and 2106 Å^2^ of the multiepitope vaccine.

### Cloning, N-glycosylation and codon optimization

**Figure 8:**
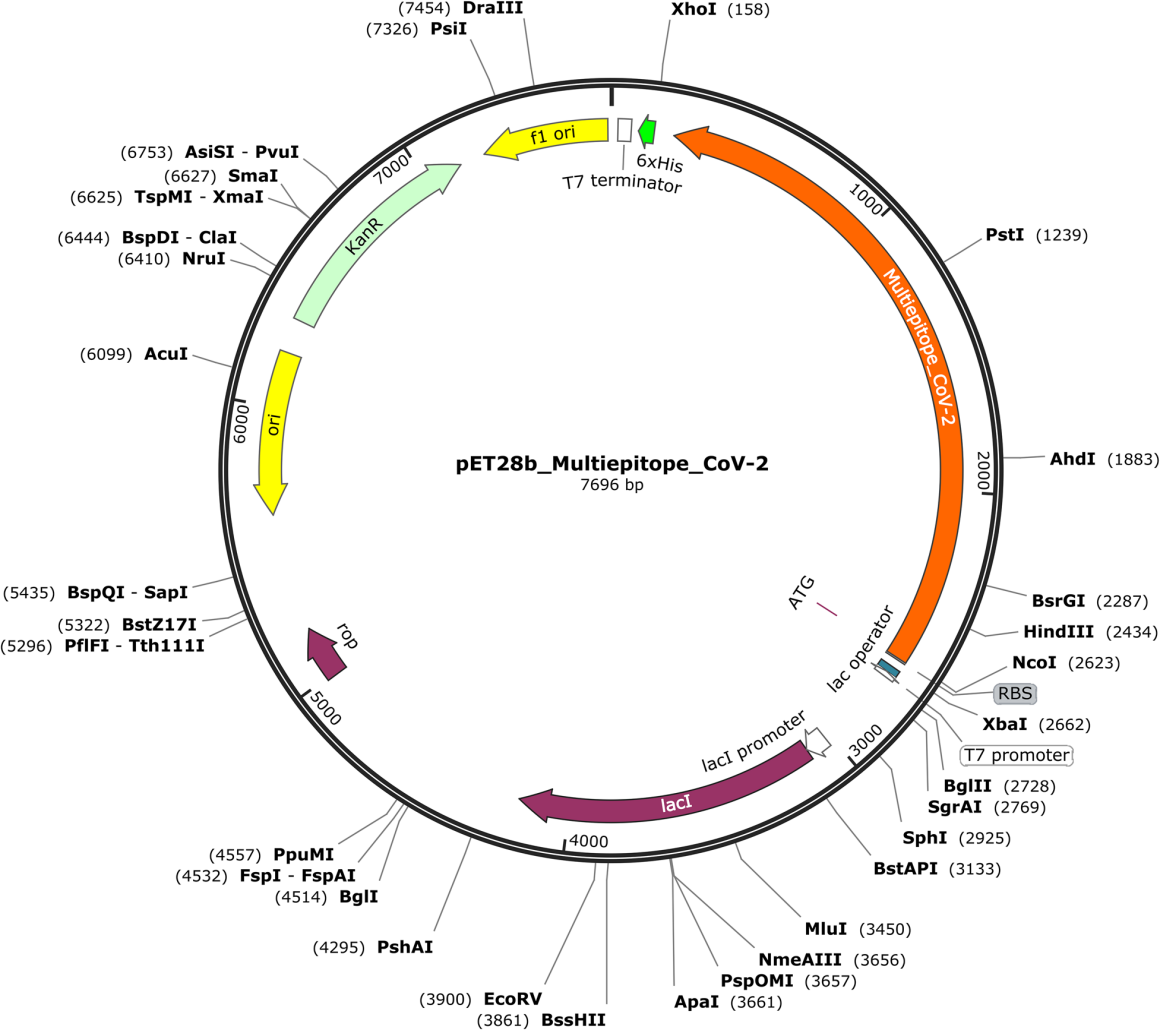
In silico cloning of the Multiepitope vaccine gene in a restriction cloning vector pET28b (+) in *E.coli* host. The cDNA nucleotide sequence of the vaccine was obtained with optimized codons for *Escherichia coli* K12 using the Java codon adaptation index tool. The GC content was 53.41%, while the normal range is 40–60%. Furthermore, the codon optimization index (CAI) value was 1.0. CAI higher than 0.5  is considered acceptable and indicates the high expression of the vector pET28b +. Here, the red areas indicate the Multiepitope vaccine DNA sequence, and the black areas represent the expression vector, pET28b (+).

The vaccine cDNA nucleotide sequence with optimized codons for *Escherichia coli* K12 was cloned into a pET28b + plasmid, between the *NcoI* and *XhoI* restriction sites with a 6 His-tag at its C-terminal (Fig. 8). The NetNGlyc—1.0 tool detected three N residues (asparagine) in positions 26, 51 and 64 of the multiepitope protein. However, the lack of peptide signals revealed that the protein might not be glycosylated in vivo even though it contains these potential motifs.

### Immune simulation

**Figure 9:**
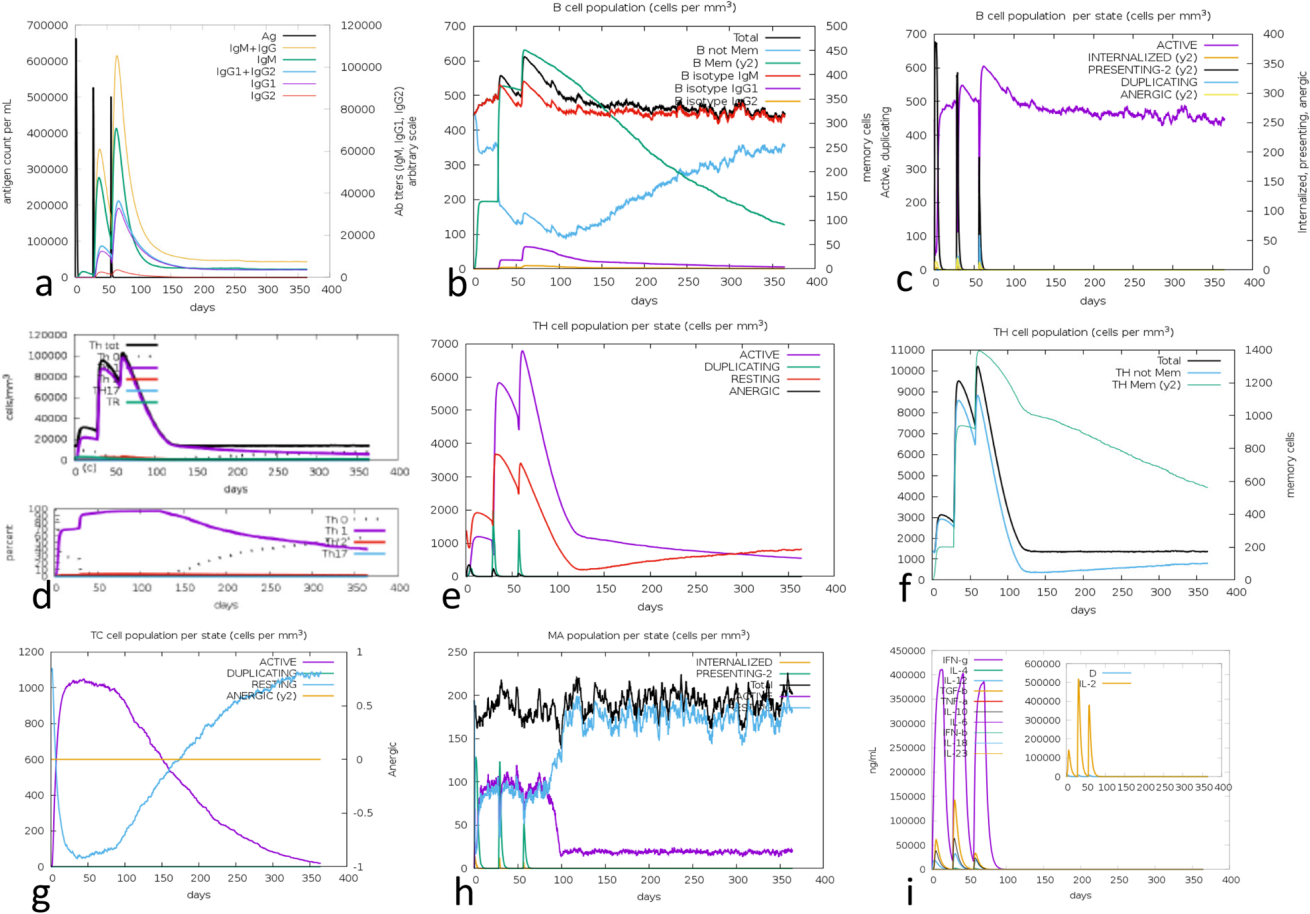
In silico immune stimulation analysis. The C-ImmSim tool simulation of the immune response promoted by the multiepitope was performed without the use of adjuvant. The tool offers only LPS as the option of adjuvant and we prefer instead, the QS21 saponin of *Quillaja saponaria* Molina. These two adjuvants probably use different mechanisms. Therefore, the simulation of the immune response was performed without the use of adjuvant. (**a**) Antigen and antibody response. (**b**) B cell counts. (**c**) B cell population per stat. (**d**) Th cell counts. (**e**) Th cell population per state. (**f**) Th memory T cells. (**g**) T cytotoxic cells per state. (**h**) Macrophage population per state. (**i**) Levels of cytokines.

To estimate the impact of the use of three injections of the multiepitope vaccine on the human immune system we used the C-ImmSim software. For host HLA selection the HLA-A*02:01, HLA-A*24:02, HLA-B*35:01, HLA-B*40:02, DRB1*07:01 and DRB1*15:01 were considered. To perform the immune stimulation, three injections at interval of 28 days were administered using 1000 multiepitope vaccine molecules per dose. Random seed, simulation volume and simulation steps were fixed as 12,345, 10 µl and 1095 (period analyzed 365 days), respectively. The injections were thus given at day 0, 28 and 56, which correspond to time steps of 1, 84 and 168 since one day is equivalent to 3 periods of 8 h. A sequence of 46 Alanine residues was used as a negative control and the sequence of the Full = Probable citrate synthase, mitochondrial; Flags: Precursor UniProtKB/Swiss-Prot: A4H9H8.1 was used a positive control. IgM and IgM + IgG primary and secondary humoral responses with predominant IgG1 and a mild enhance of IgG2 antibodies (Fig. 9a,b) were observed. The B cell population remained high and active along the first year (Fig. 9b,c); including B cell memory cells (Fig. 9b). Th1 T cell counts increased after injections (Fig. 9d,e) and Th memory T cells were sustained along the whole year (Fig. 9f). In contrast, cytotoxic T cell counts decreased rapidly (Fig. 9g). Increased macrophage activity was also observed (Fig. 9h). Increases of IL-2, followed in magnitude by IFN-γ and IL-12 levels confirmed the raise a Th1 pro-inflammatory response, while lower levels of TGF-β and IL-10 suggested a mild regulatory response (Fig. 9i). The vaccine did not promote the secretion of IL-6 (Fig. 9i). The negative control did not generate any antibody response (Fig. S6a) and its B cell population was active only for the IgM isotype (Fig. S6b). No Th1, Th2 or Th17 responses were detected (Fig. S6d) and only very low Th memory (Fig. S6e) and resting T cell (Fig. S6f) but no resting macrophage responses (Fig. S6h) were observed. Furthermore, peaks of IFN-γ were detected only after injections while no other secreted cytokine were observed (Fig. S6i). In contrast, the immune response generated by the positive control (A4H9H8.1 protein) was very similar to that of the multiepitope vaccine for all variables (Fig. S7a–h), differing only by its higher IL-2 secretion, after the second and third protein injections (Fig. S7i).

## Discussion

A vaccine against a highly mutating virus should contain a pool of antigens. If mutations occur in a single antigen, the protection generated against the remaining could circumvent the immune escape and guarantee the control infection, avoiding the disease^18^. In this way, the currently used S-protein vaccines, not only did not block the virus transmission and the re-infection by multiple variants, but also possibly contributed to the positive selection of variants such as the Omicron, which holds a completely different S antigen. In fact, most of the currently used COVID-19 vaccines are composed of the S antigen^7^, in which mutation rate ranged from 4%, in Zeta to 69%, in Omicron variant. These facts confirm the urgent need of development of a universal multivariant vaccine based on conserved multiple antigens of SARS-CoV-2 virus.

Until present, however, the multivariant conserved-epitope approach was less explored^42^. Our study is the first of them to take into consideration ten SARS-CoV-2 human variants including the Omicron VOC. Before the Omicron VOC, Prakash et al.^20^ in 2021, identified epitopes highly conserved among SARS-CoV-2 genomes, CoVs responsible for epidemics of common cold; SL-CoVs isolated from bats, pangolins, civet cats and MERS isolated from camels. Other multiepitope vaccines used only 81–100% conserved epitopes of the S, M, N and E^43^, or epitopes of the S, M and E antigens with almost 100% of conservancy^42^. Furthermore, while most recent investigations used predicted overlapping epitopes with excellent outcome^34,44,45^, in our study, the non-overlapping CD4^+^ epitope prediction showed more robust results. Finally, while many studies used for predictions a selected group of the human HLA Class II and/or Class I alleles^17,20,21,25,34,46^**,** we used the full IEDB reference list.

While the number of genomes and antigens investigated for retrieving of the epitopes might be a concern, most multiepitope proposed vaccines used only epitopes of the S protein of the Wuhan-Hu1 reference genome^22–24^ or of a non-disclosed variant^25^. Less vaccines used epitopes of the S, N, E^27^ or of the S, N, E and M proteins, also of the Wuhan Hu1 reference strain^21,43^ or of one SARS-CoV-2 and one SARS-CoV strains of China^28^.

On the other hand, less multiepitope vaccines are composed of epitopes conserved in multiple isolates. Similar to our study, Prakash et al.^20^ investigated the conserved epitopes of 10 proteins although retrieved from 81,963 genomes of GISAID bank, with disregard of their respective variant. Since they published their study in April 2021, they could not include any sample of the Omicron variant. Although the conservation approach in that study is very robust, because it is based on the analysis of many isolates, the epitope predictions were only run for 5 alleles of DRB1 for CD4 and 5 alleles for HLA-A. In contrast, in our study, we used the 27 alleles of DR, DQ and DP, and the 27 alleles of the HLA-A and HLA-B genes, which certainly represents a more extensive world populational coverage approach. Furthermore, Al Zamane et al.^42^ studied only the epitopes of S, M and E proteins that were however, present in 128 isolates from Bangladesh and 110 from other afflicted countries, while Rajput et al.^26^ retrieved the common epitopes of 92 genomes of SARS-CoV-2.

To the best of our knowledge, none of the 16 CD4^+^ epitopes identified in our study were described before^34^. However, the epitope MPNMLRIMASLVLARKHT_5018-5034_ of our list includes the sequence PNMLRIMASLVLARK_5019–5033_ described as a common CD4^+^ epitope, conserved in humans, bats, pangolin and camels^20^. We now showed that this epitope is also present in the ten SARS-CoV-2 human variants, including the Omicron. Furthermore, the epitope FLAFVVFLLVTLAILTAL_20–27_ of our list, partially overlaps with the epitope LLAFLAFVVFLLVTLA_18–32_ described by Singh et al.^21^ and with the epitopes LLVTLAILTALRLCA and LVTLAILTALRLCAY, previously shown in the literature^47–49^ with experimental evidence^48^. However, both have been shown to be very unrepresentative for countries like Brazil, being good binders just for one of the alleles with a frequency over 5%^50^. Interestingly, the peptide LIVNSVLLFLAFVVFLLVTLAILTALRLCAY described by Tiloca et al.^47^, was also present in SARS-CoV, Pangolin CoV, Bat CoV, SARS-CoV-2, Dromedarius CoV, Camel CoV, Canine CoV, Bovine, CoV, H- Enteric CoV and Avian CoV.

Prakash et al.^20^ analized 81,963 human sequences of SARS-CoV-2 collected from the GISAID and NCBI databanks on 27th August, 2020. They used a conservancy approach for linear epitopes with a sequence identity threshold set at ≥ 50%. The CD8 and CD4 epitopes that showed ≥ 50% conservancy in at least two human and two animals SARS-CoV strains (from bat/civet/pangolin/camel) were selected as candidates. Although conservancy was 100% among human SARS-CoV-2 strains, identity to 4 major common cold Coronavirus, Coronavirus from previous outbreaks and SL-CoV-2 from bats, civet, camels and pangolins was much lower^20^. In contrast, in our study, we identified 16 CD4 and 39 CD8 epitopes that were 100% conserved in 10 proteins of each one of the Wuhan-Hu1, Alpha, Beta, Gamma, Delta, Omicron, Mµ, Zeta, Lambda and R1 variants. While Prakash et al.^20^ studied the epitope-conservancy among 81,963 GISAID sequences, our conservancy approach was far more comprehensive and included 3,630,666 genomes from 16 clades: the 10 that were used for the identification of the conserved epitopes (Wuhan-Hu1, Alpha, Beta, Gamma, Delta, Omicron, Mµ, Zeta, Lambda and R1) and 6 more clades (Epsilon, Lota, Theta, Eta, Kappa and GH490 R). These genomes were also collected during a longer period, from 01 November 2019 up to August 2022.

High levels of epitope conservation were detected. Fifteen out of 16 CD4 selected epitopes showed levels of conservation ranging from 97 to 100% among all genomes, and 38 out of 39 of the CD8 epitopes exhibited level of conservation greater than 96% among all genomes, with the vast majority greater than 99%. These findings support the potential efficacy of our multivariant multiepitope vaccine in generation of cross-protection against infections by virus of different human SARS-CoV-2 clades.

The one CD4 epitope with 67% conservation in all genomes, called NSP6: 66–80 (FLCLFLLPSLATVAY) was only 11% conserved among all Delta genomes and 1.5% conserved among all Kappa genomes due to a T77A substitution; all other clades showed greater than 99% conservation at this epitope. Moreover, the one CD8 epitope with lower conservation ratio (58% conserved among all genomes) was from NSP13 (helicase) position 73–81 (KSHKPPISF), largely due to the presence of the mutation P77L substitution resulting in a 0.6% conservation level in the Delta clade. This epitope was greater than 98% conserved in all other clades. These two epitopes were however, 100% conserved in the sequence of ORF1ab of the Delta genome used for our epitope prediction (NCBI OK570404.1). Interestingly, while the two Delta mutations of interest NSP6: T77A and NSP13: P77L officially became positively selected on July 2021 and May 2021, respectively^51^, the Delta genome OK570404.1, used for epitope prediction was collected on June 24, 2021 [https://www.ncbi.nlm.nih.gov/nuccore/OK570404.1]. The decline in conservancy of these two epitopes is most certainly a result of the growth and evolution of the Delta clade.

Considering the importance of the Spike protein to the humoral response, it would be expected that it would be present more in CD4 than in CD8 T cell epitopes. In fact, the adaptive immune system can provide strong and durable cellular and humoral immunity to SARS-CoV-2, through coordinated T and B cell responses. CD4+ helper T cells are very important because they differentiate into T helper type 1 (Th1) cells, to stimulate phagocytes and cytotoxic CD8+ T cells, and into T follicular helper (Tfh) cells to promote high affinity and long-lived antibody responses by B cells in germinal center (GC) reactions^52^. Individuals with COVID-19 who did not require hospitalization had a greater percentage of circulating Tfh (cTfh) and Th1 cells among SARS-CoV-2-specific cells^51^.

In contrast, we showed that the S protein contributed to the multiepitope vaccine with more CD8 than CD4 epitopes. In fact, our prediction did not retrieved any CD4 epitope of the S protein. These results mean that the Spike protein does not have any CD4 epitope 100% conserved among the 10 variants, that has a high final score. This is why probably, the vaccines based on the S protein of Wuhan-Hu1 variant^8–12^, in spite of generating strong humoral responses against the spike protein they do not efficient cross-protect against the other SARS-CoV-2 variants. In fact, we showed that the spike protein is one of the most mutating antigens of SARS-CoV-2.

In Italian COVID-19 patients, the HLA-DRB1*15:01, DQB1*06:02 alleles have been associated to severity or extreme severity^53^, and the allele HLA-DRB1*08 to mortality^54^. In our study, 10 out of 16 Class II epitopes bind to HLA-DRB1*15:01, 8 epitopes to the DQA1*01:02/DQB1*06:02 and 7 epitopes bind to HLA-DRB1*08:02 alleles, with high affinity, indicating that our vaccine could protect more susceptible individuals.

Furthermore, the frequency of HLA-DRB1*04:01 was higher in asymptomatic individuals, suggesting its association with resistance; and the frequency of the haplotype DQA1*01:01-DQB1*05:01-DRB1*01:01 was lower in the asymptomatic group, suggesting its correlation to COVID-19 severity^55^. In addition, the frequency of the DPB1*04:01 was lower in Chinese COVID-19 patients^56^ suggesting correlation with resistance to the disease. In our prediction, 10 epitopes bind to DRB1*04:01, 9 to DQA1*01:01/DQB1*05:01, 12 to DRB1*01:01 and 10 to DPA1*01:03/DPB1*04:01, all of them with high affinity, indicating that the vaccine could protect both, the susceptible and the resistant populations.

Moreover, 21 of the CD8^+^ epitopes identified in our study are completely novel, while the other 26 were previously confirmed in immunological assays, however, they were not predicted as conserved sequences^34^. On the other hand, the fact that more than half of the sequences predicted in our investigation has been previously confirmed as Class I restricted epitopes by in-vitro experiments is also encouraging in the sense of validating our approach. Supporting this, 8 of the previously described epitopes of our list are localized among the 12.8% most dominant of the 1,051 epitopes described by Grifoni et al.^34^. Additionally, seven of the described epitopes and only one of the novel group were weak binders to the most frequent alleles, suggesting that the novel group concentrates higher affinity-binding CD8^+^ epitopes. We excluded these 8 weak binders from our selection and ended with 39 epitope candidates, 20 of them novel and 19, described before.

However, it is important to highlight that the description of an epitope as dominant by Grifoni et al.^34^ was not done based on the analysis of multiple peptides together. Since this vaccine formulation consists of presenting them all at the same time, the immunodominance may not be the same as the one reported^34^.

After the submission of this article, Kaderi Kibria et al.^29^ predicted epitopes for Class I and Class II of the M, S and E proteins of the Wuhan-Hu-1 reference strain only, and further checked the conservancy of these epitopes, disregarding the variants, among: (1) 180 SARS-CoV-2 genome sequences of GISAID; (2) 4390 new variants of SARS-CoV-2 and in (3) the sequences of 1000 spike, 245 M and 81 E proteins of SARS and MERS. They declared that they assessed the conservancy of the Omicron variant by observing the amino acid changes reported in the journals and if the amino acid changes were not located in the specific vaccine region they evaluated as conserved. However, the authors did not disclose any accession number for the Omicron genomes, sourcing information from reliable data bank, nor references of the consulted journals^29^. This is a very indirect and inappropriate method to assess conservancy of epitopes. The sequence of all variants, from Alpha to Omicron, were not aligned as they were in our investigation. They report a list of 10 Class II epitopes of M, E and S proteins. None of them is present in our list of Class II epitopes, which mostly concentrates sequences of ORF 1ab protein, and only contains two epitopes of E, one of M and none of S proteins. Among the 9 Class I epitopes of their list^29^, only the epitope YVYSRVKNL is present in our list. However, as previously stated, that epitope was also previously reviewed as biologically tested by Grifoni et al.^34^.

The number of deaths due to COVID-19 was correlated to the Class I HLA-A*01 phenotype^57^ and to the infected cases per million. HLA-A*01 is more frequent in Europe and America, where COVID-19 was more lethal and less frequent in East Asia, where severity was lower^57^. In our study, 27 CD8^+^ epitopes bind to HLA-A*01:01, a highly frequent allele. Furthermore, HLA-A*11 and HLA DQB1*04 were associated to mortality in Spain^58^. In our study, 14 Class I epitopes bind to HLA-A*11:01, and 10 of the 16 Class II epitopes bind to DQB1*04:02, suggesting that our vaccine could protect against mortality, severity and rate of infections.

On the other hand, we identified that, less epitopes bind to the ten most frequent HLA-Class I alleles in the World population (HLA-A*02:01, HLA-A*24:02, HLA-A*01:01, HLA-A*03:01, HLA-A*07:02, HLA-A*11:01, HLA-A*08:01, HLA-A*40:01, HLA-A*44:02, HLA-A* 44:03)^32^, while more epitopes tend to bind to the less frequent alleles. However, the novel epitope LMDGSYYQF binds strongly to HLA-A* 02:01, HLA-A*24:02 and HLA-A*01:01 alleles, and 7, 12 and 19 epitopes bind strongly to each one of these alleles, respectively. KVNSTLEQY, VVYRGTTTY and KLFDRYFKY epitopes are also strong binders of HLA-A* 01:01, HLA-A* 03:01 and HLA-A* 11:01 alleles. It might be adaptive for the virus to have conserved epitopes that do not bind to the Class I most frequent alleles, so they do not generate the antiviral CD8^+^ cytotoxic response. In this way, the SARS-CoV-2 virus could escape from the immune response in the majority of the World population, and the pandemic could continue. Supporting our hypothesis, the less frequent alleles HLA-A*02:02, HLA-B*15:03, and HLA-C*12:03 were the top presenters of conserved SARS-CoV-2 peptides^59^. On the other hand, the most frequent HLA-A*02:01 allele was associated to increased risk for COVID-19 and showed a lower capacity to present SARS-CoV-2 antigens compared to the less frequent alleles^60^. In our prediction, 9 epitopes are strong binders of the HLA-A*02:01 suggesting that they can generate good protection in susceptible subjects^60^. In addition, the HLA-A*11:01 and B*51:01 alleles also correlate with the extreme severity^61^. Twelve epitopes of our study are strong binders of HLA-A*11:01 and 3 are strong binders of B*51:01 indicating that the CD8^+^ epitopes of our vaccine might generate protection in the more susceptible HLA-phenotypes. Future in vivo studies could confirm this hypothesis.

It might be argued that that all the alleles shouldn’t be treated equally for an optimal vaccine design because some alleles are more abundant than others. The equation used in this study counts the number of alleles bound regardless of the allele frequency. This ends giving the same weight to high and low abundant alleles. Although it might have been useful for understanding the biology of the virus, this decision might have impact on the selection of the best epitopes for a massive vaccine.

The efficiency of our prediction was however, confirmed by the World population analysis, which disclosed high Class I and II epitope coverages, with high average hits and Pc 90^C^ values. The panels not only showed wide coverages but it also offered deep coverages, meaning that not only the individual is covered, but also that he is covered but multiple different epitopes. This is important when an epitope dominant for one specific allele suffers mutations that determine the pathogen immune escape. If the coverage is deep, the immune escape is less probable since it is unlikely that several epitopes that link to one specific allele would suffer simultaneously mutations. Different from other investigations^20,22,42^, in which population coverage was calculated using only selected alleles, in our study we used the IEDB full set of 27 alleles.

A map of epitopes recognized by CD4^+^ and CD8 T cell human responses against the whole SARS-CoV-2 proteome of individuals infected with the Alpha, Beta and Gamma variants was reported using a cytokine analysis^44^. Furthermore, the T cell responses of individuals vaccinated against the S or the M proteins of the Wuhan-Hu1 strain, cross recognize the SARS-CoV2 variants from Alpha to Omicron^62^. However, these epitopes have not yet been explored in vaccine formulations.

A criticism has been raised on the IEDB population coverage tool, which comes directly from the Allele Frequencies Net Database. This calculation was considered not representative of worldwide populations, and mainly influenced by the data of USA and Europe, which comprises the largest source of information in this database, while it probably misses a large amount of information of South America^50^. However, the two methodologies seem to be very different and hard to compare. The population data of IEDB is taken from a publically available repository that collects data from the International Histocompatibility Working Group (IHWG) certified labs from around the world, and its results can indicate an approximation since there is no other database with a more comprehensive data collection^21,22,25–28,38,42,63^.

In contrast, the work of Requena et al.^50^ was based on data of articles available at PubMed and datasets of AFNSD. These datasets were integrated by country calculating weighted allele frequencies. The authors claim to have included in this way, the frequencies of 91 alleles that were previously considered to have frequencies lower than 5% in South American Countries^50^. Different from the IEDB full list of HLA-A and HLA-B alleles used in our investigation^33^, the study by Requena et al. also included the HLA-C alleles^50^. However, in contrast to the HLA-Class II full reference list of IEDB which comprises HLA-DRB1*, HLA-DRB3*, HLA-DRB4* and HLA-DRB5*^63^, the study by Requena et al., only includes HLA-DRB1* alleles^50^.

The algorithm that we used in our study, filtered first the peptides that bound at least 14 of the 27 most frequent Class II alleles. From this subset, the epitopes with conserved identity in 100% of the 10 studied variants were further selected. For Class I prediction only epitopes with 100% of conservancy were selected which bound with percentile rank < 1% (weak binders) or with percentile rank < 0.5% (strong binders). We retrieved the protein FASTA sequences from the NCBI Genbank site, as recommended by the literature^20,21,23,24,26–28^ and confirmed the variant identity in the Pangolin site. In contrast, other investigations used multiple genomes instead of one genome for each variant^20,26,42,50^, however, none of them specified the proportional contribution of each variant to the set, and only one of them described the contribution of each geographic region to the whole set of genomes^50^.

Furthermore, we compared the prediction obtained in IEDB with the MHCFlurry method, which is a more recent tool^64^. In fact, different from IEDB, MHCFlurry is a Class-I only predictor. It is not possible to make Class-II predictions with it. Another issue is that MHCFlurry does not output scores for all alleles in a prediction, only for the best one. Because of this, it is not possible to calculate promiscuity scores, on which our final_score depends. As such, there is no direct way of making a comparison between our approach and MHCFlurry. This is yet another reason why we chose the IEDB tools for this study and likely why these are the most widely cited and used tools in this area of research. We however ran a prediction on our final selection of 47 Class I to verify whether the MHCflurry scores (named “presentation_score” in their algorithm) correlates to our final score. A Spearman correlation test confirmed that both scores have a positive correlation of R = 0.35 with a significant p value = 0.016. We noted however that our selection of epitopes includes information on all 27 alleles and the promiscuity, whereas the presentation score from MHCFlurry seems to only consider the strongest binding allele alone. We however noted that when comparing the best predicted allele in our final selection, the method we used agrees with MHCFlurry in a vast majority of cases. Namely, out of the 47 epitopes in the final selection, in 24 cases (51% of the total) the strongest binding allele predicted by MHCFlurry is the same predicted by us: (AMDEFIERY, ATSRTLSYY, GVYSVIYLY, HVISTSHKL, IVSTIQRKY, KAYNVTQAF, KMKDLSPRW, KQFDTYNLW, KVNSTLEQY, LVSDIDITF, NVIPTITQM, QVVDMSMTY, RLYYDSMSY, RTAPHGHVM, SFYEDFLEY, SSVELKHFF, STNVTIATY, STQDLFLPF, TILDGISQY, TLKEILVTY, VARDLSLQF, VYDPLQPEL, YLFDESGEF, YLITPVHVM). In 18 cases (38% of the total) the strongest binding allele predicted by MHCFlurry is the second strongest predicted by us: (AQLPAPRTL, EYADVFHLY, HADQLTPTW, HLDGEVITF, KLFDRYFKY, KSHKPPISF, LVKPSFYVY, QTFSVLACY, RTIKVFTTV, SLDNVLSTF, SSLPSYAAF, TSNQVAVLY, TTLPVNVAF, TTNGDFLHF, VMYMGTLSY, VVIPDYNTY, VVYRGTTTY, YVYSRVKNL). In 3 cases (6% of the total) the strongest binding allele predicted by MHCFlurry is the third strongest predicted by us: (LMDGSIIQF, QSAPHGVVF, TIKPVTYKL). In 1 case (2% of the total) the strongest binding allele predicted by MHCFlurry is the fourth strongest predicted by us: (QALLKTVQF). In 1 case (2% of the total) the strongest binding allele predicted by MHCFlurry is the sixth strongest predicted by us: (VAMPNLYKM). In no cases the difference in predictions was larger than this. These results show the strength of our methodology compared to the MHCFlurry tool^64^.

Moreover, also for comparison, we ran a prediction on the resumed selected 16 Class II epitopes using MixMHCPred2.0^65^ and the same 27 alleles that we used for prediction with IEDB recommended method. We observed that in 6 out of 16 peptides (38%) the strongest binding allele predicted by us (IEDB lowest percentile rank) is the same predicted by MIXMHCPred2.0 (% Rank Best Allele) for the exact first position (VTLVFLFVAAIFYLITPVHVM, LMIERFVSLAIDAYPLT, IILASFSASTSAFVET, QLIKVTLVFLFVAAIFYL, FLCLFLLPSLATVAY, FLAFVVFLLVTLAILTAL). In 3 more cases (19%) the strongest binding allele predicted by us is the second strongest predicted by MixMHCPred2.0 (MPNMLRIMASLVLARKHT, HLVDFQVTIAEILLI, SLFFFLYENAFLPFAM). In 1 case (7%) the strongest binding allele predicted by us is the third strongest predicted by MixMHCPred2.0 (CTERLKLFAAETLKA). In 2 cases (13%) the strongest binding allele predicted by us is the fourth strongest predicted by MixMHCPred2.0.2 (LFTRFFYVLGLAAIMQLFF, LMWLSYFIASFRLFARTR). In 1 case (7%) the strongest binding allele predicted by us is the fifth strongest predicted by MixMHCPred2.0 (FAWWTAFVTNVNASS) and in 1 case (17%) the strongest binding allele predicted by us is the sixth strongest predicted by MixMHCPred2.0 (VFLLVTLAILTALRLCAY). Both for IEDB and MixMHCpred2.0 tools give lower PR% or %Ranks, respectively, for epitopes having higher affinity binding to the HLA-allele receptor molecules. The mean ± SD of the PR % values attributed by IEDB to the best alleles (3.03% ± 3.6%) was not different from the mean of % Ranks attributed by MixMHCPred2.0 to the best alleles (3.66% ± 4.99%). However, taking into account the predictions of all the 27 alleles, we observed that the IEDB tool attributed low percentile ranks to many epitope-allele combinations, suggesting that it retrieved high affinity-binding epitopes to multiple alleles (mean ± SD = 7.08 ± 5.47%). In contrast, MixMHCpred2.0 attributed low % Rank to only a few combinations, and high % Rank, to the majority of the epitope-allele combinations (38.87% ± 4.38% Rank). The difference in variability of the ranks suggest differences in the algorithms of the two tools.

In addition, we also ran a prediction for the resumed selected 16 Class II epitopes using the NETMHCPan-4.0EL software (https://services.healthtech.dtu.dk/service.php?NetMHCIIpan-4.0). Different from the MHCFlurry and MixMHC Pred2.0 tools, which only give the % Rank values for the best alleles, both the IEDB and NETMHCPan-4.0EL tools give more detailed predictions and they both output scores for all alleles. We compared the Class II epitope predictions retrieved by IEDB and by NETMHCPan-4.0EL. NETMHCPan-4.0EL is an updated version^66^ that also uses the IEDB benchmark data and retrieves the % Rank EL value, which is the percentile rank of elution ligand prediction score for each epitope. We compared the performance of both methods for detection of the most powerful epitopes that bind with high affinity to MHC Class II DR, DQ and DP alleles. Only the epitopes binding to alleles with PR < 1% and PR < 5%, of the IEDB method, or with % Rank_EL < 1% (strong binders) and % Rank_EL < 5% (weak binders) of the NETMHCpan-4.0EL tool were considered for comparison. IEDB prediction detected as strong affinity-binders with PR < 1% values, 15 out of the 16 peptides (94%), while all the 16 sequences (100%) also bound with lower affinity (PR < 5%) to several alleles. Better than MIXMHCPred. 2.0, but less sensitive than IEDB, NETMHCpan-4.0EL disclosed 11 out of the 16 epitopes (69%) selected by IEDB (TERLKLFAAETLKATEE, VTLVFLFVAAIFYLITPVHVM, MPNMLRIMASLVLARKHT, LMWLSYFIASFRLFARTR, NSWLMWLIINLVQMAPISAM, LMIERFVSLAIDAYPLT, HLVDFQVTIAEILLI, IILASFSASTSAFVET, FAWWTAFVTNVNASS, CTERLKLFAAETLKA, FLAFVVFLLVTLAILTAL). Among these 11 epitopes, 5 bind to alleles with % Rank < 1% (46%), while all the 11 bind with % Rank < 5%, revealing that NETMHCPan-4.0EL is more strict or less sensitive than IEDB and detects less strong-binding epitopes. In fact, NETMHCpan-4.0EL agreed with IEDB in the identification of the same five epitopes among the strongest binders, with average % Rank < 1% values: IILASFSASTSAFVET (% R = 0.27%), TERLKLFAAETLKATEE (% R = 0.37%), MPNMLRIMASLVLARKHT (% R = 0.47%), CTERLKLFAAETLKA (% R = 0.49%), FAWWTAFVTNVNASS (% R = 0.83%). In addition to these 5 peptides, more 6 epitopes were also identified by NETMHCpan-4.0EL as weak binders to other alleles (average % Rank < 5% values): VTLVFLFVAAIFYLITPVHVM (% R = 2.65%), LMWLSYFIASFRLFARTR (% R = 2.82%), NSWLMWLIINLVQMAPISAM (% R = 4.81%), LMIERFVSLAIDAYPLT (% R = 1.84%), HLVDFQVTIAEILLI (% R = 3.70%), FLAFVVFLLVTLAILTAL (% R = 4.81%). Moreover, the promiscuity of the 11 epitopes disclosed by NETMHCPan-4.0EL was lower than that retrieved by IEDB. Only 2 peptides, which share the same core, bind to 19 out of 27 Class II alleles (TERLKLFAAETLKATEE, CTERLKLFAAETLKA), only one epitope to 10 alleles (MPNMLRIMASLVLARKHT), and the rest of them bind to only 1 to 5 alleles. In contrast, IEDB detected in all the 16 epitopes promiscuity values ranging from 4 to 14 alleles for PR < 5%, and from 1 to 4, for PR < 1%. We concluded that NETMHCPan-4.0EL predicted less strong and weak binders than IEDB, and predicted epitopes of lower promiscuity, being as such more strict or less sensitive than IEDB. However, IEDB and NETMHCPan-4.0EL agreed in the prediction of 69% of the Class II epitopes.

The designed multiepitope vaccine contains, at its N-terminal, the 39 CD8^+^ epitopes, joined by AAY spacers^21,27^, followed by 16 CD4^+^ epitopes, joined by GPGPG spacers^21,25,27,35^, with a 6 His-tag at its C-terminal. The epitopes are in decreasing order of final scores. Previous work^67,68^ indicate that the order of the epitopes in a multiepitope vaccine would impact their immunogenicity. This interesting question deserved further studies. A maximum number of conserved epitopes was preferred to ensure cross-protection, regardless size and hydrophobicity increases. The vaccine’s molecular weight is 90.84 kDa and it was non-toxic. The 220 kDa trimer of hemagglutinnin is industrially produced (Flubok^®^) and used as an alternative to the fractionated inactivated Influenza vaccine^69,70^. Proteins of less than 110 kDA were reported as good vaccines^71,72^ and a mild hydrophobicity was expected for a vaccine composed only of CD4^+^^73^ and CD8^+^ epitopes^74^. Its 93.24 aliphatic index indicated high termostability^75^. The vaccine is very stable and has a remarkable high half-life in mammalian, yeast and *E. coli* cells*.*

The multiepitope protein was planned to be expressed by *E. coli.* It has three N residues (asparagine), but since it lacks peptide signals, it might not be glycosylated in vivo even though it contains the potential motifs. Since *E. coli* does not perform glycosylation it seems that the presence of these N residues would not affect the protein expression. However, if other expression systems will be used for production and testing in the future, its final sequence might be changed from the current one in order to avoid glycosylation sites.

In COVID-19, activation of TLRs lead to activation of inflammasome, production of IL1β and IL-6. TLR3^−/−^, TLR4^−/−^ mice have increased susceptibility to SARS‐CoV^76^. The best tertiary structure model of the vaccine docked with the TLR4 receptor, indicating that the vaccine can efficiently induce the innate immune response. Long-lasting antibodies, B- and T-memory cells and a cytotoxic T cell peak were predicted to be generated during the adaptive immune response, despite the vaccine lack of B cell epitopes. A Th1, IL-2 and IFN-γ pro-inflammatory response and a mild TGF-β and IL-10 regulatory response were also detected, but IL-6, characteristic of the SARS inflammatory syndrom, was absent. The future vaccine formulation with QS21-containing adjuvants^77^ could strongly potentiate the vaccine antibody and T cell responses. We conclude that predictions of the immune responses generated by the multiepitope vaccine revealed encouraging results. However, in silico simulations are not enough to estimate the potential use of this formulation in the entire world population during this current pandemic. In vivo data about the immunogenicity of the proposed multiepitope vaccine is critical to evaluate the efficacy of this new strategy.

Finally, the evolution of the COVID-19 vaccine immune responses was monitored mainly considering the anti-S antibody titers. Now, 2 years and 6,469,458 deaths after, the need of vaccines based on conserved antigens and on T cell epitopes of SARS-CoV-2 virus has been recognized^17–20,26,27^.

## Methods

### Comparison of the sequences of SARS-CoV-2 coronavirus variants

To obtain the Fasta files of the genomes of the Wuhan-Hu-1 reference strain, Alpha, Beta, Gamma, Delta, Mu, Zeta, R.1, Lambda and Omicron variants, a search was performed using the NCBI Nucleotide database site (https://www.ncbi.nlm.nih.gov/nuccore). The genome reference codes used in this investigation were: NC_045512.2 for Wuhan-Hu1 of China, MW059036.1 for Alpha variant of England, MZ376663.1 for Beta variant of South Africa, MZ264787.1 for Gamma variant of Brazil, OK570404.1 for Delta variant of India, MZ710932.1 for Mu variant of Colombia, MZ833438.1 for Zeta variant of Peru, OK399319.1 for R.1 variant of California, OK546825.1 for Lambda variant of Peru and OK546825.1 for Omicron variant of South Africa (https://www.ncbi.nlm.nih.gov/nuccore). The lineage of each of the variants was further confirmed using the Pangolin website tool (https://pangolin.cog-uk.io/).

Furthermore, the ID numbers of the sequences of the S, M, N, E structural and ORF1ab, ORF3a, ORF 6, ORF7a, ORF7b, ORF8 and ORF10 non-structural proteins of each SARS-CoV-2 variant were obtained using the NCBI protein tool (http://www.ncbi.nlm.nih.gov/protein). Finally, the sequence of each protein of each variant was aligned with the sequence from the respective protein of the Wuhan-Hu1 reference strain using the Blast Alignment Search Tool, (https://blast.ncbi.nlm.nih.gov/Blast.cgi?PROGRAM=blastp&PAGE_TY=BlastSearch&_LOC=blasthome). Mutations and deletions were annotated.

### SARS-CoV-2 CD4^+^ T cell epitope prediction

Epitope prediction was performed on the following 11 proteins: S spike glycoprotein, E envelope protein, M membrane glycoprotein, N nucleocapsid protein, open reading frames ORF 1ab, ORF 3a, ORF 6, ORF 7a, ORF 7b, ORF 8 and ORF10, of each one of the following 10 SARS-CoV-2 virus strains: Wuhan-Hu-1, Alpha (B.1.1.7), Beta (B.1.351), Gamma or P1 (B.1.1.28.1), Delta (B.1.617.2), Omicron (B.1.1.529), Mµ (B.1.621), Zeta or P2 (B.1.1.28.2), Lambda (C.37 lineage) and R1 (B.1.427/B.1.429), using the Immune Epitope Database and Analysis Resource (IEDB) (https://www.iedb.org/home_v3.php).

Protein sequences of each SARS-CoV-2 protein of each strain used in this study are deposited in the NCBI Protein Repository Bank (https://www.ncbi.nlm.nih.gov/guide/proteins/). Table S14 shows the respective accession code numbers.

A CD4^+^ T cell epitope prediction was run, using 15-mer peptides and the Full HLA Class II reference list of the IEDB platform^78^, which contains 27 DR, DQ and DB alleles (Table S15A). The IEDB recommended method was used, which consists in using the Consensus approach combining the NN-align, SMM-align, CombLib and Sturniolo algorithms if any corresponding predictor is available for the molecule, otherwise NetMHCIIpan is used^34,44,45^**.**

Overall, predictions were carried out on 109 proteins since the sequence of ORF7a protein from the Zeta variant contains a “XX” sequence at amino acid positions 60–61, which is not recognized by the IEDB algorithm.

The sequences of the 109 proteins to the MHC-II Binding Predictions of the IEDB platform were submitted using a Python API (https://github.com/mattfemia/iedb-python). Peptides were selected only if they bound at least 14 of the 27 most frequent Class II alleles (promiscuity ≥ 14). From this subset, the epitopes with conserved identity in 100% of the 10 studied variants were further selected (corresponding to conservancy = 10).

All plots and visualizations were generated with the Seaborn and Matplotlib libraries.

In order to facilitate the selection and comparison of the best candidate peptides for a multiepitope conserved vaccine against SARS-CoV-2, it was necessary to define a quantitative metric corresponding to each peptide. Such a metric, here termed “final score” (*f*_*s*_), combines the values for promiscuity, conservancy and percentile rank into a single quantity.

As the selected CD4^+^ epitopes only include those with 100% conservancy, it would be redundant to add a conservancy-related term to *f*_*s*_. (It would be equivalent to adding 1 to the final score of all peptides).

Thus, in the case of CD4^+^ epitopes, the final score was calculated according to the equation:

$${f}_{s}=\frac{{N}_{bound} }{{N}_{total}}-\frac{{p}_{r}}{10}$$

where *N*_*bound*_ is the number of alleles bound by the epitope, *N*_*total*_ is the total number of HLA class II, and *p*_*r*_ is the percentile rank. As such, the final score is a combination of the relative promiscuity (number of alleles bound divided by the total number of alleles) and the percentile rank.

However, by design, the relative promiscuity will be a number between 0 and 1. The percentile rank, on the other hand, is a number between 0 and 100, inversely proportional to the quality of a given candidate peptide. That is to say, desirable peptides will have very low values of percentile rank (often around 1 or less). To reflect this in the calculation of *f*_*s*_, pr is multiplied by a negative coefficient (in this case − 0.1 or − 1/10). By adjusting this coefficient, the *p*_*r*_ influence over the final score can be increased or decreased accordingly.

As an illustrative example, a very bad candidate, with percentile rank of 100, would greatly decrease the value of *f*_*s*_, compared to a very good candidate with percentile rank 1.

The final score was used to identify of the best epitopes in the category plots discussed in the “Results” section.

Furthermore, for comparison, we also obtained a prediction of the 15-mer CD4^+^ T cell epitopes with overlapping of 10 amino acids^34,44,45^.

### SARS-CoV-2 CD8 T cell epitope prediction

Similar to the CD4^+^ case, a prediction was ran in a Python API (https://github.com/mattfemia/iedb-python). In this case the Class I API was used for the prediction of 9-mer length peptides, using the Full HLA Class I reference list of the IEDB platform^33^, which contains 27 HLA Class I A and B alleles (Table S15B). The IEDB 2020.9 recommended method was used, which corresponds to NetMHCpan EL 4.1 algorithm^79^.

Only epitopes with 100% of conservancy were selected. We then calculated two separate promiscuity values: the promiscuity for weak binders with percentile rank < 1%, and the promiscuity for strong binders that bind with percentile rank < 0.5%. As such, we could select for the peptides that bind strongly to the most possible alleles while still bind weakly to the rest. This was done because CD8^+^ peptides had less candidates with high promiscuity values, compared to CD4^+^.

As such, the formula for *f*_*s*_ had to be changed accordingly for CD8^+^ epitopes, favoring strong binding peptides, as given by the equation:

$${f}_{s}={c}_{0}\left(\frac{{N}_{<0.5\%} }{{N}_{total}}\right)+{c}_{1}\left(\frac{{N}_{<1\%} }{{N}_{total}}\right)-{{c}_{2}*p}_{r1\%}$$

where *c*_*0*_*, c*_*1*_ and *c*_*2*_ are coefficients that can be adjusted give more or less weight to the strong binder promiscuity, weak binder promiscuity, or percentile rank. The percentile rank contribution has a negative sign for the same reasons as discussed in the CD4^+^ case. The coefficients used in this study were *c*_*0*_ = 1, *c*_*1*_ = 0.1, and *c*_*2*_ = 0.1.

The score does not distinguish a strong from a weak CD8 epitope. What distinguish them is its percentile rank. Therefore, one epitope can be a strong binder of some alleles and a weak binder of other simultaneously. Weak binders are those CD8 epitopes that bind to alleles with percentile rank < 1% while strong binders are CD8 epitopes that bind to an allele with percentile rank < 0.5%. The percentile rank measures the affinity to the Class I HLA molecule. The formula for calculation of the score involves both the percentile rank < 1% and the percentile rank < 0.5%. The lower the percentile rank, the higher its affinity.

The following example illustrates how two different epitopes are classified. The first binds to 11 alleles strongly and to 3 weakly. The second binds to 2 alleles strongly and to 12 weakly. As such, both epitopes bind to a total of 14 alleles. However, we wish to favor the epitopes with more strong binds. The formula defined on Line was conceived for this purpose. The first epitope will receive a higher final score than the second.

As such, the CD4^+^ and CD8^+^ epitopes were selected according to these respective final scores.

### Population coverage of epitopes

The world population coverage of the predicted epitopes was determined using the IEDB platform (http://tools.iedb.org/population/)^80^.

#### Conservancy analysis

To evaluate the amino acid sequence conservation of 16 CD4 and 39 CD8 SARS-CoV-2 epitope regions of the structural and non-structural proteins, the amino acid substitutions found in the GISAID^81^ metadata file from August 2022 was used. This metadata file includes ~ 10 million genomes records that have been pre-aligned to the Wuhan-Hu-1 reference genome and includes a summary of the amino acid substitutions of the full-proteome for each sequenced SARS-CoV-2 genome. In addition to the amino acid substitutions, each genome record in the metadata file is annotated with information about the nucleotide sequence length, the WHO clade, PANGO lineage, geographical location of sample collection, collection date, and indeterminant nucleotide content (N-content). The precomputed amino acid substitutions were used to compute the extent of sequence conservation within each individual WHO clade and across all high-quality SARS-CoV-2 genomes.

For quality control, records were removed from further analysis if: (i) the genome sequence was less than 29,400 nucleotides, (ii) the genome sequence had an N-content proportion greater than 1%, or (iii) the metadata record was missing a geographical region of isolation, collection date, or not annotated with a variant clade. Following this quality control and including data since the earliest collection date of 01 November 2019, 3,630,666 genomes remained for conservation analysis.

The protein start and end coordinates of each CD4 and CD8 epitope within each mature protein was determined using the Wuhan-Hu-1 reference coordinates based on annotations provided in the Bacterial-Viral Bioinformatics Resource Center (BV-BRC)^51^. The protein amino acid substitution position obtained from the GISAID metadata file was then compared to the protein start and end positions of each CD4 and CD8 epitope to determine if the substitution was contained within the epitope region. The number of sequence records in which each epitope was affected by an amino acid substitution was determined for the entire collection of high quality genomes and for 14 disjoint WHO clades: Alpha, Beta, Delta, Epsilon, Eta, Gamma, Iota, Kappa, Lambda, Mu, Omicron, Theta, Zeta, and GH/490R (no WHO name assigned, PANGO lineages B.1.640 + B.1.640.*). From this analysis we were able to compute a conservation ratio between 0 and 1 quantifying the conservation of each epitope. The conservation of epitope *k* among all genomes was calculated according to formula (1) and the conservation of epitope *k* among all genomes of each WHO clade was calculated according to formula (2).

1 $$Genome-wide \ conservation \ of \ epitope \ k = 1-\frac{Total \ \# \ of \ genomes \ not \ conserved \ at \ epitope \ k}{Total \ \# \ of \ genomes}$$

2 $$Variant \ clade \ conservation \ of \ epitope \ k = 1-\frac{Total \ \# \ of \ clade \ genomes \ not \ conserved \ at \ epitope \ k}{Total \ \# \ of \ genomes \ in \ the \ clade}$$

### Prediction of physicochemical properties of the multiepitope vaccine

Physicochemical evaluation of the vaccine construct were carried out utilizing the Expasy portal’s ProtParam tool (https://web.expasy.org/protparam/)^21^, which predicts the following important parameters: theoretical isolectric point (pI), amino acid composition, molecular weight (MW), in vitro and in vivo half-life, instability and aliphatic index, and grand average of hydropathicity (GRAVY). Prediction of toxicity was addressed using PSI-BLAST BTXpred Server tool (https://webs.iiitd.edu.in/cgibin/btxpred/btx_main.pl).

### Secondary structure prediction

This was carried out using the PSIPRED tool (http://bioinf.cs.ucl.ac.uk/psipred/) which predicts the secondary structure, transmembrane topology, transmembrane helix, fold and domain recognition. The solvent accessibility of the vaccine was assessed using the RaptorX server (http://raptorx.uchicago.edu/), which is a protein structure and function prediction tool powered by deep learning.

### Tertiary structure modelling, refine and validation

Since the multiepitope vaccine includes epitopes of several different proteins of SARS-CoV-2, it was not possible to predict its 3D structure by homology. We used instead the Robetta Fold tool (https://robetta.bakerlab.org/submit.php)^82^ which performs accurate predictions of protein structures and interactions using a three-track neural network. Refinement of the models was performed with the Swiss Model (https://swissmodel.expasy.org/) and the PROCHEK tool of the PDBSUM (http://www.ebi.ac.uk/thornton-srv/databases/cgi-bin/pdbsum/GetPage.pl?pdbcode=index.html). Validation of the models was assessed by their Ramachandran plots, their best Q mean Z score, the highest Qmean Disco Global, the highest GMQE, solvation value in the Swiss Model, and the highest G factor that gives an estimate of how unusual the model is in the PROCHEK-PDBSUM tool.

### Molecular docking and interactions between the multiepitope vaccine and the TLR4 human receptor

In order to describe the interaction of the vaccine with the Toll-like human receptors, the tertiary structure models of TLR4 (2Z63) were obtained from the Protein Data Bank. The docking of the best vaccine model and TLR4 were analyzed using the ClusPro 2.0 (https://cluspro.bu.edu/login.php?redir=/home.php). The Cluspro 2.0 model, showing the highest cluster sizes and lowest interaction energy scores was considered as the best docked model^83^. Furthermore, the interactions between the receptors and vaccine were further analyzed by the PDBSUM tool (http://www.ebi.ac.uk/thornton-srv/databases/cgi-bin/pdbsum/GetPage.pl?pdbcode=index.html).

### Codon optimization, N glycosylation sites and cloning

Codon optimization is a crucial tool to enhance protein expression. For this purpose, the Java codon adaptation index (JCAT) (http://www.jcat.de/)^84^ was used, and the multieptitope protein DNA with optimized codons was obtained. Then, the codon adaptation index (CAI) value and GC content were also obtained. Furthermore, *Escherichia coli* (strain K12) was selected as the target organism, and XhoI and NcoI as the restriction enzyme cleavage sites. SnapGene (version 6.0.2) (https://www.snapgene.com/) was used to integrate the adapted DNA sequence to pET-28b (+) vector, between the NcoI and XhoI restriction sites with no stop codon. This vector enables enhanced protein yield and purification due to its C-terminally 6× HIS-tag. For determination of the N-glycosylation sites in human protein we used the tool NetNGlyc-1.0 (https://services.healthtech.dtu.dk/service.php?NetNGlyc-1.0).

### Prediction of the immune stimulations response to the multiepitope vaccine

For this purpose we used the C-ImmSim software (https://kraken.iac.rm.cnr.it/C-IMMSIM/)^85,86^. It determines the pathogen epitopes, using existing prediction methods, and evaluates the molecular interactions of the immune complexes. It combines a mesoscopic stimulator of the immune response with machine learning techniques (Neural Networks) for predictions of MHC-peptide binding interactions, linear B-cell epitopes and protein–protein potential estimation^60^. A sequence of 46 Alanine residues was used as a negative control. The sequence of the Full = Probable citrate synthase, mitochondrial; Flags: Precursor UniProtKB/Swiss-Prot: A4H9H8.1 was used a positive control.

### Statistical analysis

Correlation analysis between percentile ranks and promiscuity values of CD4^+^ T cell epitopes was performed using the Spearman two-tailed test. Correlation analysis between number of epitopes and number of amino acid of each proteins was carried out using the Pearson two-tailed test (Graphpad Prism 6).

## Supplementary Information

Supplementary Information.

## Data Availability

All relevant data supporting the findings of this investigation are available in the manuscript and its Supplemental information. The corresponding protein sequences for each protein of each SARS-CoV-2 strain used in this study are available at the NCBI Protein Bank (https://www.ncbi.nlm.nih.gov/guide/proteins/) and their respective accession code numbers were summarized in Table S14. The genome reference codes used in this investigation are available at the NCBI Nucleotide Bank (https://www.ncbi.nlm.nih.gov/nuccore).
