# Supplementary material for: A novel vaccine based on SARS-CoV-2 CD4+ and CD8+ T cell conserved epitopes from variants Alpha to Omicron
Source: Sci Rep. 2022 Oct 6;12:16731. doi: 10.1038/s41598-022-21207-2 (PMC9537284; doi:10.1038/s41598-022-21207-2)
Supplement: Supplementary file 1 — Supplementary Information. [file 41598_2022_21207_MOESM1_ESM.pdf]

Figure S1. Total epitopes and epitopes normalized by the number of amino acids of each protein

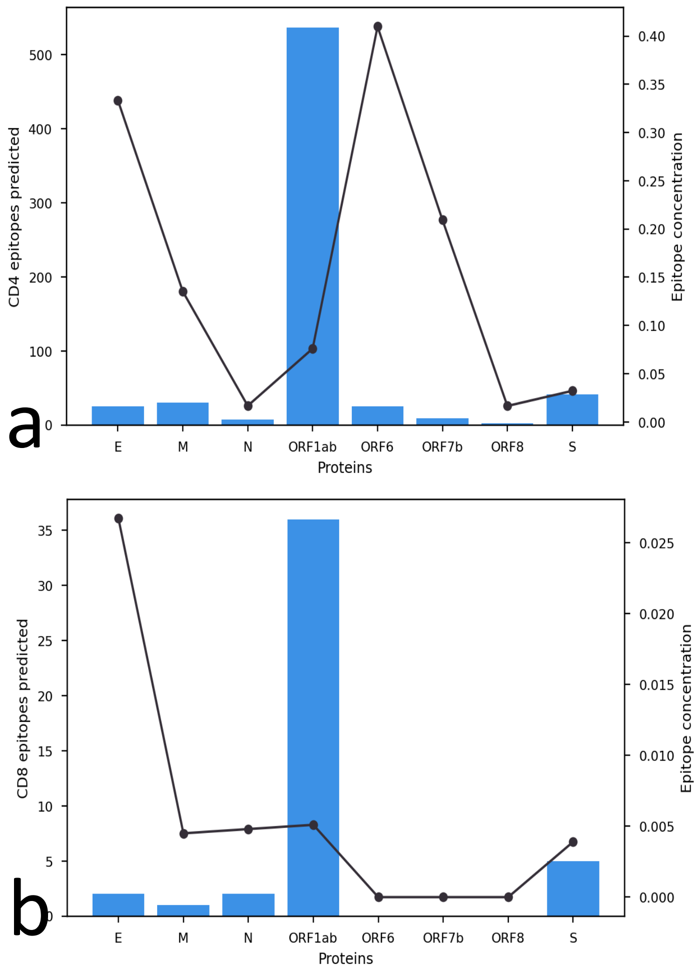

Figure S1: Number and density of conserved Class I and Class II predicted epitopes of the proteins of SARS-CoV-2 virus. The bars show the number of totally conserved epitopes predicted for each protein of the SARS-CoV-2 virus. The right-hand y axis shows the density of epitopes, which is the sum of all predicted epitopes divided by the number of amino acids of each protein. (A) $CD4^+$ conserved predicted epitopes. (B) $CD8^+$ conserved predicted epitopes

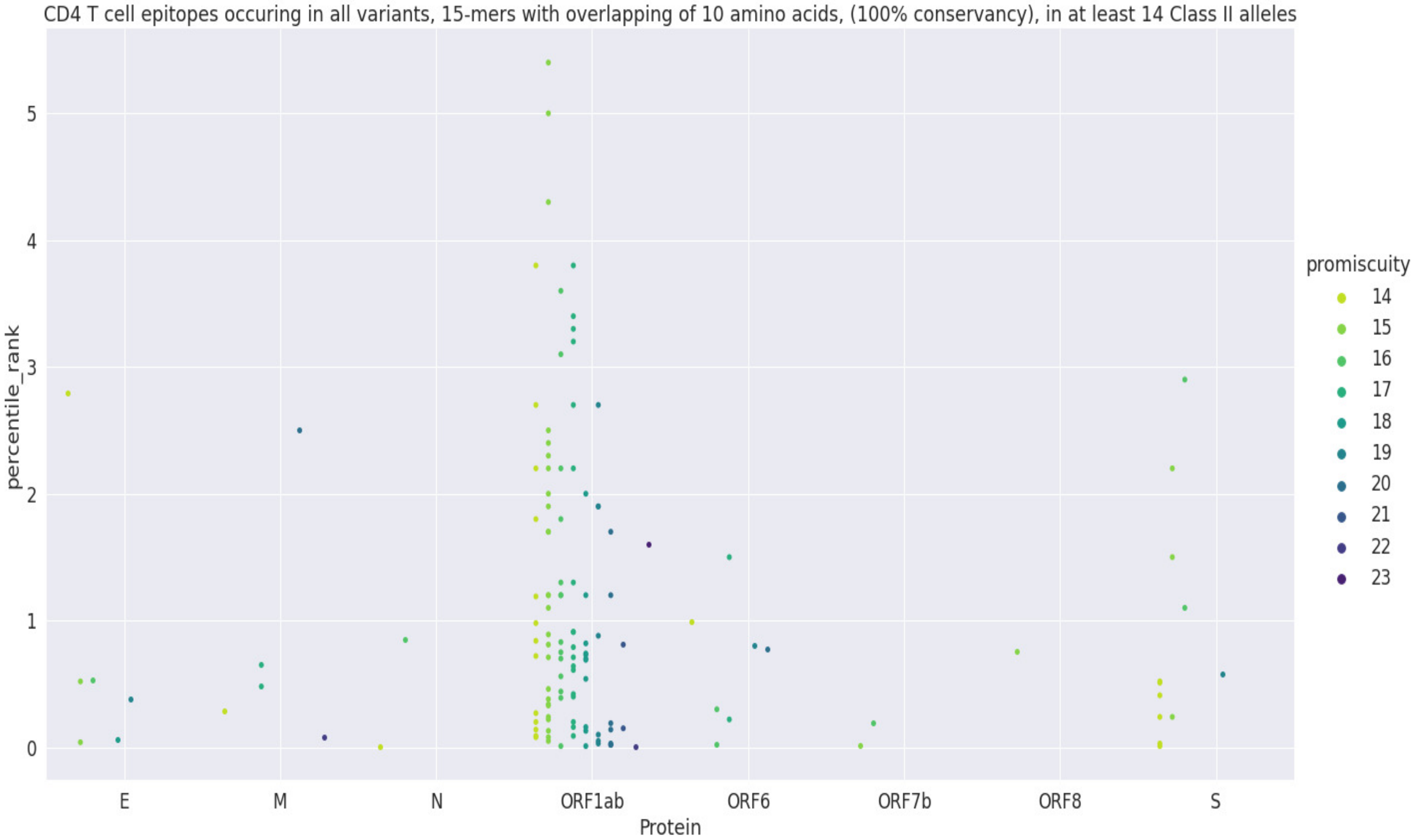

Figure S2: Prediction of CD4+ epitopes with 15 x 10 overlapping. The list of all 676 CD4+ T cell epitopes was additionally filtered for 15-mers epitopes with overlapping of 10 amino acids, spanning the whole protein sequences. This prediction retrieved 135 epitopes. This category plot shows the 15-mers with overlapping of 10 amino acids predicted CD4 T cell epitopes (100% conserved, promiscuity of at least 14). Each dot corresponds to an epitope, with the x-axis showing the respective protein where the epitope was found, and the y-axis showing the percentile rank. Within each x-axis protein, the color of the dots represents the promiscuity according to the color scale.

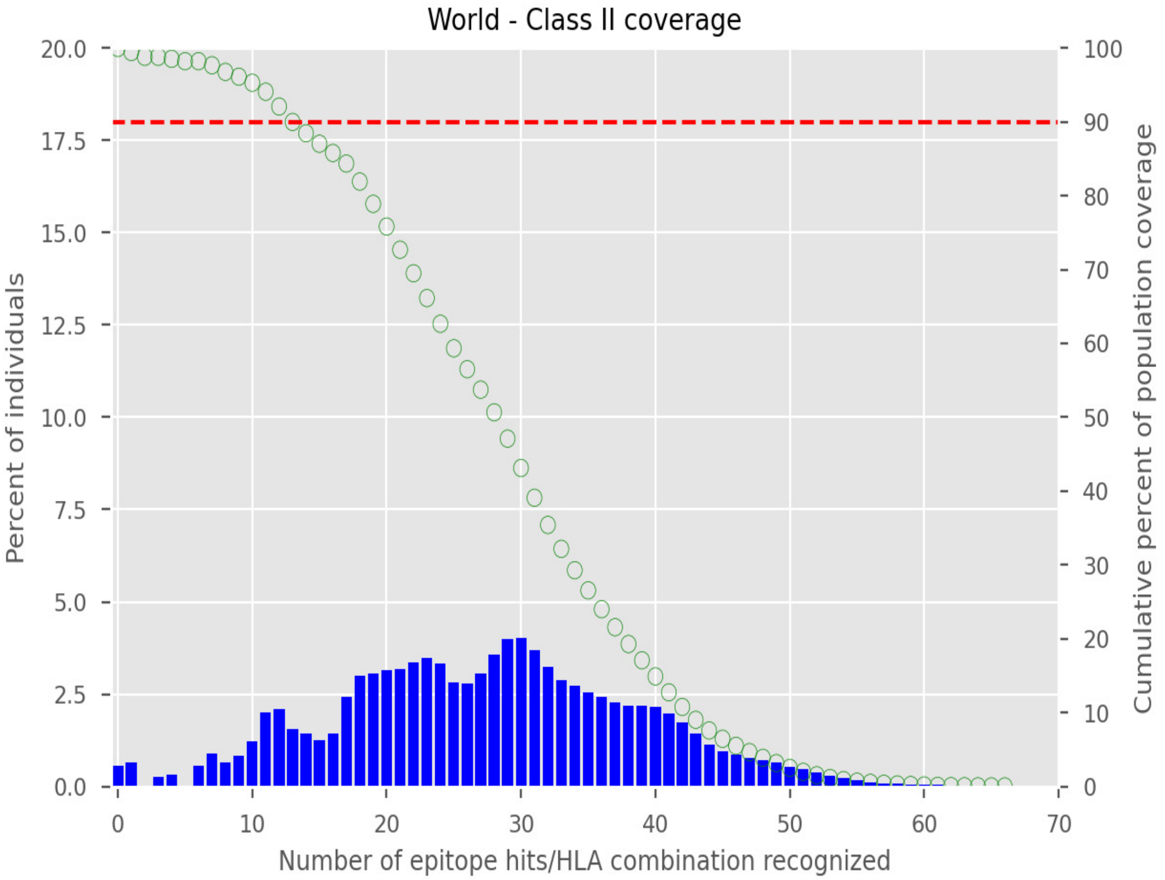

Class II PR < 10 % World coverage = 99.45 %
Average hit = 27.51 pc 90^{C} = 12.99

Figure S3: World population Class II coverage as predicted by the IEDB tool. The bar chart shows the number of epitope and allele combinations (x-axis) recognized by different fractions of the World population (x-axis). Here, about 4% of individuals will recognize 30 and 29 combinations, 3.6 % will recognize 28, about 3.5 % will recognize 23, etc. The right-hand y-axis, shows the cumulative percent of the World population coverage. Cumulatively, adding from right to left, at 90 % of individuals it is expected that at least 12.99 epitopes will be recognized. By the same way, about a little over 80 % of the population would recognize 19 or more, 60% about 25 or more, etc. The coverage values for the whole group of Class II epitopes is 99.45 %. The average number of epitope hits/ HLA combinations recognized by the population (average hit) is 27.51 and the minimum number of epitope hits/ HLA combinations recognized by the population (Pc 90^{C}) is 12.99.

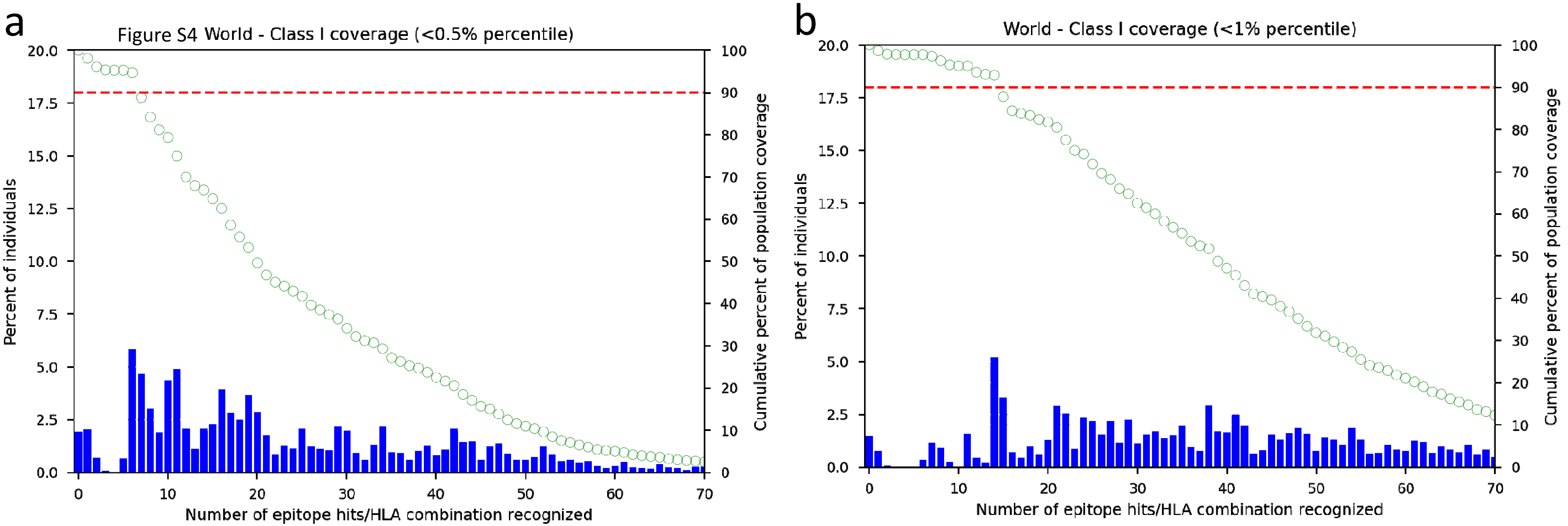

Class I PR < 0.5 % World coverage = 98.09 %
Average hit = 24.96
pc 90^{C} = 6.79

Class I PR < 1 % World coverage = 98.55 %
Average hit = 40.77
pc 90^{C} = 14.55

Figure S4: World population Class I coverage as predicted by the IEDB tool. The bar chart shows the number of combinations recognized by different fractions of the population. About 6% of individuals will recognize 6 combinations of strong binder epitopes and alleles, 4.91 % will recognize 11, 3.65 will recognize 19 and about 2.84% will recognize 20, etc. Cumulatively, adding from right to left, at 90% of individuals it is expected that at least 6.79 epitopes will be recognized. By the same way, about 75% of the population would recognize 11 or more, 50% about 21 or more etc. A little over 5% of individuals will recognize 14 combinations of weak binders epitopes and alleles, 3.26 % will recognize 15, about 3 % will recognize 38, etc. Cumulatively, adding from right to left, at 90% of individuals it is expected that at least 14.55 epitopes will be recognized. By the same way, about 75% of the population would recognize 25 or more, 50% about 38 or more, etc. Considering a percentile rank < 0.5 %, the population coverage for the strong binders was 98.09%, the average hit = 24.96% and the $pc90^{c}$ = 6.79. Furthermore, considering a percentile rank < 1 %, the population coverage for the weak binders was 98.55%, the average hit = 40.737% the $pc90^{c}$ = 14.55.

Secondary structure of the multiepitope protein

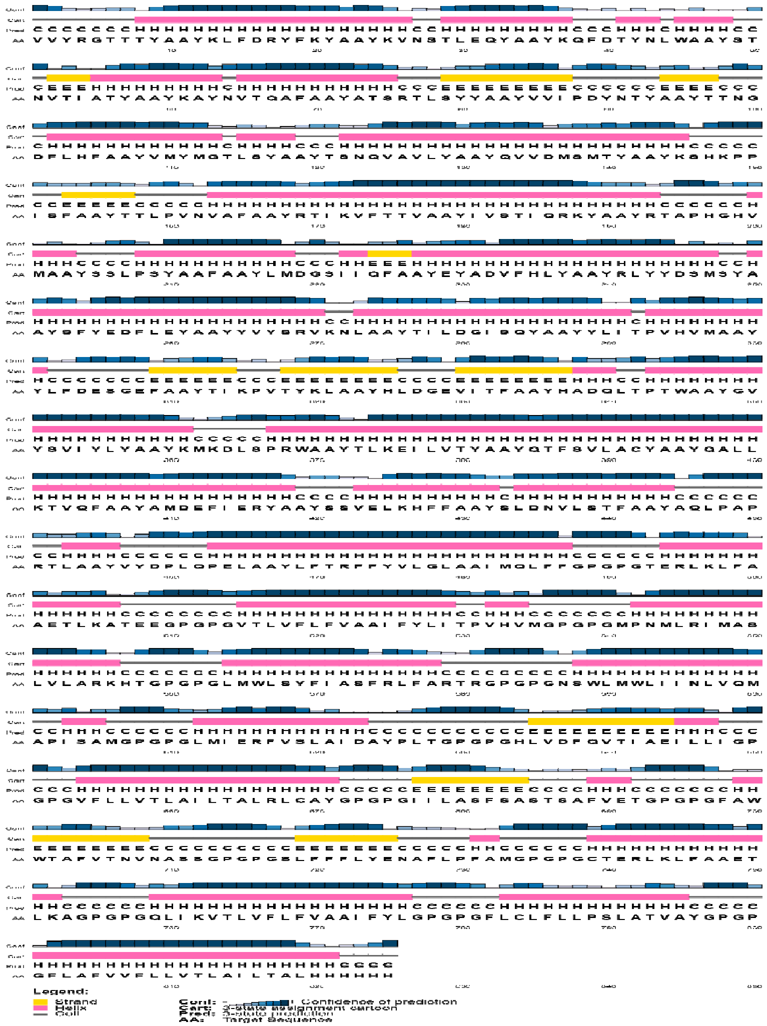

Figure S5: Secondary structure prediction of the multieptitope vaccine. The secondary structure prediction was performed using the PSIPRED tool. The strands are labeled in yellow, the Helixes in pink and the coils in green. Dark blue bars indicates highly confident prediction. 804/825 amino acids were predicted as to be extracellular and 18/825 as having membrane interactions.

Figure S6. Immune response. Negative control

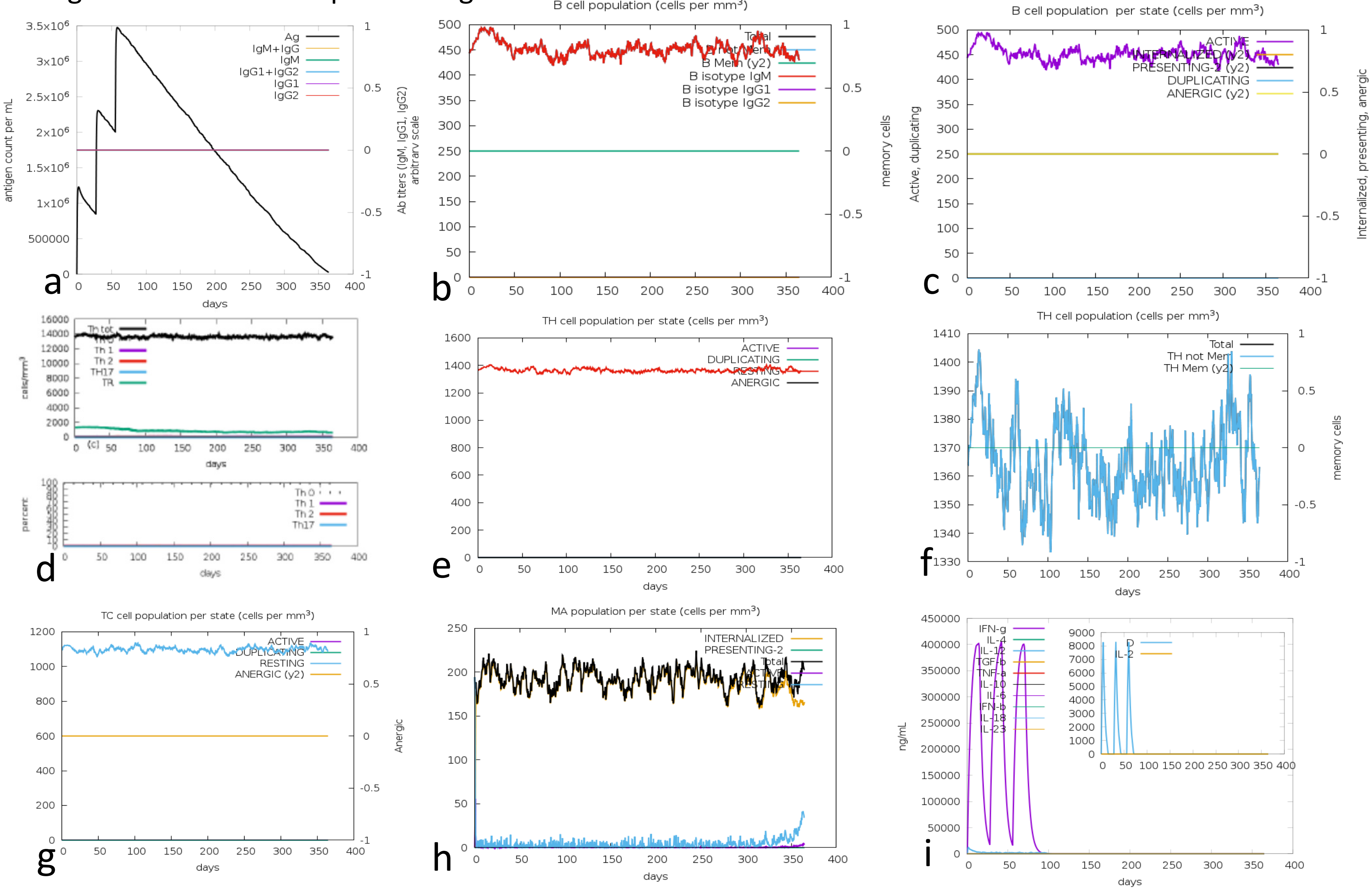

Figure S7. Immune response. Positive control

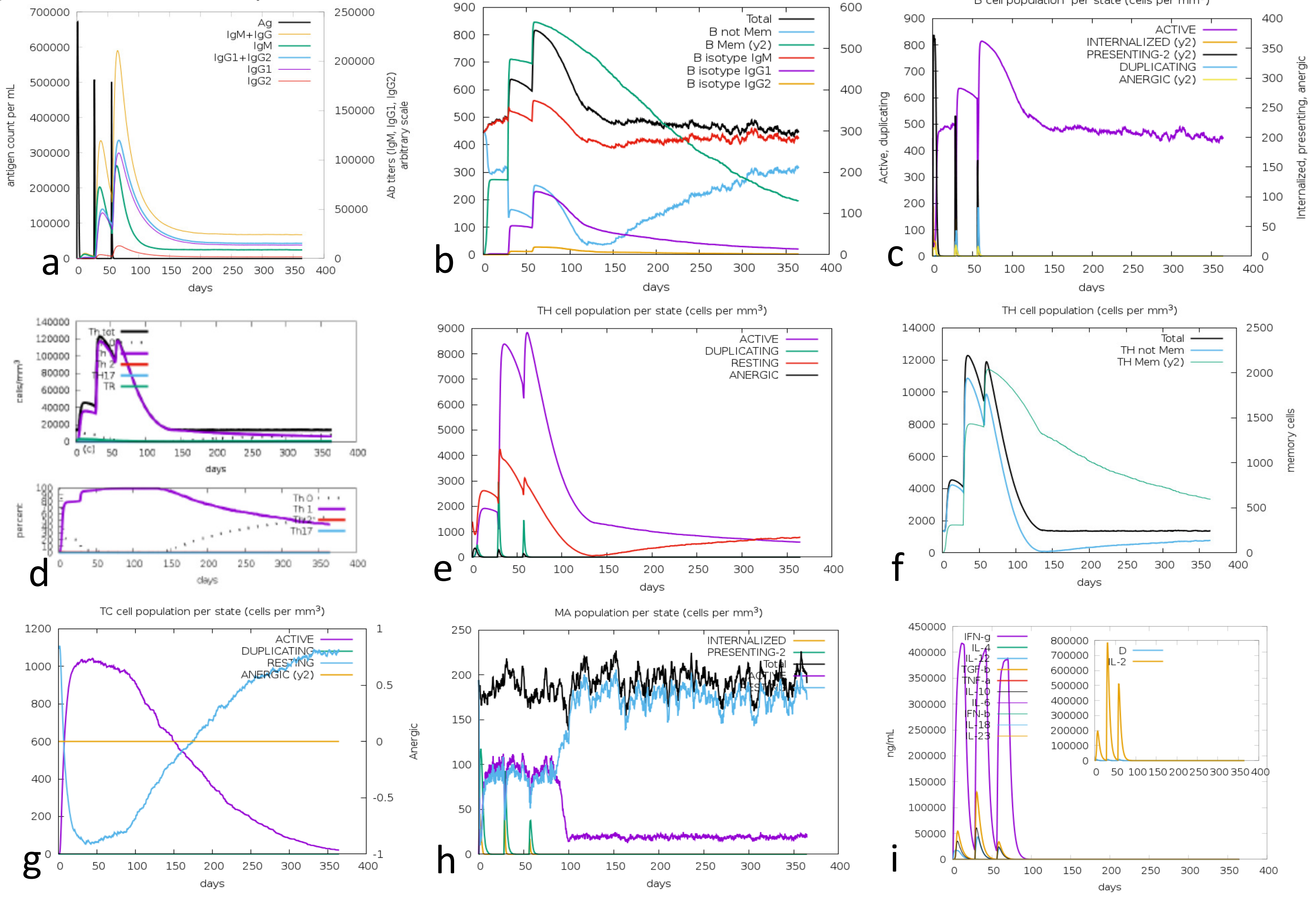

Table S1. List of mutatuions and deletion in comparison to the Wuhan-Hu1 reference virus

Protein	position	Ancestral (Wu)	Alfa (UK)	Beta (SA)	Gama (BR)	Delta (IN)	Mu (CO)	Zeta(PE)	Lambda (PE)	R1 (US)	Omicron (SA)
E	9	T									I
E	71	P		L							
M	3	D									G
M	19	Q									E
M	28	F								L	
M	63	A									T
M	82	I				T					
N	13	P							L		L
N	31	E									-
N	32	R									-
N	33	S									-
N	63	D				G					
N	80	P			R						
N	119	A						S			
N	187	S								L	
N	203	R			K	M		K	K	K	K
N	204	G			R			R	R	R	R
N	205	T		I			I				
N	213	G							C		
N	234	M						I			
N	349	Q				H					
N	366	T							I		
N	378	D				Y					
N	418	Q								H	
ORF10	13	I	M								
ORF1ab	110	H					Y				
ORF1ab	265	T		I							
ORF1ab	309	P				L					
ORF1ab	474	A					S				
ORF1ab	856	K									R
ORF1ab	882	T								I	
ORF1ab	1055	T					A				
ORF1ab	1056	V				L					
ORF1ab	1188	S			L						
ORF1ab	1246	T							I		
ORF1ab	1538	T					I				
ORF1ab	1640	P				L					
ORF1ab	1655	K		N							

ORF1ab	1795	K			Q						
ORF1ab	1921	P				Q					
ORF1ab	2083	S									-
ORF1ab	2084	L									I
ORF1ab	1924	S							G		
ORF1ab	2287	P							S		
ORF1ab	2387	F							V		
ORF1ab	2553	S			F						
ORF1ab	2710	A									T
ORF1ab	2980	D				N					
ORF1ab	3138	F				S					
ORF1ab	3201	L							P		
ORF1ab	3255	T							I		I
ORF1ab	3278	G							S		
ORF1ab	3353	K		R							
ORF1ab	3359	P			H						
ORF1ab	3395	P									H
ORF1ab	3468	L						V			
ORF1ab	3515	P		L							
ORF1ab	3580	H				Q					
ORF1ab	3674	L									-
ORF1ab	3675	S		-	T				-		-
ORF1ab	3676	G		-	S				-		-
ORF1ab	3677	F		-	L				-		
ORF1ab	3729	Q					R				
ORF1ab	3758	I									V
ORF1ab	3930	L						F			
ORF1ab	4616	P								S	
ORF1ab	4712	P								L	
ORF1ab	4715	P			L	L	L	L		L	L
ORF1ab	5215	G								C	
ORF1ab	5496	P					H				
ORF1ab	5665	E			D						
ORF1ab	5743	P					S				
ORF1ab	5763	G								R	
ORF1ab	5967	I									V
ORF1ab	6050	V					F				
ORF1ab	6075	I	T								
ORF1ab	6076	P				S					
ORF1ab	6337	P								H	

ORF1ab	6711	K				R					
ORF3a	26	S				L					
ORF3a	57	Q		H							
ORF3a	131	W		L							
ORF3a	171	S		L							
ORF3a	253	S			P						
ORF7a	3	I								T	
ORF7a	60	S						X			
ORF7a	61	T						X			
ORF7a	73	H						Q			
ORF7a	82	V			A	A					
ORF7a	93	V		F							
ORF7a	120	T				I					
ORF8	3	F						S			
ORF8	11	T					K				
ORF8	38	P					S				
ORF8	67	S					F				
ORF8	84	L	S								
ORF8	92	E			K						
ORF8	115	R		L							
S	18	L		F	F						
S	19	T				R	R				
S	21	T			N						
S	26	P			S						
S	67	A									V
S	69	H									-
S	70	V									-
S	74	G							V		
S	75	T							I		
S	80	D		A							
S	95	T					I				I
S	138	D			Y						
S	140	G									
S	142	G					D				D
S	143	V									-
S	144	Y									-
S	145	Y									-
S	152	W								L	
S	156	E					G				
S	157	F					-				

S	158	R					-				
S	190	R			S						
S	211	N									-
S	212	L									I
S	215	D		G							
S	215	-									E
S	216	-									P
S	217	-									E
S	222	A				V					
S	243	A		-							
S	244	L		-							
S	246	R							-		
S	247	S							-		
S	248	Y							-		
S	249	L							-		
S	250	T							-		
S	251	P							-		
S	252	G							-		
S	253	D							N		
S	339	G									D
S	371	S									L
S	373	S									P
S	375	S									F
S	417	K									N
S	440	N									K
S	446	G									S
S	452	L				R	R		Q		
S	474	S									
S	477	S									N
S	478	T				K	K				K
S	481	E									
S	484	E		K	K			K		K	A
S	490	F							S		
S	493	Q									R
S	496	G									S
S	498	Q									R
S	501	N		Y	Y						Y
S	505	Y									H
S	547	T									K
S	611	D									

S	614	D		G	G	G	G	G	G	G	G
S	655	H			Y						Y
S	677	Q		H							
S	679	N	-								K
S	680	S	-								
S	681	P	-			R	R				H
S	682	R	-								
S	683	R	-	W							
S	684	A	-								
S	685	R	-								
S	686	S	-								
S	687	V	I								
S	701	A		V							
S	764	N									K
S	769	G								V	
S	796	D									Y
S	856	N									K
S	859	T							N		
S	950	D				B	N				
S	954	Q									H
S	969	N									K
S	981	L									F
S	1027	T			I						
S	1083	H						N			
S	1176	V			F			F			
Mutations			4	20	26	25	22	15	19	17	48
Deletions			8	5	0	0	2	0	10	0	13
Mutation in S			1	9	11	7	9	4	7	4	33
S/total			25	45	42	28	41	27	37	24	69
Deletion in S			8	2	0	0	2	0	7	0	6
S/total			100	40	0	0	100	0	70	0	46
			alfa	beta	gamma	delta	um	zeta	lambda	R1	omicron

Table S2. Total $CD4^+$ conserved epitopes filtered using final scores in decreasing order

epitope	lenght	protein_clean	start	end	percentile_rank	promiscuity	final_score
TRFFYVLGLAAIMQL	15	ORF1ab	2335	2349	0.24	26	0.939
RFFYVLGLAAIMQLF	15	ORF1ab	2335	2349	0.56	26	0.907
ERLKLFAAETLKATE	15	ORF1ab	5452	5466	0.15	23	0.837
VTLVFLFVAAIFYLI	15	ORF1ab	2775	2789	0.19	23	0.833
FTRFFYVLGLAAIMQ	15	ORF1ab	2334	2348	0.24	23	0.828
FFYVLGLAAIMQLFF	15	ORF1ab	2337	2351	0.67	24	0.822
MPNMLRIMASLVLAR	15	ORF1ab	5018	5032	0.01	22	0.814
NMLRIMASLVLARKH	15	ORF1ab	5020	5034	0.01	22	0.814
PNMLRIMASLVLARK	15	ORF1ab	5016	5030	0.01	22	0.814
MWLSYFIASFRLFAR	15	M	91	105	0.08	22	0.807
LFVAAIFYLITPVHV	15	ORF1ab	2780	2794	0.55	23	0.797
TLVFLFVAAIFYLIT	15	ORF1ab	2777	2791	0.19	22	0.796
LFTRFFYVLGLAAIM	15	ORF1ab	2333	2347	0.38	22	0.777
LMWLSYFIASFRLFA	15	M	90	104	0.05	21	0.773
TERLKLFAAETLKAT	15	ORF1ab	5451	5465	0.15	21	0.763
WLSYFIASFRLFART	15	M	92	106	0.16	21	0.762
MLRIMASLVLARKHT	15	ORF1ab	5018	5032	0.2	21	0.758
RLKLFAAETLKATEE	15	ORF1ab	5453	5467	0.2	21	0.758
MWLIINLVQMAPISA	15	ORF1ab	2365	2379	0.6	22	0.755
FVAAIFYLITPVHVM	15	ORF1ab	2782	2796	0.63	22	0.752
MIERFVSLAIDAYPL	15	ORF1ab	5247	5261	0.65	22	0.75
HLVDFQVTIAEILLI	15	ORF6	3	17	0.67	22	0.748
VFLLVTLAILTALRL	15	E	25	39	0.38	21	0.74
IILASFSASTSAFVE	15	ORF1ab	475	489	0.01	20	0.74
FAWWTAFVTNVNASS	15	ORF1ab	6982	6996	0.01	20	0.74
ILASFSASTSAFVET	15	ORF1ab	476	490	0.02	20	0.739
LFFFLYENAFLPFAM	15	ORF1ab	3601	3615	0.02	20	0.739
SLFFFLYENAFLPFA	15	ORF1ab	3601	3615	0.03	20	0.738
CTERLKLFAAETLKA	15	ORF1ab	5450	5464	0.41	21	0.737
KVTLVFLFVAAIFYL	15	ORF1ab	2775	2789	0.04	20	0.737
FLCLFLLPSLATVAY	15	ORF1ab	3635	3649	0.05	20	0.736
FLAFVVFLLVTLAIL	15	E	20	34	0.06	20	0.735
KHFYWFFSNYLKRRV	15	ORF1ab	3151	3165	0.14	20	0.727
LVDFQVTIAEILLII	15	ORF6	4	18	0.54	21	0.724
HFYWFFSNYLKRRVV	15	ORF1ab	3152	3166	0.18	20	0.723
LVFLFVAAIFYLITP	15	ORF1ab	2778	2792	0.19	20	0.722
QTFFKLVNKFLALCA	15	ORF1ab	676	690	0.19	20	0.722
IIAMSAFAMMFVKHK	15	ORF1ab	3618	3632	0.58	21	0.72
QLIKVTLVFLFVAAI	15	ORF1ab	2772	2786	0.22	20	0.719
AAIFYLITPVHVMSK	15	ORF1ab	2784	2798	0.67	21	0.711
LRIMASLVLARKHTT	15	ORF1ab	5022	5036	0.32	20	0.709
LMPILTLTRALTAES	15	ORF1ab	4630	4644	0.37	20	0.704
LMIERFVSLAIDAYP	15	ORF1ab	5242	5256	0.75	21	0.703
LLVTLAILTALRLCA	15	E	27	41	0.38	20	0.703
LFLAFVVFLLVTLAI	15	E	19	33	0.03	19	0.701
LASFSASTSAFVETV	15	ORF1ab	477	491	0.03	19	0.701
LCLFLLPSLATVAYF	15	ORF1ab	3636	3650	0.03	19	0.701
ASFSASTSAFVETVK	15	ORF1ab	478	492	0.03	19	0.701
FFFLYENAFLPFAMG	15	ORF1ab	3602	3616	0.03	19	0.701
EFYAYLRKHFSMMIL	15	ORF1ab	5136	5150	0.05	19	0.699
WLIINLVQMAPISAM	15	ORF1ab	2366	2380	0.81	21	0.697

WSLFFFLYENAFLPF	15	ORF1ab	3600	3614	0.07	19	0.697
WLMWLIINLVQMAPI	15	ORF1ab	2363	2377	1.19	22	0.696
IERFVSLAIDAYPLT	15	ORF1ab	5248	5262	0.82	21	0.696
NSWLMWLIINLVQMA	15	ORF1ab	2361	2375	0.1	19	0.694
ILLIIMRTFKVSIWN	15	ORF6	14	28	0.1	19	0.694
EILLIIMRTFKVSIW	15	ORF6	13	27	0.1	19	0.694
MGIIAMSAFAMMFVK	15	ORF1ab	3616	3630	1.6	23	0.692
YAYLRKHFSMMILSD	15	ORF1ab	5135	5149	0.88	21	0.69
IKVTLVFLFVAAIFY	15	ORF1ab	2774	2788	0.53	20	0.688
SVQTFFKLVNKFLAL	15	ORF1ab	674	688	0.17	19	0.687
VQTFFKLVNKFLALC	15	ORF1ab	675	689	0.18	19	0.686
ETFKLSYGIATVREV	15	ORF1ab	5467	5481	0.56	20	0.685
FLFVAAIFYLITPVH	15	ORF1ab	2780	2794	0.19	19	0.685
VDFQVTIAEILLIIM	15	ORF6	5	19	0.57	20	0.684
FVVFLLVTLAILTAL	15	E	23	37	0.24	19	0.68
AFVVFLLVTLAILTA	15	E	22	36	0.25	19	0.679
KSAFYILPSIISNEK	15	ORF1ab	1350	1364	0.27	19	0.677
YSHVVAFNTLLFLMS	15	ORF1ab	3074	3088	1.4	22	0.675
VAAIFYLITPVHVMS	15	ORF1ab	2783	2797	0.67	20	0.674
LLIIMRTFKVSIWNL	15	ORF6	15	29	0.68	20	0.673
KVKYLYFIKGLNNLN	15	ORF1ab	4224	4238	0.31	19	0.673
LSYFIASFRLFARTR	15	M	93	107	0.35	19	0.669
TLMIERFVSLAIDAY	15	ORF1ab	5245	5259	1.1	21	0.668
GTLMIERFVSLAIDA	15	ORF1ab	5244	5258	0.74	20	0.667
LVTLAILTALRLCAY	15	E	28	42	0.38	19	0.666
FLLVTLAILTALRLC	15	E	26	40	0.38	19	0.666
SPFVMMSAPPAQYEL	15	ORF1ab	1802	1816	0.01	18	0.666
ESPFVMMSAPPAQYE	15	ORF1ab	1801	1815	0.01	18	0.666
QESPFVMMSAPPAQY	15	ORF1ab	1800	1814	0.01	18	0.666
SYFIASFRLFARTRS	15	M	94	108	0.39	19	0.665
LLFLAFVVFLLVTLA	15	E	18	32	0.02	18	0.665
VLLFLAFVVFLLVTL	15	E	17	31	0.02	18	0.665
CLFLLPSLATVAYFN	15	ORF1ab	3637	3651	0.03	18	0.664
LIIMRTFKVSIWNLD	15	ORF6	16	30	0.78	20	0.663
KCKSAFYILPSIISN	15	ORF1ab	1348	1362	0.42	19	0.662
KKCKSAFYILPSIIS	15	ORF1ab	1347	1361	0.42	19	0.662
GLMWLSYFIASFRLF	15	M	89	103	0.05	18	0.662
LAFVVFLLVTLAILT	15	E	21	35	0.06	18	0.661
FLAFLLFLVLIMLII	15	ORF7b	13	27	0.06	18	0.661
VGLMWLSYFIASFRL	15	M	88	102	0.07	18	0.66
FFLYENAFLPFAMGI	15	ORF1ab	3604	3618	0.07	18	0.66
PLIVTALRANSAVKL	15	ORF1ab	4125	4139	0.09	18	0.658
LMWLIINLVQMAPIS	15	ORF1ab	2364	2378	0.85	20	0.656
LFLLPSLATVAYFNM	15	ORF1ab	3638	3652	0.11	18	0.656
FHLVDFQVTIAEILL	15	ORF6	2	16	0.87	20	0.654
TFFKLVNKFLALCAD	15	ORF1ab	677	691	0.5	19	0.654
VLSFCAFAVDAAKAY	15	ORF1ab	4266	4280	0.13	18	0.654
YIWLGFIAGLIAIVM	15	S	1212	1226	0.51	19	0.653
IWLGFIAGLIAIVMV	15	S	1217	1231	0.51	19	0.653
LFFSYFAVHFISNSW	15	ORF1ab	2349	2363	0.52	19	0.652
HWLLLTILTSLLVLV	15	ORF1ab	3581	3595	0.16	18	0.651
TKHFYWFFSNYLKRR	15	ORF1ab	3150	3164	0.18	18	0.649
ESVQTFFKLVNKFLA	15	ORF1ab	673	687	0.19	18	0.648

WYIWLGFIAGLIAIV	15	S	1211	1225	0.58	19	0.646
FKLVNKFLALCADSI	15	ORF1ab	679	693	1.7	22	0.645
AMGIIAMSAFAMMFV	15	ORF1ab	3615	3629	1.7	22	0.645
AFLCLFLLPSLATVA	15	ORF1ab	3634	3648	0.24	18	0.643
VKYLYFIKGLNNLNR	15	ORF1ab	4225	4239	0.29	18	0.638
LGSLIYSTAALGVLM	15	ORF1ab	2240	2254	0.68	19	0.636
FKELLVYAADPAMHA	15	ORF1ab	4760	4774	0.31	18	0.636
WPLIVTALRANSAVK	15	ORF1ab	4120	4134	0.37	18	0.63
SAVVLLILMTARTVY	15	ORF1ab	3687	3701	0.38	18	0.629
AMPNMLRIMASLVLA	15	ORF1ab	5017	5031	0.01	17	0.629
NEFYAYLRKHFSMMI	15	ORF1ab	5132	5146	0.02	17	0.628
CFLAFLLFLVLIMLI	15	ORF7b	12	26	0.03	17	0.627
FAMGIIAMSAFAMMF	15	ORF1ab	3614	3628	1.9	22	0.625
LSVLQQLRVESSSKL	15	ORF1ab	3873	3887	0.42	18	0.625
CKSAFYILPSIISNE	15	ORF1ab	1349	1363	0.42	18	0.625
VSIWNLDYIINLIIK	15	ORF6	24	38	0.05	17	0.625
MFHLVDFQVTIAEIL	15	ORF6	1	15	0.8	19	0.624
SIWNLDYIINLIIKN	15	ORF6	25	39	0.43	18	0.624
YFIASFRLFARTRSM	15	M	95	109	0.44	18	0.623
FYAYLRKHFSMMILS	15	ORF1ab	5134	5148	0.44	18	0.623
SWLMWLIINLVQMAP	15	ORF1ab	2362	2376	0.45	18	0.622
PFAMGIIAMSAFAMM	15	ORF1ab	3612	3626	1.2	20	0.621
KDCVMYASAVVLLIL	15	ORF1ab	3676	3690	1.2	20	0.621
LIVTALRANSAVKLQ	15	ORF1ab	4126	4140	0.09	17	0.621
VVVLSFELLHAPATV	15	S	503	517	0.09	17	0.621
ILFTRFFYVLGLAAI	15	ORF1ab	2332	2346	0.11	17	0.619
AEILLIIMRTFKVSI	15	ORF6	12	26	0.11	17	0.619
AYLRKHFSMMILSDD	15	ORF1ab	5136	5150	0.88	19	0.616
VFLFVAAIFYLITPV	15	ORF1ab	2779	2793	0.14	17	0.616
QLFFSYFAVHFISNS	15	ORF1ab	2347	2361	0.15	17	0.615
FFSYFAVHFISNSWL	15	ORF1ab	2350	2364	0.53	18	0.614
DSYYSLLMPILTLTR	15	ORF1ab	4627	4641	0.16	17	0.614
VDSYYSLLMPILTLT	15	ORF1ab	4626	4640	0.16	17	0.614
GDYFVLTSHTVMPLS	15	ORF1ab	5546	5560	0.54	18	0.613
ARFYFYTSKTTVASL	15	ORF1ab	1420	1434	0.55	18	0.612
KLVNKFLALCADSII	15	ORF1ab	680	694	1.3	20	0.611
IINLVQMAPISAMVR	15	ORF1ab	2367	2381	0.56	18	0.611
WLLLTILTSLLVLVQ	15	ORF1ab	3582	3596	0.94	19	0.61
PKYKFVRIQPGQTFS	15	ORF1ab	3361	3375	0.2	17	0.61
LKLFAAETLKATEET	15	ORF1ab	5454	5468	0.96	19	0.608
IAEILLIIMRTFKVS	15	ORF6	11	25	0.22	17	0.608
GARFYFYTSKTTVAS	15	ORF1ab	1419	1433	0.6	18	0.607
RVVVLSFELLHAPAT	15	S	509	523	0.24	17	0.606
GARRVWTLMNVLTLV	15	ORF1ab	3704	3718	0.62	18	0.605
STKHFYWFFSNYLKR	15	ORF1ab	3149	3163	0.25	17	0.605
FFKLVNKFLALCADS	15	ORF1ab	678	692	1	19	0.604
RLYLDAYNMMISAGF	15	ORF1ab	6414	6428	0.63	18	0.604
VVFLLVTLAILTALR	15	E	24	38	0.28	17	0.602
FYWFFSNYLKRRVVF	15	ORF1ab	3153	3167	0.28	17	0.602
FSKLINIIIWFLLLS	15	ORF1ab	2223	2237	0.3	17	0.6
LIINLVQMAPISAMV	15	ORF1ab	2367	2381	0.68	18	0.599
TQLCQYLNTLTLAVP	15	ORF1ab	6846	6860	0.69	18	0.598
EETFKLSYGIATVRE	15	ORF1ab	5466	5480	0.7	18	0.597

PFVMMSAPPAQYELK	15	ORF1ab	1803	1817	0.33	17	0.597
KSAFVNLKQLPFFYY	15	ORF1ab	6358	6372	0.71	18	0.596
ASFNYLKSPNFSKLI	15	ORF1ab	2213	2227	0.34	17	0.596
YFAVHFISNSWLMWL	15	ORF1ab	2353	2367	0.34	17	0.596
PKVKYLYFIKGLNNL	15	ORF1ab	4223	4237	0.35	17	0.595
AYNMMISAGFSLWVY	15	ORF1ab	6419	6433	1.1	19	0.594
ARRVWTLMNVLTLVY	15	ORF1ab	3702	3716	1.1	19	0.594
EYSHVVAFNTLLFLM	15	ORF1ab	3073	3087	1.1	19	0.594
SPLYAFASEAARVVR	15	ORF1ab	531	545	0.73	18	0.594
RFYFYTSKTTVASLI	15	ORF1ab	1421	1435	0.74	18	0.593
MPILTLTRALTAESH	15	ORF1ab	4634	4648	0.38	17	0.592
RAMPNMLRIMASLVL	15	ORF1ab	5016	5030	0.01	16	0.592
TFKVSIWNLDYIINL	15	ORF6	21	35	0.02	16	0.591
GRFVLALLSDLQDLK	15	ORF1ab	4178	4192	0.77	18	0.59
AYYFMRFRRAFGEYS	15	ORF1ab	3061	3075	0.4	17	0.59
KGGRFVLALLSDLQD	15	ORF1ab	4173	4187	0.78	18	0.589
QWSLFFFLYENAFLP	15	ORF1ab	3599	3613	0.04	16	0.589
SAFYILPSIISNEKQ	15	ORF1ab	1351	1365	0.42	17	0.588
LIKVTLVFLFVAAIF	15	ORF1ab	2773	2787	0.42	17	0.588
MQLFFSYFAVHFISN	15	ORF1ab	2347	2361	0.05	16	0.588
SLLMPILTLTRALTA	15	ORF1ab	4631	4645	0.82	18	0.585
LAFLLFLVLIMLIIF	15	ORF7b	14	28	0.08	16	0.585
CQYLNTLTLAVPYNM	15	ORF1ab	6849	6863	1.2	19	0.584
IVTALRANSAVKLQN	15	ORF1ab	4124	4138	0.09	16	0.584
FIASFRLFARTRSMW	15	M	96	110	0.48	17	0.582
IASFRLFARTRSMWS	15	M	97	111	0.48	17	0.582
GIIAMSAFAMMFVKH	15	ORF1ab	3617	3631	1.6	20	0.581
SHVVAFNTLLFLMSF	15	ORF1ab	3075	3089	0.86	18	0.581
NRFLYIIKLIFLWLL	15	M	43	57	0.12	16	0.581
QKLLKSIAATRGATV	15	ORF1ab	4965	4979	0.12	16	0.581
FLAHIQWMVMFTPLV	15	ORF1ab	3122	3136	0.87	18	0.58
LAWPLIVTALRANSA	15	ORF1ab	4122	4136	0.88	18	0.579
DGARRVWTLMNVLTL	15	ORF1ab	3703	3717	0.88	18	0.579
AFLLFLVLIMLIIFW	15	ORF7b	15	29	0.14	16	0.579
VGDYFVLTSHTVMPL	15	ORF1ab	5545	5559	0.52	17	0.578
KLLKSIAATRGATVV	15	ORF1ab	4963	4977	0.15	16	0.578
LYAFASEAARVVRSI	15	ORF1ab	533	547	0.15	16	0.578
PLNIIPLTTAAKLMV	15	ORF1ab	4058	4072	0.53	17	0.577
SYYSLLMPILTLTRA	15	ORF1ab	4628	4642	0.16	16	0.577
VVDSYYSLLMPILTL	15	ORF1ab	4622	4636	0.16	16	0.577
AIFYLITPVHVMSKH	15	ORF1ab	2785	2799	0.91	18	0.576
AWPLIVTALRANSAV	15	ORF1ab	4123	4137	0.55	17	0.575
RIKIVQMLSDTLKNL	15	ORF1ab	6088	6102	0.55	17	0.575
ICLLQFAYANRNRFL	15	M	32	46	1.3	19	0.574
PLYAFASEAARVVRS	15	ORF1ab	532	546	0.56	17	0.574
FLLFLVLIMLIIFWF	15	ORF7b	16	30	0.19	16	0.574
GEYSHVVAFNTLLFL	15	ORF1ab	3071	3085	1.7	20	0.571
FYVLGLAAIMQLFFS	15	ORF1ab	2338	2352	1.7	20	0.571
FSYFAVHFISNSWLM	15	ORF1ab	2351	2365	0.61	17	0.569
ARYMRSLKVPATVSV	15	ORF1ab	1463	1477	0.24	16	0.569
PVVDSYYSLLMPILT	15	ORF1ab	4624	4638	0.24	16	0.569
FVFLVLLPLVSSQCV	15	S	2	16	0.24	16	0.569
LVGLMWLSYFIASFR	15	M	87	101	0.25	16	0.568

LRKHFSMMILSDDAV	15	ORF1ab	5141	5155	0.64	17	0.566
NRNRFLYIIKLIFLW	15	M	41	55	0.65	17	0.565
RNRFLYIIKLIFLWL	15	M	42	56	0.65	17	0.565
ANRNRFLYIIKLIFL	15	M	40	54	0.65	17	0.565
VMYASAVVLLILMTA	15	ORF1ab	3683	3697	1.4	19	0.564
KYLYFIKGLNNLNRG	15	ORF1ab	4223	4237	0.3	16	0.563
YIINLIIKNLSKSLT	15	ORF6	31	45	0.3	16	0.563
YLRKHFSMMILSDDA	15	ORF1ab	5137	5151	0.68	17	0.562
FLYENAFLPFAMGII	15	ORF1ab	3605	3619	0.32	16	0.561
DAYNMMISAGFSLWV	15	ORF1ab	6421	6435	0.71	17	0.559
LCTFLLNKEMYLKLR	15	ORF1ab	3180	3194	0.71	17	0.559
MTYRRLISMMGFKMN	15	ORF1ab	5974	5988	0.35	16	0.558
ERFVSLAIDAYPLTK	15	ORF1ab	5249	5263	1.1	18	0.557
AFNTLLFLMSFTVLC	15	ORF1ab	3078	3092	1.1	18	0.557
DGTLMIERFVSLAID	15	ORF1ab	5243	5257	1.1	18	0.557
INLVQMAPISAMVRM	15	ORF1ab	2369	2383	0.73	17	0.557
FKWDLTAFGLVAEWF	15	ORF1ab	2314	2328	0.36	16	0.557
FAVHFISNSWLMWLI	15	ORF1ab	2354	2368	0.37	16	0.556
VTLAILTALRLCAYC	15	E	29	43	0.38	16	0.555
VRIKIVQMLSDTLKN	15	ORF1ab	6087	6101	0.38	16	0.555
VVRIKIVQMLSDTLK	15	ORF1ab	6082	6096	0.38	16	0.555
TAFVTNVNASSSEAF	15	ORF1ab	6985	6999	0.01	15	0.555
NVVRIKIVQMLSDTL	15	ORF1ab	6082	6096	0.39	16	0.554
SYFAVHFISNSWLMW	15	ORF1ab	2351	2365	0.39	16	0.554
KVSIWNLDYIINLII	15	ORF6	23	37	0.02	15	0.554
LCFLAFLLFLVLIML	15	ORF7b	11	25	0.02	15	0.554
CLGSLIYSTAALGVL	15	ORF1ab	2238	2252	0.77	17	0.553
GGRFVLALLSDLQDL	15	ORF1ab	4173	4187	0.77	17	0.553
KESVQTFFKLVNKFL	15	ORF1ab	672	686	0.4	16	0.553
TEETFKLSYGIATVR	15	ORF1ab	5465	5479	0.78	17	0.552
ITQMNLKYAISAKNR	15	ORF1ab	4928	4942	0.41	16	0.552
SVLLFLAFVVFLLVT	15	E	16	30	0.04	15	0.552
NSVLLFLAFVVFLLV	15	E	15	29	0.04	15	0.552
TQWSLFFFLYENAFL	15	ORF1ab	3598	3612	0.04	15	0.552
FNTLLFLMSFTVLCL	15	ORF1ab	3080	3094	0.79	17	0.551
NTLLFLMSFTVLCLT	15	ORF1ab	3081	3095	0.79	17	0.551
FKLSYGIATVREVLS	15	ORF1ab	5469	5483	0.79	17	0.551
IMQLFFSYFAVHFIS	15	ORF1ab	2346	2360	0.05	15	0.551
ISMWALIISVTSNYS	15	ORF1ab	3731	3745	0.44	16	0.549
SRTLSYYKLGASQRV	15	M	173	187	0.07	15	0.549
PSFYVYSRVKNLNSS	15	E	54	68	0.82	17	0.548
SAFVNLKQLPFFYYS	15	ORF1ab	6359	6373	0.82	17	0.548
YILFTRFFYVLGLAA	15	ORF1ab	2331	2345	0.08	15	0.548
TEAFEKMVSLLSVLL	15	ORF1ab	3905	3919	1.2	18	0.547
QLCQYLNTLTLAVPY	15	ORF1ab	6847	6861	1.2	18	0.547
NGDFLHFLPRVFSAV	15	ORF1ab	2878	2892	1.2	18	0.547
DCVMYASAVVLLILM	15	ORF1ab	3681	3695	1.2	18	0.547
DYIINLIIKNLSKSL	15	ORF6	30	44	0.46	16	0.547
VTALRANSAVKLQNN	15	ORF1ab	4124	4138	0.09	15	0.547
VAFNTLLFLMSFTVL	15	ORF1ab	3077	3091	0.48	16	0.545
TQMNLKYAISAKNRA	15	ORF1ab	4929	4943	0.11	15	0.545
LYLTFYLTNDVSFLA	15	ORF1ab	3110	3124	0.87	17	0.543
KQLIKVTLVFLFVAA	15	ORF1ab	2771	2785	0.13	15	0.543

RFLYIIKLIFLWLLW	15	M	44	58	0.14	15	0.542
KFPLKLRGTAVMSLK	15	ORF1ab	7043	7057	0.14	15	0.542
TLIVNSVLLFLAFVV	15	E	11	25	0.53	16	0.54
LNIIPLTTAAKLMVV	15	ORF1ab	4059	4073	0.53	16	0.54
LLFLVLIMLIIFWFS	15	ORF7b	17	31	0.53	16	0.54
FHQKLLKSIAATRGA	15	ORF1ab	4963	4977	0.16	15	0.54
CQPILLLDQALVSDV	15	ORF1ab	2566	2580	0.91	17	0.539
NVGDYFVLTSHTVMP	15	ORF1ab	5541	5555	0.91	17	0.539
VAEWFLAYILFTRFF	15	ORF1ab	2324	2338	0.17	15	0.539
YTQLCQYLNTLTLAV	15	ORF1ab	6845	6859	0.93	17	0.537
LNKEMYLKLRSDVLL	15	ORF1ab	3185	3199	0.56	16	0.537
NKEMYLKLRSDVLLP	15	ORF1ab	3186	3200	0.56	16	0.537
YYFMRFRRAFGEYSH	15	ORF1ab	3062	3076	0.57	16	0.536
IAMSAFAMMFVKHKH	15	ORF1ab	3619	3633	0.58	16	0.535
ANEYRLYLDAYNMMI	15	ORF1ab	6413	6427	1.7	19	0.534
DYFVLTSHTVMPLSA	15	ORF1ab	5547	5561	0.96	17	0.534
TPKYKFVRIQPGQTF	15	ORF1ab	3361	3375	0.22	15	0.534
VVLKKLKKSLNVAKS	15	ORF1ab	3975	3989	0.6	16	0.533
VASIKNFKSVLYYQN	15	ORF1ab	5165	5179	0.6	16	0.533
AARYMRSLKVPATVS	15	ORF1ab	1462	1476	0.23	15	0.533
AALALLLLDRLNQLE	15	N	217	231	0.61	16	0.532
KYKFVRIQPGQTFSV	15	ORF1ab	3363	3377	0.61	16	0.532
ASAVVLLILMTARTV	15	ORF1ab	3686	3700	0.24	15	0.532
MFVFLVLLPLVSSQC	15	S	1	15	0.24	15	0.532
CLLQFAYANRNRFLY	15	M	33	47	0.99	17	0.531
GDFLHFLPRVFSAVG	15	ORF1ab	2879	2893	0.99	17	0.531
VVLLSVLQQLRVESS	15	ORF1ab	3867	3881	0.62	16	0.531
VPLNIIPLTTAAKLM	15	ORF1ab	4057	4071	0.25	15	0.531
LYLDAYNMMISAGFS	15	ORF1ab	6418	6432	0.65	16	0.528
YLYLTFYLTNDVSFL	15	ORF1ab	3109	3123	0.65	16	0.528
LPFAMGIIAMSAFAM	15	ORF1ab	3612	3626	1.4	18	0.527
LTFYLTNDVSFLAHI	15	ORF1ab	3112	3126	0.66	16	0.527
YLYFIKGLNNLNRGM	15	ORF1ab	4227	4241	0.29	15	0.527
NEYRLYLDAYNMMIS	15	ORF1ab	6414	6428	1.8	19	0.524
YLTFYLTNDVSFLAH	15	ORF1ab	3111	3125	0.7	16	0.523
EAARYMRSLKVPATV	15	ORF1ab	1461	1475	0.33	15	0.523
HSLSHFVNLDNLRAN	15	ORF1ab	2516	2530	0.34	15	0.522
NIIPLTTAAKLMVVI	15	ORF1ab	4057	4071	1.1	17	0.52
NLDYIINLIIKNLSK	15	ORF6	28	42	1.1	17	0.52
IIIWFLLLSVCLGSL	15	ORF1ab	2229	2243	0.73	16	0.52
VFHLYLQYIRKLHDE	15	ORF1ab	5269	5283	0.37	15	0.519
GSLIYSTAALGVLMS	15	ORF1ab	2241	2255	0.75	16	0.518
DVFHLYLQYIRKLHD	15	ORF1ab	5271	5285	0.38	15	0.518
AVVLLILMTARTVYD	15	ORF1ab	3688	3702	0.38	15	0.518
WPQIAQFAPSASAFF	15	N	301	315	0.01	14	0.518
PQIAQFAPSASAFFG	15	N	299	313	0.01	14	0.518
WWTAFVTNVNASSSE	15	ORF1ab	6984	6998	0.01	14	0.518
WFLAYILFTRFFYVL	15	ORF1ab	2327	2341	0.01	14	0.518
QSLLIVNNATNVVIK	15	S	115	129	0.01	14	0.518
TQSLLIVNNATNVVI	15	S	114	128	0.01	14	0.518
SLLIVNNATNVVIKV	15	S	116	130	0.01	14	0.518
YLCFLAFLLFLVLIM	15	ORF7b	10	24	0.02	14	0.517
NCYLATALLTLQQIE	15	ORF1ab	1673	1687	0.4	15	0.516

VVLSFELLHAPATVC	15	S	511	525	0.03	14	0.516
DMTYRRLISMMGFKM	15	ORF1ab	5973	5987	0.41	15	0.515
FGEYSHVVAFNTLLF	15	ORF1ab	3071	3085	1.9	19	0.514
NDMILSLLSKGRLII	15	ORF1ab	7066	7080	1.9	19	0.514
TLLFLMSFTVLCLTP	15	ORF1ab	3082	3096	0.79	16	0.514
LLFLMSFTVLCLTPV	15	ORF1ab	3083	3097	0.79	16	0.514
AAIMQLFFSYFAVHF	15	ORF1ab	2344	2358	0.05	14	0.514
VNEFYAYLRKHFSMM	15	ORF1ab	5134	5148	0.05	14	0.514
AIMQLFFSYFAVHFI	15	ORF1ab	2345	2359	0.05	14	0.514
WNTFTRLQSLENVAF	15	ORF1ab	6445	6459	0.81	16	0.512
LKRRVVFNGVSFSTF	15	ORF1ab	3160	3174	0.81	16	0.512
EVITFDNLKTLLSLR	15	ORF1ab	1549	1563	0.44	15	0.512
SFYVYSRVKNLNSSR	15	E	55	69	0.82	16	0.511
MNLKYAISAKNRART	15	ORF1ab	4931	4945	0.08	14	0.511
LCQYLNTLTLAVPYN	15	ORF1ab	6848	6862	1.2	17	0.51
NSTVLSFCAFAVDAA	15	ORF1ab	4263	4277	1.2	17	0.51
CVMYASAVVLLILMT	15	ORF1ab	3682	3696	1.2	17	0.51
WNLDYIINLIIKNLS	15	ORF6	27	41	1.2	17	0.51
QCFKMFYKGVITHDV	15	ORF1ab	5791	5805	0.83	16	0.51
TIAEILLIIMRTFKV	15	ORF6	10	24	0.83	16	0.51
EASFNYLKSPNFSKL	15	ORF1ab	2212	2226	0.46	15	0.51
GLVASIKNFKSVLYY	15	ORF1ab	5166	5180	0.46	15	0.51
QGLVASIKNFKSVLY	15	ORF1ab	5165	5179	0.46	15	0.51
LINIIIWFLLLSVCL	15	ORF1ab	2226	2240	0.09	14	0.51
IYLYLTFYLTNDVSF	15	ORF1ab	3108	3122	0.47	15	0.509
VNSVLLFLAFVVFLL	15	E	14	28	0.1	14	0.509
QQESPFVMMSAPPAQ	15	ORF1ab	1799	1813	0.1	14	0.509
DAALALLLLDRLNQL	15	N	216	230	0.85	16	0.508
ALCTFLLNKEMYLKL	15	ORF1ab	3178	3192	0.85	16	0.508
LSPLYAFASEAARVV	15	ORF1ab	530	544	1.6	18	0.507
ASEFSSLPSYAAFAT	15	ORF1ab	3945	3959	0.12	14	0.507
LSFCAFAVDAAKAYK	15	ORF1ab	4267	4281	0.13	14	0.506
LKQLIKVTLVFLFVA	15	ORF1ab	2770	2784	0.13	14	0.506
TFKLSYGIATVREVL	15	ORF1ab	5468	5482	0.51	15	0.505
AEWFLAYILFTRFFY	15	ORF1ab	2325	2339	0.14	14	0.505
INIIIWFLLLSVCLG	15	ORF1ab	2226	2240	0.14	14	0.505
FYVYSRVKNLNSSRV	15	E	56	70	0.52	15	0.504
KDMTYRRLISMMGFK	15	ORF1ab	5972	5986	0.52	15	0.504
SEFSSLPSYAAFATA	15	ORF1ab	3943	3957	0.15	14	0.504
HQKLLKSIAATRGAT	15	ORF1ab	4964	4978	0.15	14	0.504
NWLKQLIKVTLVFLF	15	ORF1ab	2768	2782	0.15	14	0.504
FPLKLRGTAVMSLKE	15	ORF1ab	7048	7062	0.15	14	0.504
LMCQPILLLDQALVS	15	ORF1ab	2564	2578	0.91	16	0.502
LVAEWFLAYILFTRF	15	ORF1ab	2323	2337	0.18	14	0.501
KPSFYVYSRVKNLNS	15	E	53	67	1.3	17	0.5
LLMPILTLTRALTAE	15	ORF1ab	4632	4646	1.3	17	0.5
EAFEKMVSLLSVLLS	15	ORF1ab	3906	3920	1.3	17	0.5
SFNYLKSPNFSKLIN	15	ORF1ab	2214	2228	1.3	17	0.5
ALALLLLDRLNQLES	15	N	218	232	0.93	16	0.5
VSFLAHIQWMVMFTP	15	ORF1ab	3120	3134	0.94	16	0.499
SMWALIISVTSNYSG	15	ORF1ab	3732	3746	0.2	14	0.499
KELLVYAADPAMHAA	15	ORF1ab	4761	4775	0.2	14	0.499
SAFAMMFVKHKHAFL	15	ORF1ab	3622	3636	0.58	15	0.498

LVASIKNFKSVLYYQ	15	ORF1ab	5167	5181	0.58	15	0.498
FLYIIKLIFLWLLWP	15	M	45	59	0.22	14	0.497
RQFHQKLLKSIAATR	15	ORF1ab	4957	4971	0.97	16	0.496
YKFVRIQPGQTFSVL	15	ORF1ab	3364	3378	0.61	15	0.495
NIIIWFLLLSVCLGS	15	ORF1ab	2228	2242	0.24	14	0.495
QPYRVVVLSFELLHA	15	S	506	520	0.24	14	0.495
AVNLLTNMFTPLIQP	15	ORF1ab	3023	3037	1	16	0.493
DEMIAQYTSALLAGT	15	S	867	881	1	16	0.493
FCLEASFNYLKSPNF	15	ORF1ab	2208	2222	0.64	15	0.492
CVPLNIIPLTTAAKL	15	ORF1ab	4056	4070	0.27	14	0.492
WICLLQFAYANRNRF	15	M	31	45	2.5	20	0.491
YANRNRFLYIIKLIF	15	M	39	53	0.65	15	0.491
YVTQQLIRAAEIRAS	15	S	1007	1021	0.65	15	0.491
VVAFNTLLFLMSFTV	15	ORF1ab	3077	3091	0.66	15	0.49
CLVGLMWLSYFIASF	15	M	86	100	0.29	14	0.49
IINLIIKNLSKSLTE	15	ORF6	32	46	0.31	14	0.488
LKDCVMYASAVVLLI	15	ORF1ab	3679	3693	1.8	18	0.487
TCLAYYFMRFRRAFG	15	ORF1ab	3058	3072	0.7	15	0.486
HVVAFNTLLFLMSFT	15	ORF1ab	3076	3090	0.71	15	0.485
TQQLIRAAEIRASAN	15	S	1009	1023	0.34	14	0.485
CLAYYFMRFRRAFGE	15	ORF1ab	3059	3073	0.72	15	0.484
KRRVVFNGVSFSTFE	15	ORF1ab	3162	3176	0.72	15	0.484
DAVNLLTNMFTPLIQ	15	ORF1ab	3022	3036	1.1	16	0.483
VNKFLALCADSIIIG	15	ORF1ab	682	696	1.1	16	0.483
IIWFLLLSVCLGSLI	15	ORF1ab	2230	2244	1.1	16	0.483
TDEMIAQYTSALLAG	15	S	866	880	1.1	16	0.483
LFLVLIMLIIFWFSL	15	ORF7b	18	32	0.73	15	0.483
IGLALYYPSARIVYT	15	ORF1ab	5614	5628	0.75	15	0.481
VVLLILMTARTVYDD	15	ORF1ab	3689	3703	0.38	14	0.481
IWNLDYIINLIIKNL	15	ORF6	26	40	1.5	17	0.48
FYSKWYIRVGARKSA	15	ORF8	41	55	0.76	15	0.48
FHLYLQYIRKLHDEL	15	ORF1ab	5273	5287	0.39	14	0.48
TYRRLISMMGFKMNY	15	ORF1ab	5975	5989	0.39	14	0.48
NNCYLATALLTLQQI	15	ORF1ab	1672	1686	0.39	14	0.48
LIRAAEIRASANLAA	15	S	1009	1023	0.39	14	0.48
PWNVVRIKIVQMLSD	15	ORF1ab	6083	6097	0.41	14	0.478
GPKVKYLYFIKGLNN	15	ORF1ab	4222	4236	0.41	14	0.478
VTQQLIRAAEIRASA	15	S	1001	1015	0.41	14	0.478
ASIKNFKSVLYYQNN	15	ORF1ab	5169	5183	0.8	15	0.476
IKNFKSVLYYQNNVF	15	ORF1ab	5171	5185	0.81	15	0.475
ISTKHFYWFFSNYLK	15	ORF1ab	3148	3162	0.81	15	0.475
ADVFHLYLQYIRKLH	15	ORF1ab	5270	5284	0.82	15	0.474
LALLLLDRLNQLESK	15	N	219	233	1.2	16	0.473
QYLNTLTLAVPYNMR	15	ORF1ab	6850	6864	1.2	16	0.473
PLVRKIFVDGVPFVV	15	ORF1ab	4720	4734	1.2	16	0.473
LVQMAPISAMVRMYI	15	ORF1ab	2371	2385	1.2	16	0.473
VNLLTNMFTPLIQPI	15	ORF1ab	3024	3038	1.2	16	0.473
FLPFAMGIIAMSAFA	15	ORF1ab	3611	3625	1.2	16	0.473
LDYIINLIIKNLSKS	15	ORF6	29	43	1.2	16	0.473
HFYSKWYIRVGARKS	15	ORF8	40	54	1.2	16	0.473
EMIAQYTSALLAGTI	15	S	865	879	1.2	16	0.473
RRVVFNGVSFSTFEE	15	ORF1ab	3163	3177	0.84	15	0.472
EEAARYMRSLKVPAT	15	ORF1ab	1460	1474	0.85	15	0.471

ASFRLFARTRSMWSF	15	M	98	112	0.48	14	0.471
SFKWDLTAFGLVAEW	15	ORF1ab	2313	2327	1.6	17	0.47
WLGFIAGLIAIVMVT	15	S	1214	1228	1.6	17	0.47
PTNFTISVTTEILPV	15	S	716	730	0.51	14	0.468
SVVLLSVLQQLRVES	15	ORF1ab	3866	3880	2	18	0.467
VAYFNMVYMPASWVM	15	ORF1ab	3647	3661	2	18	0.467
YFMRFRRAFGEYSHV	15	ORF1ab	3063	3077	0.89	15	0.467
YRRLISMMGFKMNYQ	15	ORF1ab	5976	5990	0.89	15	0.467
LAYYFMRFRRAFGEY	15	ORF1ab	3059	3073	0.52	14	0.467
IPTNFTISVTTEILP	15	S	715	729	0.52	14	0.467
TNFTISVTTEILPVS	15	S	716	730	0.52	14	0.467
DVSFLAHIQWMVMFT	15	ORF1ab	3119	3133	0.9	15	0.466
IIMRTFKVSIWNLDY	15	ORF6	17	31	0.9	15	0.466
QFHQKLLKSIAATRG	15	ORF1ab	4962	4976	0.53	14	0.466
QPILLLDQALVSDVG	15	ORF1ab	2567	2581	0.91	15	0.465
RKVQHMVVKAALLAD	15	ORF1ab	6235	6249	0.91	15	0.465
FRLFARTRSMWSFNP	15	M	100	114	1.3	16	0.463
PILTLTRALTAESHV	15	ORF1ab	4635	4649	1.3	16	0.463
FYFYTSKTTVASLIN	15	ORF1ab	1422	1436	1.3	16	0.463
FNYLKSPNFSKLINI	15	ORF1ab	2215	2229	1.3	16	0.463
AFLPFAMGIIAMSAF	15	ORF1ab	3610	3624	1.3	16	0.463
YASAVVLLILMTART	15	ORF1ab	3681	3695	1.3	16	0.463
LTDEMIAQYTSALLA	15	S	865	879	0.93	15	0.463
VLLILMTARTVYDDG	15	ORF1ab	3690	3704	0.56	14	0.463
FVMMSAPPAQYELKH	15	ORF1ab	1804	1818	0.56	14	0.463
GTWLTYTGAIKLDDK	15	N	328	342	0.58	14	0.461
TQYNRYLALYNKYKY	15	ORF1ab	3202	3216	0.61	14	0.458
CLEASFNYLKSPNFS	15	ORF1ab	2210	2224	0.61	14	0.458
DGFMGRIRSVYPVAS	15	ORF1ab	294	308	0.61	14	0.458
TDGTLMIERFVSLAI	15	ORF1ab	5242	5256	1	15	0.456
FKSVLYYQNNVFMSE	15	ORF1ab	5174	5188	1.4	16	0.453
TNGDFLHFLPRVFSA	15	ORF1ab	2877	2891	1.4	16	0.453
DDFVEIIKSQDLSVV	15	ORF1ab	6752	6766	1.4	16	0.453
AFEKMVSLLSVLLSM	15	ORF1ab	3907	3921	1.8	17	0.45
YFNMVYMPASWVMRI	15	ORF1ab	3649	3663	2.2	18	0.447
FNMVYMPASWVMRIM	15	ORF1ab	3650	3664	2.2	18	0.447
CNVNRFNVAITRAKV	15	ORF1ab	5876	5890	0.72	14	0.447
VCLGSLIYSTAALGV	15	ORF1ab	2238	2252	1.1	15	0.446
NKFLALCADSIIIGG	15	ORF1ab	683	697	1.1	15	0.446
LVNKFLALCADSIII	15	ORF1ab	681	695	1.1	15	0.446
LLQFAYANRNRFLYI	15	M	34	48	0.75	14	0.444
PIFFITGNTLQCIML	15	ORF1ab	3763	3777	0.76	14	0.443
SFRLFARTRSMWSFN	15	M	99	113	0.77	14	0.442
EYRLYLDAYNMMISA	15	ORF1ab	6415	6429	3	20	0.441
IFFITGNTLQCIMLV	15	ORF1ab	3768	3782	0.78	14	0.441
SSFKWDLTAFGLVAE	15	ORF1ab	2312	2326	1.9	17	0.44
PWYIWLGFIAGLIAI	15	S	1210	1224	1.9	17	0.44
RFVLALLSDLQDLKW	15	ORF1ab	4179	4193	0.79	14	0.44
YNMMISAGFSLWVYK	15	ORF1ab	6423	6437	2.3	18	0.437
QIPFAMQMAYRFNGI	15	S	895	909	0.82	14	0.437
GEVITFDNLKTLLSL	15	ORF1ab	1548	1562	1.2	15	0.436
AARVVRSIFSRTLET	15	ORF1ab	540	554	1.2	15	0.436
ARVVRSIFSRTLETA	15	ORF1ab	541	555	1.2	15	0.436

KNFKSVLYYQNNVFM	15	ORF1ab	5172	5186	1.2	15	0.436
VDAVNLLTNMFTPLI	15	ORF1ab	3021	3035	1.2	15	0.436
STVLSFCAFAVDAAK	15	ORF1ab	4261	4275	0.84	14	0.435
RRVWTLMNVLTLVYK	15	ORF1ab	3706	3720	2.7	19	0.434
LAHIQWMVMFTPLVP	15	ORF1ab	3123	3137	1.6	16	0.433
NAFLPFAMGIIAMSA	15	ORF1ab	3609	3623	1.6	16	0.433
AYFNMVYMPASWVMR	15	ORF1ab	3648	3662	2	17	0.43
RFVSLAIDAYPLTKH	15	ORF1ab	5250	5264	2	17	0.43
KTLLSLREVRTIKVF	15	ORF1ab	1557	1571	0.91	14	0.428
PILLLDQALVSDVGD	15	ORF1ab	2568	2582	0.91	14	0.428
MCQPILLLDQALVSD	15	ORF1ab	2565	2579	0.91	14	0.428
YYSLLMPILTLTRAL	15	ORF1ab	4629	4643	1.3	15	0.426
NTCTERLKLFAAETL	15	ORF1ab	5448	5462	1.3	15	0.426
DFLHFLPRVFSAVGN	15	ORF1ab	2880	2894	1.3	15	0.426
LNVGDYFVLTSHTVM	15	ORF1ab	5543	5557	1.3	15	0.426
QDLFLPFFSNVTWFH	15	S	52	66	1.3	15	0.426
TVLSFCAFAVDAAKA	15	ORF1ab	4262	4276	0.93	14	0.426
GTLIVNSVLLFLAFV	15	E	10	24	1.7	16	0.423
DKSAFVNLKQLPFFY	15	ORF1ab	6357	6371	1.7	16	0.423
HFAIGLALYYPSARI	15	ORF1ab	5614	5628	1.7	16	0.423
NDVSFLAHIQWMVMF	15	ORF1ab	3118	3132	0.96	14	0.423
SVLQQLRVESSSKLW	15	ORF1ab	3870	3884	0.97	14	0.422
KVQHMVVKAALLADK	15	ORF1ab	6236	6250	0.98	14	0.421
YVLGLAAIMQLFFSY	15	ORF1ab	2339	2353	2.1	17	0.42
ILSPLYAFASEAARV	15	ORF1ab	529	543	2.1	17	0.42
DFQVTIAEILLIIMR	15	ORF6	6	20	0.99	14	0.42
FGLFCLLNRYFRLTL	15	ORF1ab	3794	3808	1	14	0.419
EAARVVRSIFSRTLE	15	ORF1ab	539	553	1.4	15	0.416
SEAARVVRSIFSRTL	15	ORF1ab	538	552	1.4	15	0.416
TSVVLLSVLQQLRVE	15	ORF1ab	3868	3882	2.9	19	0.414
DTRYVLMDGSIIQFP	15	ORF1ab	2951	2965	1.8	16	0.413
FAMMFVKHKHAFLCL	15	ORF1ab	3624	3638	1.8	16	0.413
NLWNTFTRLQSLENV	15	ORF1ab	6443	6457	1.8	16	0.413
TVAYFNMVYMPASWV	15	ORF1ab	3646	3660	2.2	17	0.41
YLDAYNMMISAGFSL	15	ORF1ab	6419	6433	1.1	14	0.409
FAIGLALYYPSARIV	15	ORF1ab	5612	5626	1.1	14	0.409
NLVQMAPISAMVRMY	15	ORF1ab	2369	2383	1.1	14	0.409
KLKTLVATAEAELAK	15	ORF1ab	2608	2622	1.1	14	0.409
ATEETFKLSYGIATV	15	ORF1ab	5464	5478	1.5	15	0.406
NFKSVLYYQNNVFMS	15	ORF1ab	5170	5184	1.5	15	0.406
TQDLFLPFFSNVTWF	15	S	51	65	1.5	15	0.406
YNLWNTFTRLQSLEN	15	ORF1ab	6442	6456	1.9	16	0.403
CTSVVLLSVLQQLRV	15	ORF1ab	3867	3881	3.4	20	0.401
WTLMNVLTLVYKVYY	15	ORF1ab	3706	3720	1.19	14	0.4
AALCTFLLNKEMYLK	15	ORF1ab	3178	3192	1.2	14	0.399
SIKNFKSVLYYQNNV	15	ORF1ab	5170	5184	1.2	14	0.399
LDAYNMMISAGFSLW	15	ORF1ab	6420	6434	1.6	15	0.396
VQHMVVKAALLADKF	15	ORF1ab	6234	6248	1.6	15	0.396
TCTERLKLFAAETLK	15	ORF1ab	5449	5463	1.3	14	0.389
YWFFSNYLKRRVVFN	15	ORF1ab	3154	3168	1.3	14	0.389
MGHFAWWTAFVTNVN	15	ORF1ab	6979	6993	1.3	14	0.389
PDTRYVLMDGSIIQF	15	ORF1ab	2950	2964	1.7	15	0.386
FDKSAFVNLKQLPFF	15	ORF1ab	6356	6370	1.7	15	0.386

LEEAARYMRSLKVPA	15	ORF1ab	1459	1473	1.7	15	0.386
ISSFKWDLTAFGLVA	15	ORF1ab	2311	2325	1.7	15	0.386
LFLMSFTVLCLTPVY	15	ORF1ab	3083	3097	1.7	15	0.386
NVLSTFISAARQGFV	15	ORF1ab	2628	2642	3.2	19	0.384
YGARFYFYTSKTTVA	15	ORF1ab	1418	1432	2.1	16	0.383
DMILSLLSKGRLIIR	15	ORF1ab	7067	7081	2.1	16	0.383
LTILTSLLVLVQSTQ	15	ORF1ab	3585	3599	2.1	16	0.383
FEKMVSLLSVLLSMQ	15	ORF1ab	3905	3919	2.5	17	0.38
NRYFRLTLGVYDYLV	15	ORF1ab	3797	3811	2.5	17	0.38
NSIDAFKLNIKLLGV	15	ORF1ab	3833	3847	1.4	14	0.379
AFAMMFVKHKHAFLC	15	ORF1ab	3622	3636	1.8	15	0.376
PNFVFPLNSIIKTIQ	15	ORF1ab	271	285	2.2	16	0.373
LLLTILTSLLVLVQS	15	ORF1ab	3583	3597	2.2	16	0.373
AISMWALIISVTSNY	15	ORF1ab	3727	3741	2.6	17	0.37
ASFYYVWKSYVHVVD	15	ORF1ab	2388	2402	2.6	17	0.37
SLLVLVQSTQWSLFF	15	ORF1ab	3590	3604	1.5	14	0.369
STQDLFLPFFSNVTW	15	S	50	64	1.5	14	0.369
HSTQDLFLPFFSNVT	15	S	49	63	1.5	14	0.369
FIEDLLFNKVTLADA	15	S	818	832	1.5	14	0.369
MVLGSLAATVRLQAG	15	ORF1ab	4241	4255	1.9	15	0.366
RVVRSIFSRTLETAQ	15	ORF1ab	542	556	2.3	16	0.363
SILSPLYAFASEAAR	15	ORF1ab	528	542	2.3	16	0.363
KSILSPLYAFASEAA	15	ORF1ab	527	541	2.3	16	0.363
PNFSKLINIIIWFLL	15	ORF1ab	2221	2235	2.7	17	0.36
LGFIAGLIAIVMVTI	15	S	1218	1232	1.6	14	0.359
TITQMNLKYAISAKN	15	ORF1ab	4930	4944	2	15	0.356
YKIEELFYSYATHSD	15	ORF1ab	6286	6300	2	15	0.356
TRYVLMDGSIIQFPN	15	ORF1ab	2952	2966	2	15	0.356
CLLNRYFRLTLGVYD	15	ORF1ab	3798	3812	3.5	19	0.354
AFDKSAFVNLKQLPF	15	ORF1ab	6352	6366	1.7	14	0.349
CFKMFYKGVITHDVS	15	ORF1ab	5795	5809	2.1	15	0.346
LWNTFTRLQSLENVA	15	ORF1ab	6444	6458	2.1	15	0.346
WPWYIWLGFIAGLIA	15	S	1213	1227	2.1	15	0.346
YSLLMPILTLTRALT	15	ORF1ab	4627	4641	2.5	16	0.343
SQYSLRLIDAMMFTS	15	ORF1ab	574	588	2.5	16	0.343
TKGGRFVLALLSDLQ	15	ORF1ab	4175	4189	2.5	16	0.343
RYFRLTLGVYDYLVS	15	ORF1ab	3799	3813	2.5	16	0.343
LNRYFRLTLGVYDYL	15	ORF1ab	3800	3814	2.9	17	0.34
LSLREVRTIKVFTTV	15	ORF1ab	1560	1574	1.8	14	0.339
LLLDDFVEIIKSQDL	15	ORF1ab	6749	6763	1.8	14	0.339
FCLLNRYFRLTLGVY	15	ORF1ab	3797	3811	1.8	14	0.339
TNDVSFLAHIQWMVM	15	ORF1ab	3116	3130	1.8	14	0.339
LLNRYFRLTLGVYDY	15	ORF1ab	3795	3809	3.3	18	0.337
FLHFLPRVFSAVGNI	15	ORF1ab	2881	2895	2.2	15	0.336
GHFAWWTAFVTNVNA	15	ORF1ab	6980	6994	2.2	15	0.336
LLTILTSLLVLVQST	15	ORF1ab	3584	3598	2.2	15	0.336
MIAQYTSALLAGTIT	15	S	861	875	2.2	15	0.336
LSFKELLVYAADPAM	15	ORF1ab	4758	4772	3	17	0.33
HANEYRLYLDAYNMM	15	ORF1ab	6412	6426	1.9	14	0.329
FDNLKTLLSLREVRT	15	ORF1ab	1553	1567	2.3	15	0.326
YLNTLTLAVPYNMRV	15	ORF1ab	6851	6865	2.3	15	0.326
SHFAIGLALYYPSAR	15	ORF1ab	5609	5623	2.3	15	0.326
FLMSFTVLCLTPVYS	15	ORF1ab	3085	3099	2.7	16	0.323

AYYVGYLQPRTFLLK	15	S	262	276	2.7	16	0.323
VWTLMNVLTLVYKVY	15	ORF1ab	3708	3722	1.99	14	0.32
IFYLITPVHVMSKHT	15	ORF1ab	2786	2800	2.4	15	0.316
INDMILSLLSKGRLI	15	ORF1ab	7065	7079	2.45	15	0.311
GKSHFAIGLALYYPS	15	ORF1ab	5611	5625	3.2	17	0.31
FKMFYKGVITHDVSS	15	ORF1ab	5792	5806	2.1	14	0.309
GTTFTYASALWEIQQ	15	ORF1ab	4082	4096	2.1	14	0.309
ILHCANFNVLFSTVF	15	ORF1ab	4699	4713	2.5	15	0.306
NFSKLINIIIWFLLL	15	ORF1ab	2221	2235	2.5	15	0.306
MILSLLSKGRLIIRE	15	ORF1ab	7068	7082	2.9	16	0.303
QYSLRLIDAMMFTSD	15	ORF1ab	575	589	2.9	16	0.303
AAYYVGYLQPRTFLL	15	S	261	275	2.9	16	0.303
HGVVFLHVTYVPAQE	15	S	1050	1064	2.9	16	0.303
VVFLHVTYVPAQEKN	15	S	1057	1071	2.9	16	0.303
LMSFTVLCLTPVYSF	15	ORF1ab	3086	3100	3.3	17	0.3
TLAILTALRLCAYCC	15	E	30	44	2.2	14	0.299
TTNGDFLHFLPRVFS	15	ORF1ab	2876	2890	2.2	14	0.299
SFKELLVYAADPAMH	15	ORF1ab	4759	4773	2.6	15	0.296
QHMVVKAALLADKFP	15	ORF1ab	6235	6249	2.6	15	0.296
GMVLGSLAATVRLQA	15	ORF1ab	4240	4254	2.6	15	0.296
YYVGYLQPRTFLLKY	15	S	265	279	2.6	15	0.296
SRLSFKELLVYAADP	15	ORF1ab	4756	4770	3.4	17	0.29
KSHFAIGLALYYPSA	15	ORF1ab	5612	5626	3.4	17	0.29
NFVFPLNSIIKTIQP	15	ORF1ab	272	286	2.3	14	0.289
TISSFKWDLTAFGLV	15	ORF1ab	2310	2324	2.3	14	0.289
YADVFHLYLQYIRKL	15	ORF1ab	5269	5283	2.3	14	0.289
CFVLAAVYRINWITG	15	M	64	78	2.7	15	0.286
NHNFLVQAGNVQLRV	15	ORF1ab	3325	3339	2.7	15	0.286
HNFLVQAGNVQLRVI	15	ORF1ab	3327	3341	2.7	15	0.286
YRLYLDAYNMMISAG	15	ORF1ab	6416	6430	3.1	16	0.283
RVWTLMNVLTLVYKV	15	ORF1ab	3707	3721	4.6	20	0.281
EHFIETISLAGSYKD	15	ORF1ab	1499	1513	3.5	17	0.28
DGEVITFDNLKTLLS	15	ORF1ab	1547	1561	2.5	14	0.269
GVVFLHVTYVPAQEK	15	S	1057	1071	2.9	15	0.266
HFAWWTAFVTNVNAS	15	ORF1ab	6984	6998	3.3	16	0.263
PEEHFIETISLAGSY	15	ORF1ab	1497	1511	3.3	16	0.263
VSLDNVLSTFISAAR	15	ORF1ab	2624	2638	3.3	16	0.263
FTRLQSLENVAFNVV	15	ORF1ab	6445	6459	3.7	17	0.26
VYSFLPGVYSVIYLY	15	ORF1ab	3097	3111	2.6	14	0.259
CPNFVFPLNSIIKTI	15	ORF1ab	270	284	2.6	14	0.259
YFVLTSHTVMPLSAP	15	ORF1ab	5548	5562	2.6	14	0.259
FMRFRRAFGEYSHVV	15	ORF1ab	3064	3078	2.6	14	0.259
LLILMTARTVYDDGA	15	ORF1ab	3687	3701	2.6	14	0.259
KCTSVVLLSVLQQLR	15	ORF1ab	3866	3880	3.8	17	0.25
ISQYSLRLIDAMMFT	15	ORF1ab	573	587	3.8	17	0.25
RYVLMDGSIIQFPNT	15	ORF1ab	2953	2967	2.7	14	0.249
TTKGGRFVLALLSDL	15	ORF1ab	4171	4185	2.7	14	0.249
DNVLSTFISAARQGF	15	ORF1ab	2627	2641	4.2	18	0.247
RLSFKELLVYAADPA	15	ORF1ab	4757	4771	3.5	16	0.243
TGKSHFAIGLALYYP	15	ORF1ab	5607	5621	3.5	16	0.243
LVKPSFYVYSRVKNL	15	E	51	65	2.8	14	0.239
VKPSFYVYSRVKNLN	15	E	52	66	2.8	14	0.239
VSLLSVLLSMQGAVD	15	ORF1ab	3912	3926	2.8	14	0.239

MSFTVLCLTPVYSFL	15	ORF1ab	3087	3101	2.8	14	0.239
EEHFIETISLAGSYK	15	ORF1ab	1498	1512	2.8	14	0.239
YSLRLIDAMMFTSDL	15	ORF1ab	576	590	3.6	16	0.233
VFRSSVLHSTQDLFL	15	S	42	56	2.9	14	0.229
SLDNVLSTFISAARQ	15	ORF1ab	2625	2639	3.3	15	0.226
EKMVSLLSVLLSMQG	15	ORF1ab	3909	3923	3.7	16	0.223
VLQQLRVESSSKLWA	15	ORF1ab	3875	3889	3	14	0.219
VLGLAAIMQLFFSYF	15	ORF1ab	2340	2354	3	14	0.219
LTSMKYFVKIGPERT	15	ORF1ab	6117	6131	3.5	15	0.206
SFYYVWKSYVHVVDG	15	ORF1ab	2389	2403	3.5	15	0.206
EQYVFCTVNALPETT	15	ORF1ab	5677	5691	3.9	16	0.203
GISQYSLRLIDAMMF	15	ORF1ab	572	586	4.65	18	0.202
LTWICLLQFAYANRN	15	M	29	43	3.95	16	0.198
KFVRIQPGQTFSVLA	15	ORF1ab	3364	3378	3.6	15	0.196
SSRLSFKELLVYAAD	15	ORF1ab	4752	4766	3.6	15	0.196
LATVAYFNMVYMPAS	15	ORF1ab	3644	3658	4.5	17	0.18
HMVVKAALLADKFPV	15	ORF1ab	6239	6253	3.5	14	0.169
KTVLKKCKSAFYILP	15	ORF1ab	1343	1357	3.9	15	0.166
IAYIICISTKHFYWF	15	ORF1ab	3142	3156	3.8	14	0.139
MMFVKHKHAFLCLFL	15	ORF1ab	3626	3640	3.8	14	0.139
LEQYVFCTVNALPET	15	ORF1ab	5676	5690	4.3	15	0.126
GTGKSHFAIGLALYY	15	ORF1ab	5609	5623	4.33	15	0.123
ATVAYFNMVYMPASW	15	ORF1ab	3645	3659	5.1	17	0.12
YLKSPNFSKLINIII	15	ORF1ab	2217	2231	4.4	15	0.116
VKCTSVVLLSVLQQL	15	ORF1ab	3865	3879	4.4	15	0.116
QYVFCTVNALPETTA	15	ORF1ab	5678	5692	4.1	14	0.109
SPNFSKLINIIIWFL	15	ORF1ab	2220	2234	4.2	14	0.099
AKTVLKKCKSAFYIL	15	ORF1ab	1342	1356	4.3	14	0.089
KLQFTSLEIPRRNVA	15	ORF1ab	5908	5922	4.7	15	0.086
TLEQYVFCTVNALPE	15	ORF1ab	5671	5685	5	15	0.056
VLSTFISAARQGFVD	15	ORF1ab	2629	2643	5	15	0.056
FYYVWKSYVHVVDGC	15	ORF1ab	2390	2404	4.7	14	0.049
TWICLLQFAYANRNR	15	M	30	44	5.5	16	0.043
LTSLLVLVQSTQWSL	15	ORF1ab	3588	3602	5.2	15	0.036
SVCLGSLIYSTAALG	15	ORF1ab	2237	2251	5.2	15	0.036
KEIIFLEGETLPTEV	15	ORF1ab	737	751	4.9	14	0.029
PLIQPIGALDISASI	15	ORF1ab	3033	3047	5	14	0.019
AMMFVKHKHAFLCLF	15	ORF1ab	3625	3639	5.4	15	0.016
MVSLLSVLLSMQGAV	15	ORF1ab	3911	3925	5.4	15	0.016
ITIAYIICISTKHFY	15	ORF1ab	3139	3153	5.5	14	-0.031
TRLQSLENVAFNVVN	15	ORF1ab	6449	6463	5.5	14	-0.031
IQPIGALDISASIVA	15	ORF1ab	3035	3049	5.9	14	-0.071

Table S3. 15 mers CD4+ epitopes with overlapping of 10 amino acid residues

epitope	length	protein_clean	start	end	percentile_rank	promiscuity	final_score
PNMLRIMASLVLARK	15	ORF1ab	5016	5030	0.01	22	0.814
MWLSYFIASFRLFAR	15	M	91	105	0.08	22	0.807
TERLKLFAAETLKAT	15	ORF1ab	5451	5465	0.15	21	0.763
LFFFLYENAFLPFAM	15	ORF1ab	3601	3615	0.02	20	0.739
ILASFSASTSAFVET	15	ORF1ab	476	490	0.02	20	0.739
SLFFFLYENAFLPFA	15	ORF1ab	3601	3615	0.03	20	0.738
KHFYWFFSNYLKRRV	15	ORF1ab	3151	3165	0.14	20	0.727
QTFFKLVNKFLALCA	15	ORF1ab	676	690	0.19	20	0.722
LCLFLLPSLATVAYF	15	ORF1ab	3636	3650	0.03	19	0.701
EFYAYLRKHFSMMIL	15	ORF1ab	5136	5150	0.05	19	0.699
WLIINLVQMAPISAM	15	ORF1ab	2366	2380	0.81	21	0.697
NSWLMWLIINLVQMA	15	ORF1ab	2361	2375	0.1	19	0.694
MGIIAMSAFAMMFVK	15	ORF1ab	3616	3630	1.6	23	0.692
FLLVTLAILTALRLC	15	E	26	40	0.38	19	0.666
ESPFVMMSAPPAQYE	15	ORF1ab	1801	1815	0.01	18	0.666
LIIMRTFKVSIWNLD	15	ORF6	16	30	0.78	20	0.663
LAFVVFLLVTLAILT	15	E	21	35	0.06	18	0.661
VLSFCAFAVDAAKAY	15	ORF1ab	4266	4280	0.13	18	0.654
HWLLLTILTSLLVLV	15	ORF1ab	3581	3595	0.16	18	0.651
WYIWLGFIAGLIAIV	15	S	1211	1225	0.58	19	0.646
MFHLVDFQVTIAEIL	15	ORF6	1	15	0.8	19	0.624
KDCVMYASAVVLLIL	15	ORF1ab	3676	3690	1.2	20	0.621
LIVTALRANSAVKLQ	15	ORF1ab	4126	4140	0.09	17	0.621
AYLRKHFSMMILSDD	15	ORF1ab	5136	5150	0.88	19	0.616
VDSYYSLLMPILTLT	15	ORF1ab	4626	4640	0.16	17	0.614
GDYFVLTSHTVMPLS	15	ORF1ab	5546	5560	0.54	18	0.613
PKYKFVRIQPGQTFS	15	ORF1ab	3361	3375	0.2	17	0.610
IAEILLIIMRTFKVS	15	ORF6	11	25	0.22	17	0.608
TQLCQYLNTLTLAVP	15	ORF1ab	6846	6860	0.69	18	0.598
EETFKLSYGIATVRE	15	ORF1ab	5466	5480	0.7	18	0.597
SPLYAFASEAARVVR	15	ORF1ab	531	545	0.73	18	0.594
RFYFYTSKTTVASLI	15	ORF1ab	1421	1435	0.74	18	0.593
RAMPNMLRIMASLVL	15	ORF1ab	5016	5030	0.01	16	0.592
TFKVSIWNLDYIINL	15	ORF6	21	35	0.02	16	0.591
AYYFMRFRRAFGEYS	15	ORF1ab	3061	3075	0.4	17	0.590
SAFYILPSIISNEKQ	15	ORF1ab	1351	1365	0.42	17	0.588
SLLMPILTLTRALTA	15	ORF1ab	4631	4645	0.82	18	0.585
FIASFRLFARTRSMW	15	M	96	110	0.48	17	0.582
FLLFLVLIMLIIFWF	15	ORF7b	16	30	0.19	16	0.574
GEYSHVVAFNTLLFL	15	ORF1ab	3071	3085	1.7	20	0.571
FSYFAVHFISNSWLM	15	ORF1ab	2351	2365	0.61	17	0.569
LRKHFSMMILSDDAV	15	ORF1ab	5141	5155	0.64	17	0.566
NRNRFLYIIKLIFLW	15	M	41	55	0.65	17	0.565
YIINLIIKNLSKSLT	15	ORF6	31	45	0.3	16	0.563
DAYNMMISAGFSLWV	15	ORF1ab	6421	6435	0.71	17	0.559
SYFAVHFISNSWLMW	15	ORF1ab	2351	2365	0.39	16	0.554
LCFLAFLLFLVLIML	15	ORF7b	11	25	0.02	15	0.554
SVLLFLAFVVFLLVT	15	E	16	30	0.04	15	0.552
NTLLFLMSFTVLCLT	15	ORF1ab	3081	3095	0.79	17	0.551
IMQLFFSYFAVHFIS	15	ORF1ab	2346	2360	0.05	15	0.551
ISMWALIISVTSNYS	15	ORF1ab	3731	3745	0.44	16	0.549
YILFTRFFYVLGLAA	15	ORF1ab	2331	2345	0.08	15	0.548
DCVMYASAVVLLILM	15	ORF1ab	3681	3695	1.2	18	0.547
KQLIKVTLVFLFVAA	15	ORF1ab	2771	2785	0.13	15	0.543
TLIVNSVLLFLAFVV	15	E	11	25	0.53	16	0.540

NVGDYFVLTSHTVMP	15	ORF1ab	5541	5555	0.91	17	0.539
CQPILLLDQALVSDV	15	ORF1ab	2566	2580	0.91	17	0.539
NKEMYLKLRSDVLLP	15	ORF1ab	3186	3200	0.56	16	0.537
TPKYKFVRIQPGQTF	15	ORF1ab	3361	3375	0.22	15	0.534
ASAVVLLILMTARTV	15	ORF1ab	3686	3700	0.24	15	0.532
MFVFLVLLPLVSSQC	15	S	1	15	0.24	15	0.532
YLTFYLTNDVSFLAH	15	ORF1ab	3111	3125	0.7	16	0.523
EAARYMRSLKVPATV	15	ORF1ab	1461	1475	0.33	15	0.523
HSLSHFVNLDNLRAN	15	ORF1ab	2516	2530	0.34	15	0.522
GSLIYSTAALGVLMS	15	ORF1ab	2241	2255	0.75	16	0.518
DVFHLYLQYIRKLHD	15	ORF1ab	5271	5285	0.38	15	0.518
WPQIAQFAPSASAFF	15	N	301	315	0.01	14	0.518
SLLIVNNATNVVIKV	15	S	116	130	0.01	14	0.518
VVLSFELLHAPATVC	15	S	511	525	0.03	14	0.516
FGEYSHVVAFNTLLF	15	ORF1ab	3071	3085	1.9	19	0.514
NDMILSLLSKGRLII	15	ORF1ab	7066	7080	1.9	19	0.514
MNLKYAISAKNRART	15	ORF1ab	4931	4945	0.08	14	0.511
QCFKMFYKGVITHDV	15	ORF1ab	5791	5805	0.83	16	0.510
GLVASIKNFKSVLYY	15	ORF1ab	5166	5180	0.46	15	0.510
LINIIIWFLLLSVCL	15	ORF1ab	2226	2240	0.09	14	0.510
DAALALLLLDRLNQL	15	N	216	230	0.85	16	0.508
INIIIWFLLLSVCLG	15	ORF1ab	2226	2240	0.14	14	0.505
FYVYSRVKNLNSSRV	15	E	56	70	0.52	15	0.504
EAFEKMVSLLSVLLS	15	ORF1ab	3906	3920	1.3	17	0.500
KELLVYAADPAMHAA	15	ORF1ab	4761	4775	0.2	14	0.499
QPYRVVVLSFELLHA	15	S	506	520	0.24	14	0.495
CVPLNIIPLTTAAKL	15	ORF1ab	4056	4070	0.27	14	0.492
WICLLQFAYANRNRF	15	M	31	45	2.5	20	0.491
CLVGLMWLSYFIASF	15	M	86	100	0.29	14	0.490
HVVAFNTLLFLMSFT	15	ORF1ab	3076	3090	0.71	15	0.485
TDEMIAQYTSALLAG	15	S	866	880	1.1	16	0.483
IWNLDYIINLIIKNL	15	ORF6	26	40	1.5	17	0.480
FYSKWYIRVGARKSA	15	ORF8	41	55	0.76	15	0.480
VTQQLIRAAEIRASA	15	S	1001	1015	0.41	14	0.478
IKNFKSVLYYQNNVF	15	ORF1ab	5171	5185	0.81	15	0.475
LVQMAPISAMVRMYI	15	ORF1ab	2371	2385	1.2	16	0.473
FLPFAMGIIAMSAFA	15	ORF1ab	3611	3625	1.2	16	0.473
PTNFTISVTTEILPV	15	S	716	730	0.51	14	0.468
SVVLLSVLQQLRVES	15	ORF1ab	3866	3880	2	18	0.467
YRRLISMMGFKMNYQ	15	ORF1ab	5976	5990	0.89	15	0.467
TNFTISVTTEILPVS	15	S	716	730	0.52	14	0.467
YASAVVLLILMTART	15	ORF1ab	3681	3695	1.3	16	0.463
CNVNRFNVAITRAKV	15	ORF1ab	5876	5890	0.72	14	0.447
LVNKFLALCADSIII	15	ORF1ab	681	695	1.1	15	0.446
ARVVRSIFSRTLETA	15	ORF1ab	541	555	1.2	15	0.436
VDAVNLLTNMFTPLI	15	ORF1ab	3021	3035	1.2	15	0.436
STVLSFCAFAVDAAK	15	ORF1ab	4261	4275	0.84	14	0.435
RRVWTLMNVLTLVYK	15	ORF1ab	3706	3720	2.7	19	0.434
KVQHMVVKAALLADK	15	ORF1ab	6236	6250	0.98	14	0.421
DFQVTIAEILLIIMR	15	ORF6	6	20	0.99	14	0.420
DTRYVLMDGSIIQFP	15	ORF1ab	2951	2965	1.8	16	0.413
TVAYFNMVYMPASWV	15	ORF1ab	3646	3660	2.2	17	0.410
TQDLFLPFFSNVTWF	15	S	51	65	1.5	15	0.406
WTLMNVLTLVYKVYY	15	ORF1ab	3706	3720	1.19	14	0.400
ISSFKWDLTAFGLVA	15	ORF1ab	2311	2325	1.7	15	0.386
FDKSAFVNLKQLPFF	15	ORF1ab	6356	6370	1.7	15	0.386
PNFVFPLNSIIKTIQ	15	ORF1ab	271	285	2.2	16	0.373

MVLGSLAATVRLQAG	15	ORF1ab	4241	4255	1.9	15	0.366
PNFSKLINIIIWFLL	15	ORF1ab	2221	2235	2.7	17	0.360
YKIEELFYSYATHSD	15	ORF1ab	6286	6300	2	15	0.356
TNDVSFLAHIQWMVM	15	ORF1ab	3116	3130	1.8	14	0.339
FLHFLPRVFSAVGNI	15	ORF1ab	2881	2895	2.2	15	0.336
MIAQYTSALLAGTIT	15	S	861	875	2.2	15	0.336
YLNTLTLAVPYNMRV	15	ORF1ab	6851	6865	2.3	15	0.326
IFYLITPVHVMSKHT	15	ORF1ab	2786	2800	2.4	15	0.316
GKSHFAIGLALYYPS	15	ORF1ab	5611	5625	3.2	17	0.310
NFSKLINIIIWFLLL	15	ORF1ab	2221	2235	2.5	15	0.306
AAYYVGYLQPRTFLL	15	S	261	275	2.9	16	0.303
LMSFTVLCLTPVYSF	15	ORF1ab	3086	3100	3.3	17	0.300
TTNGDFLHFLPRVFS	15	ORF1ab	2876	2890	2.2	14	0.299
SRLSFKELLVYAADP	15	ORF1ab	4756	4770	3.4	17	0.290
YRLYLDAYNMMISAG	15	ORF1ab	6416	6430	3.1	16	0.283
KCTSVVLLSVLQQLR	15	ORF1ab	3866	3880	3.8	17	0.250
TTKGGRFVLALLSDL	15	ORF1ab	4171	4185	2.7	14	0.249
LVKPSFYVYSRVKNL	15	E	51	65	2.8	14	0.239
YSLRLIDAMMFTSDL	15	ORF1ab	576	590	3.6	16	0.233
MMFVKHKHAFLCLFL	15	ORF1ab	3626	3640	3.8	14	0.139
LEQYVFCTVNALPET	15	ORF1ab	5676	5690	4.3	15	0.126
TLEQYVFCTVNALPE	15	ORF1ab	5671	5685	5	15	0.056
MVSLLSVLLSMQGAV	15	ORF1ab	3911	3925	5.4	15	0.016

Table S4: List of the selected $CD4^+$ T cell epitopes

	Epitopes	Coverage PR10%	Protein	Start	End	Percentile rank	Promiscuity	Final scores
1	LFTRFFYVLGLAAIMQLFF	93.06	ORF1ab	2333	2351	0.24-0.67	22-26	0.938-0.777
2	TERLKLFAAETLKATEE	91.83	ORF1ab	5451	5467	0.15-0.2	21-23	0.837-0.758
3	VTLVFLFVAAIFYLITPVHVM	95.75	ORF1ab	2775	2796	0.19-0.63	20-23	0.832-0.722
4	MPNMLRIMASLVLARKHT	88.22	ORF1ab	5018	5034	0.01-0.2	21-22	0.814-0758
5	LMWLSYFIASFRLFARTR	94.61	M	90	107	0.05-0.35	19-22	0.807-0.669
6	NSWLMWLIINLVQMAPISAM	72.17	ORF1ab	2361	2380	0.1-1.19	19-22	0.755-0.694
7	LMIERFVSLAIDAYPLT	93.53	ORF1ab	5242	5262	0.65-0.52	21-22	0.750-0.696
8	HLVDFQVTIAEILLI	93.44	ORF6	3	17	0.67	22	0.748
9	VFLLVTLAILTALRLCAY	90.93	E	25	42	0.38	19-21	0.740-0.666
10	IILASFSASTSAFVET	75.96	ORF1ab	475	490	0.01-0.02	20-20	0.740-0.739
11	FAWWTAFVTNVNASS	62.88	ORF1ab	6982	6996	0.01-0.01	20	0.740
12	SLFFFLYENAFLPFAM	93.43	ORF1ab	3601	3615	0.02-0.07	19-20	0.739-0.697
13	CTERLKLFAAETLKA	92.95	ORF1ab	5450	5464	0.41	21	0.737
14	QLIKVTLVFLFVAAIFYL	90.68	ORF1ab	2772	2789	0.04-0.22	20-20	0.737-0.719
15	FLCLFLLPSLATVAY	86.54	ORF1ab	3635	3649	0.05	20	0.736
16	FLAFVVFLLVTLAILTAL	87.88	E	20	37	0.06	20	0.735

Table S5 CD4 prediction for the 16 epitopes in the IEDB platform considering the full set Class II alleles

	Class II epitopes	PR<10%	PR<20%	promiscuity	DPA1*01:03/DPB1*02:01	DPA1*01:03/DPB1*04:01	DPA1*02:01/DPB1*01:01	DPA1*02:01/DPB1*05:01	DPA1*02:01/DPB1*14:01	DPA1*03:01/DPB1*04:02	DQA1*01:01/DQB1*05:01	DQA1*01:02/DQB1*06:02	DQA1*03:01/DQB1*03:02	DQA1*04:01/DQB1*04:02	DQA1*05:01/DQB1*02:01	DQA1*05:01/DQB1*03:01
1	LFTRFFYVLGLAAIMQLFF	12	9	21	2	10	4.9	7.7	18	7.2	7.7	4.6	16	6.2	2.5	16
2	TERLKLFAAETLKATEE	15	5	20	12	2.1	3.3	3.4	0.25	4.6	13	3.8	9.1	7	-	14
3	VTLVFLFVAAIFYLITPVHVM	17	4	21	0.34	4.9	0.5	4.8	-	1.7	4	-	8.9	-	3.1	-
4	MPNMLRIMASLVLARKHT	16	3	19	-	8	16	16	0.49	12	-	1.3	-	-	-	-
5	LMWLSYFIASFRLFARTR	16	4	20	0.18	0.47	0.79	1.1	4	4.7	2.3	-	13	19	-	-
6	NSWLMWLIINLVQMAPISAM	13	5	18	-	17	8.6	-	16	4.1	0.23	-	-	-	-	-
7	LMIERFVSLAIDAYPLT	16	5	21	3.9	2.7	7.8	15	1.1	2.7	3	-	6	2.1	1.6	-
8	HLVDFQVTIAEILLI	13	9	22	4.5	1.3	2	6.7	6.4	0.67	12	5.2	7.9	1.5	2.2	-
9	VFLLVTLAILTALRLCAY	14	4	18	3.5	17	1.7	3.3	-	0.46	-	4.2	-	-	18	-
10	IILASFSASTSAFVET	11	8	19	11	14	18	-	2.4	-	-	17	3.2	6.2	-	3
	FAWWTAFVTNVNASS	10	12	22	16	8.9	11	19	13	14	16	-	18	11	-	11
12	SLFFFLYENAFLPFAM	15	4	19	0.74	0.01	0.47	0.94	3.7	1.1	0.74	-	18	8.9	0.59	-
13	CTERLKLFAAETLKA	15	5	20	9.9	3.3	2.1	2.1	0.41	5	7.8	5.3	19	17	14	-
14	QLIKVTLVFLFVAAIFYL	14	5	19	2.8	-	0.79	0.03	-	2.4	5.8	8.5	6.9	-	2.5	-
15	FLCLFLLPSLATVAY	14	6	20	4.3	3.7	12	18	6.6	2	3.1	-	-	19	-	16
16	FLAFVVFLLVTLAILTAL	12	4	16	0.08	-	0.14	3.9	-	0.07	-	7.7	-	-	3.2	-

	Class II epitopes	DRB1*01:01	DRB1*03:01	DRB1*04:01	DRB1*04:05	DRB1*07:01	DRB1*08:02	DRB1*09:01	DRB1*11:01	DRB1*12:01	DRB1*13:02	DRB1*15:01	DRB3*01:01	DRB3*02:02	DRB4*01:01	DRB5*01:01
1	LFTRFFYVLGLAAIMQLFF	0.65	-	-	16	14	14	5.9	7.2	3.55	-	14	-	-	-	16
2	TERLKLFAAETLKATEE	7.5	-	2.8	15	7.9	-	1.9	-	18	-	4.7	-	7.7	4.2	-
3	VTLVFLFVAAIFYLITPVHVM	1.6	-	3.6	5.5	1.2	11	4.2	8.9	7.53	6	15	12	15	-	3.5
4	MPNMLRIMASLVLARKHT	2.3	0.94	4	4.9	4.8	6.2	-	2.4	8.8	6.1	0.01	-	7.6	4.3	3.7
5	LMWLSYFIASFRLFARTR	8.7	3.6	-	16	6	6.6	-	2.4	8.55	-	2.3	8.9	13	-	3.6
6	NSWLMWLIINLVQMAPISAM	7.3	11	5.3	4.6	11	4.9	7.4	-	1.16	8.9	8.1	-	10	4.3	-
7	LMIERFVSLAIDAYPLT	14	-	8	7.3	2.5	13	12	-	5	-	1.7	2.2	16	6.3	-
8	HLVDFQVTIAEILLI	17	18	19	16	1.3	-	5.8	-	-	16	-	9.5	15	17	17
9	VFLLVTLAILTALRLCAY	0.69	9.4	2.3	4	2.3	11	-	5.3	1.01	18	3.9	-	-	-	9.9
10	IILASFSASTSAFVET	14	-	0.51	3.5	0.57	2.9	0.04	-	-	7.7	12	14	1.6	-	11
	FAWWTAFVTNVNASS	6.8	-	0.24	2.1	17	1.6	8.4	8.9	-	10	-	12	0.01	-	-
12	SLFFFLYENAFLPFAM	1.2	-	0.82	2.8	-	-	16	-	10.55	19	4.8	2.2	4.7	-	-
13	CTERLKLFAAETLKA	13	-	1.7	8.9	4.5	-	0.93	-	11.35	-	2.9	-	9.8	2.3	13
14	QLIKVTLVFLFVAAIFYL	5	-	10	8.3	3.8	2.4	-	-	18.07	15	12	10	-	18	4
15	FLCLFLLPSLATVAY	0.05	-	13	4.9	13	9.2	9	2.9	1.57	9.6	4.1	-	6.5	-	-
16	FLAFVVFLLVTLAILTAL	2.8	15	2.3	2.7	4.4	11	-	12	-	-	3.3	-	-	7.3	18

Table S6. Number of CD4$^+$ epitopes binding with high affinity to the most frequent DR, DQ and DP alleles.

allelles	Phenotype frequency in World population (%)	Number of epitopes binding with PR < 10 %
DRB4*01:01	41.8	6
DPA1*03:01/DPB1 04:02	41.6	13
DPA1*01:03/DPB1 04:01	36.2	10
DQA1*05:01/DQB1*03:01	35	1
DRB3*02:02	34.3	7
DPA1*02:01/ DPB1*05:01	21.7	10
DQA1*0301/DQB1*03:02	19	6
DPA1*01:03/DPB1*02:01	17.5	10
DPA1*02:01/DPB1*01:01	16	12
DRB5*01:01	16	5
DQA1*01:02/DQB1*06:02	14.6	8
DRB1*03:01	13.7	3
DRB1*07:01	13.5	11
DQA1*04:01/DQB1*04:02	12.8	5
DRB1*15:01	12.2	10
DRB1*11:01	11.8	7
DQA1*05:01/DQB1*02:01	11.3	7
DRB1*13:02	7.7	5

Table S7. List of the 47 CD8+ epitopes filtered using final scores in decreasing order

epitope	Population coverage PR=0.5%SB	Population coverage PR=1%WB	length	protein	start	end	percentile rank	weak binders (PR<1%)	strong binders (PR<0.5%)	final_score	tested	dominant
VVYRGTTTY	66.42	67.9	9	ORF1ab	5533	5541	0.01	12	11	0.451	VVYRGTTTY	yes
KLFDRYFKY	65.13	94.35	9	ORF1ab	4673	4681	0.01	15	10	0.425	KLFDRYFKY	yes
KVNSTLEQY	63.62	66.94	9	ORF1ab	5668	5676	0.01	11	10	0.410		
KQFDTYNLW	52.54	52.54	9	ORF1ab	6437	6445	0.01	10	10	0.406	KQFDTYNLW	yes
KAYNVTQAF	38.52	65.34	9	N	266	274	0.01	13	9	0.380	KAYNVTQAF	yes
STNVTIATY	55.21	70.73	9	ORF1ab	2273	2281	0.01	13	9	0.380	STNVTIATY	
ATSRTLSYY	61.41	63.62	9	M	171	179	0.01	10	9	0.369	ATSRTLSYY	yes
VVIPDYNTY	46.79	67.9	9	ORF1ab	4072	4080	0.01	10	9	0.369		
TTNGDFLHF	36.85	63.07	9	ORF1ab	2875	2883	0.11	11	9	0.363		
VMYMGTLSY	61.92	66.42	9	ORF1ab	1768	1776	0.01	11	8	0.336	VMYMGTLSY	
TSNQVAVLY	43.81	59.81	9	S	604	612	0.02	11	8	0.335	TSNQVAVLY	
KSHKPPISF	45.3	54.27	9	ORF1ab	5397	5405	0.01	10	8	0.332		
QVVDMSMTY	52.7	57.96	9	ORF1ab	1582	1590	0.01	10	8	0.332	QVVDMSMTY	
TTLPVNVAF	36.85	54.21	9	ORF1ab	6499	6507	0.05	11	8	0.332	TTLPVNVAF	
RTIKVFTTV	53.21	58.55	9	ORF1ab	1566	1574	0.02	10	8	0.331	RTIKVFTTV	
IVSTIQRKY	45.21	57.3	9	ORF1ab	1398	1406	0.02	10	8	0.331		
RTAPHGHVM	38.92	56.36	9	ORF1ab	77	85	0.06	11	8	0.331		
SSLPSYAAF	50.01	52,91	9	ORF1ab	3949	3957	0.15	10	8	0.318		
LMDGSIIQF	77.79	79.22	9	ORF1ab	2956	2964	0.22	10	8	0.311		
EYADVFHLY	41.72	66.27	9	ORF1ab	5268	5276	0.05	12	7	0.299		
RLYYDSMSY	58.51	65.44	9	ORF1ab	4901	4909	0.01	10	7	0.295	RLYYDSMSY	yes
SFYEDFLEY	47.34	68.55	9	ORF8	103	111	0.01	10	7	0.295		
YVYSRVKNL	56.12	67.15	9	E	57	65	0.02	10	7	0.294	YVYSRVKNL	yes
TILDGISQY	51.35	66.15	9	ORF1ab	568	576	0.02	10	7	0.294		
YLITPVHVM	59.94	67.53	9	ORF1ab	2788	2796	0.06	11	7	0.294	YLITPVHVM	
LVKPSFYVY	33.02	49.24	9	E	51	59	0.03	10	7	0.293	LVKPSFYVY	
YLFDESGEF	57.54	73.42	9	ORF1ab	906	914	0.03	10	7	0.293	YLFDESGEF	
TIKPVTYKL	27.5	77.44	9	ORF1ab	1876	1884	0.08	11	7	0.292		
LVSDIDITF	33.01	54.11	9	ORF1ab	1270	1278	0.1	11	7	0.290	LVSDIDITF	
QSAPHGVVF	33.01	63.07	9	S	1054	1062	0.1	11	7	0.290	QSAPHGVVF	
HLDGEVITF	65	83.73	9	ORF1ab	1545	1553	0.25	12	7	0.279	HLDGEVITF	
HADQLTPTW	35.4	67.34	9	S	626	634	0.01	12	6	0.266		
GVYSVIYLY	46.26	67.94	9	ORF1ab	3103	3111	0.02	11	6	0.261		
KMKDLSPRW	40.88	52.54	9	N	100	108	0.01	10	6	0.258	KMKDLSPRW	yes
TLKEILVTY	40.09	65.09	9	ORF1ab	4533	4541	0.01	10	6	0.258	TLKEILVTY	
QTFSVLACY	40.49	58.62	9	ORF1ab	3373	3381	0.04	10	6	0.255		
QALLKTVQF	30.58	58.36	9	ORF1ab	4576	4584	0.08	10	6	0.251		
VARDLSLQF	28.77	52.42	9	ORF1ab	2469	2477	0.12	11	6	0.251	VARDLSLQF	
VAMPNLYKM	25.89	58.84	9	ORF1ab	6807	6815	0.16	11	6	0.247	VAMPNLYKM	
NVIPTITQM	26.94	63.45	9	ORF1ab	4923	4931	0.01	10	5	0.221	NVIPTITQM	
AMDEFIERY	34.76	73.12	9	ORF1ab	6669	6677	0.02	10	5	0.220		
SSVELKHFF	18.99	52.91	9	ORF1ab	4825	4833	0.06	10	5	0.216		

SLDNVLSTF	49.18	81.76	9	ORF1ab	2625	2633	0.21	11	5	0.205	SLDNVLSTF
HVISTSHKL	14.48	64.82	9	ORF1ab	5357	5365	0.04	10	4	0.181	
AQLPAPRTL	21.39	75.11	9	ORF1ab	5727	5735	0.09	11	4	0.180	
VYDPLQPEL	33.97	77.02	9	S	1134	1142	0.04	11	3	0.148	
STQDLFLPF	10.29	63.07	9	S	50	58	0.1	11	2	0.105	STQDLFLPF

Table S8: List of the selected CD8^{+} T cell epitopes

	Epitope	Coverage PR<0.5 %	Coverage PR <1 %	protein	Start	end	PR	Promiscuity PR<0.5 %	Promiscuity PR<1%	Final scores
1	*VVYRGTTTY	66.42	67.9	ORF1ab	5533	5541	0.01	11	12	0.451
2	*KLFDRYFKY	65.13	94.35	ORF1ab	4673	4681	0.01	10	15	0.425
3	KVNSTLEQY	63.62	66.94	ORF1ab	5668	5676	0.01	10	11	0.410
4	*KQFDTYNLW	52.54	52.54	ORF1ab	6437	6445	0.01	10	10	0.406
5	*STNVTIATY	55.21	70.73	ORF1ab	2273	2281	0.01	9	13	0.380
6	*KAYNVTQAF	38.52	65.34	N	266	274	0.01	9	13	0.380
7	*ATSRTLSYY	61.41	63.62	M	171	179	0.01	9	10	0.369
8	VVIPDYNTY	46.79	67.9	ORF1ab	4072	4080	0.01	9	10	0.369
9	TTNGDFLHF	36.85	63.07	ORF1ab	2875	2883	0.11	9	11	0.363
10	*VMYMGTLSY	61.92	66.42	ORF1ab	1768	1776	0.01	8	11	0.336
11	*TSNQVAVLY	43.81	59.81	S	604	612	0.02	8	11	0.335
12	*QVVDMSMTY	52.7	57.96	ORF1ab	1582	1590	0.01	8	10	0.332
13	KSHKPPISF	45.3	54.27	ORF1ab	5397	5405	0.01	8	10	0.332
14	*TTLPVNVAF	36.85	54.21	ORF1ab	6499	6507	0.05	8	11	0.332
15	*RTIKVFTTV	53.21	58.55	ORF1ab	1566	1574	0.02	8	10	0.331
16	IVSTIQRKY	45.21	57.3	ORF1ab	1398	1406	0.02	8	10	0.331
17	RTAPHGHVM	38.92	56.36	ORF1ab	77	85	0.06	8	11	0.331
18	SSLPSYAAF	50.01	52,91	ORF1ab	3949	3957	0.15	8	10	0.318
19	LMDGSIIQF	77.79	79.22	ORF1ab	2956	2964	0.22	8	10	0.311
20	EYADVFHLY	41.72	66.27	ORF1ab	5268	5276	0.05	7	12	0.299
21	*RLYYDSMSY	58.51	65.44	ORF1ab	4901	4909	0.01	7	10	0.295
22	SFYEDFLEY	47.34	68.55	ORF8	103	111	0.01	7	10	0.295
23	*YVYSRVKNL	56.12	67.15	E	57	65	0.02	7	10	0.294
24	TILDGISQY	51.35	66.15	ORF1ab	568	576	0.02	7	10	0.294
25	*YLITPVHVM	59.94	67.53	ORF1ab	2788	2796	0.06	7	11	0.294
26	*YLFDESGEF	57.54	73.42	ORF1ab	906	914	0.03	7	10	0.293
27	TIKPVTYKL	27.5	77.44	ORF1ab	1876	1884	0.08	7	11	0.292
28	*HLDGEVITF	65	83.73	ORF1ab	1545	1553	0.25	7	12	0.279
29	HADQLTPTW	35.4	67.34	S	626	634	0.01	6	12	0.266
30	GVYSVIYLY	46.26	67.94	ORF1ab	3103	3111	0.02	6	11	0.261
31	*KMKDLSPRW	40.88	52.54	N	100	108	0.01	6	10	0.258
32	*TLKEILVTY	40.09	65.09	ORF1ab	4533	4541	0.01	6	10	0.258
33	QTFSVLACY	40.49	58.62	ORF1ab	3373	3381	0.04	6	10	0.255
34	QALLKTVQF	30.58	58.36	ORF1ab	4576	4584	0.08	6	10	0.251
35	AMDEFIERY	34.76	73.12	ORF1ab	6669	6677	0.02	5	10	0.220
36	SSVELKHFF	18.99	52.91	ORF1ab	4825	4833	0.06	5	10	0.216
37	*SLDNVLSTF	49.18	81.76	ORF1ab	2625	2633	0.21	5	11	0.205
38	AQLPAPRTL	21.39	75.11	ORF1ab	5727	5735	0.09	4	11	0.180
39	VYDPLQPEL	33.97	77.02	S	1134	1142	0.04	3	11	0.148

Table S9. The full analysis results of CD4 epitope conservation among all genomes and among genomes within each WHO clade, with each value ranging from 0 to 1 following a computation using formula (1), in column F and (2) in columns G to T.

Sequence	Gene	Protein	Start	End	Conservancy	Omicron	Delta	Alpha	Beta	Mu	Epsilon	Gamma	Zeta	Lambda	Iota	Theta	Eta	Kappa	GH/490R
LFTRFFYVLGLAAIMQLF	ORF1ab	NSP3	1515	1533	0.994731545	0.99792746	0.99545879	0.97683154	0.99511668	0.99704524	0.99596673	0.99750334	0.99867198	0.99650485	0.99934417	0.99671053	0.98941239	0.99717753	1
TERLKLFAAETLKATEE	ORF1ab	NSP13	127	143	0.991291956	0.98344449	0.99721312	0.99866737	0.9994835	0.99833795	0.99817968	0.99862253	1	0.99941748	0.99774102	1	0.99761779	0.99407282	0.99837925
VTLVFLFVAAIFYLITPVH	ORF1ab	NSP4	13	33	0.989079139	0.99448791	0.99097002	0.95710867	0.99610274	0.99612188	0.99646643	0.97942405	0.99269588	0.99553398	0.99191139	0.99671053	0.99258867	0.98899238	0.9821718
MPNMLRIMASLVLARKI	ORF1ab	NSP12	626	643	0.996545262	0.99791691	0.99502872	0.99840909	0.99849744	0.89621422	0.99279009	0.99786923	0.99734396	0.99883495	0.99701232	0.99013158	0.99576496	0.98391194	0.99675851
LMWLSYFIASFRLFARTR	M	M	90	107	0.997255049	0.9985759	0.99642446	0.99456783	0.99892004	0.99852262	0.99753721	0.9978477	1	0.99747573	0.99919843	1	0.99655903	0.99943551	0.99837925
NSWLMWLIINLVQMAF	ORF1ab	NSP3	1543	1562	0.996257711	0.99801868	0.99399815	0.99795847	0.99746443	0.99741459	0.99396795	0.99638414	0.99734396	0.9976699	0.99963565	1	0.98941239	0.99181485	0.99837925
LMIERFVSLAIDAYPLT	ORF1ab	NSP12	854	870	0.998680407	0.99949117	0.99753698	0.99956312	0.99971827	0.99944598	0.99942892	0.99976325	1	0.99961165	0.99963565	1	0.99708841	0.99971775	1
HLVDFQVTIAEILLI	ORF6	NS6	3	17	0.995808758	0.99492662	0.99661458	0.9959664	0.99788703	0.99593721	0.99814398	0.99659937	0.99800797	0.98582524	0.99759528	1	0.99841186	0.99520181	1
VFLLVTLAILTALRLCAY	E	E	25	42	0.999186651	0.99948435	0.99900024	0.99873607	0.99826267	0.99815328	0.99907199	0.99922517	0.99933599	0.99941748	0.99905269	0.99671053	0.99841186	0.99887101	1
IILASFSASTSAFVET	ORF1ab	NSP2	295	310	0.996949871	0.99757873	0.99632415	0.99682917	0.99774616	0.99704524	0.99739444	0.99591064	0.99734396	0.99514563	0.99810537	0.99013158	0.99602965	0.99745978	0.99675851
FAWWTAFVTNVNASS	ORF1ab	NSP16	187	201	0.998370822	0.99897366	0.99764187	0.99854648	0.99868526	0.99981533	0.99932184	0.99892385	0.99667995	0.99902913	0.99897981	0.99671053	0.99841186	0.99830652	1
SLFFFLYENAFLPFAM	ORF1ab	NSP6	32	47	0.978245589	0.98832055	0.96578519	0.98464595	0.98741607	0.97396122	0.96723418	0.98687099	0.97609562	0.98271845	0.98848648	0.93092105	0.98650079	0.97516229	0.99513776
CTERLKLFAAETLKA	ORF1ab	NSP13	126	140	0.999980169	0.99999379	0.99999148	0.99987635	1	1	1	0.99997848	1	0.99980583	1	1	0.99973531	0.99971775	1
QLIKVTLVFLFVAAIFYL	ORF1ab	NSP4	9	26	0.999760925	0.99984983	0.99969712	0.99957686	1	0.99963066	0.99989292	0.99991391	1	0.99980583	1	1	1	1	1
FLCLFLLPSLATVAY	ORF1ab	NSP6	66	80	0.621890584	0.99741305	0.10642385	0.99555425	0.99643142	0.99759926	0.99671628	0.99472687	0.99933599	0.99805825	0.99788676	1	0.99497088	0.01552357	0.99837925
FLAFVVFLLVTLAILTAL	E	E	20	37	0.998055453	0.99918153	0.99860689	0.99851625	0.9994835	0.99926131	0.99846522	0.99845035	0.99933599	0.9984466	0.9987612	0.99671053	0.22816305	0.99915326	1

Table S10. The full analysis results of CD8 epitope conservation among all genomes and among genomes within each WHO clade, with each value ranging from 0 to 1 following a computation using formula (1) in column F, and (2) in columns G to T

Sequence	Gene	Protein	Start	End	Conservancy	Omicron	Delta	Alpha	Beta	Mu	Epsilon	Gamma	Zeta	Lambda	Iota	Theta	Eta	Kappa	GH/490R
VVYRGTTTY	ORF1ab	NSP13	209	217	0.99822099	0.99922869	0.99698301	0.99873057	0.99873222	0.99889197	0.999536	0.9986871	0.99933599	0.99572816	0.99934417	0.99671053	0.99814717	0.99887101	0.99837925
KLFDRYFKY	ORF1ab	NSP12	281	289	0.99905802	0.99917409	0.99885601	0.99936254	0.99934263	0.99926131	0.99921476	0.99907451	0.99933599	0.9961165	0.99963565	1	0.99920593	1	1
KVNSTLEQY	ORF1ab	NSP13	347	355	0.99514965	0.99350562	0.99588557	0.9986344	0.99793398	0.97820868	0.99828675	0.99776161	0.99734396	0.99805825	0.99919843	1	0.99629434	0.99491956	1
KQFDTYNLW	ORF1ab	NSP14	512	520	0.99891287	0.9993801	0.99833154	0.99932132	0.99849744	0.99907664	0.99942892	0.99821359	0.99601594	0.9992233	0.99963565	1	0.99973531	0.99887101	1
STNVTIATY	ORF1ab	NSP3	1455	1463	0.99573439	0.99697558	0.99477043	0.99409248	0.99896699	0.99741459	0.98954206	0.99817055	0.99734396	0.99728155	0.99861546	0.99013158	0.99364743	0.99774203	1
KAYNVTQAF	N	N	266	274	0.99570905	0.99799075	0.99360611	0.99439473	0.9976053	0.99815328	0.99489596	0.99513581	0.99800797	0.99514563	0.99795963	1	0.98941239	0.99548405	0.99027553
ATSRTLSYY	M	M	171	179	0.99935136	0.99914864	0.99950897	0.99949168	0.99953045	0.99963066	0.99960738	0.99961259	1	1	0.99948991	1	1	0.99971775	1
VVIPDYNTY	ORF1ab	NSP8	130	138	0.99864047	0.99948062	0.9975927	0.99939001	0.99915481	0.99963066	0.99835814	0.99931127	0.95949535	0.99805825	0.99883407	0.99671053	0.99947062	0.9957663	0.99675851
TTNGDFLHF	ORF1ab	NSP4	113	121	0.9984069	0.99941361	0.99795196	0.99890367	0.99962436	0.99926131	0.95666917	0.99866558	1	0.99883495	0.99978139	1	0.99602965	0.99887101	1
VMYMGTLSY	ORF1ab	NSP3	950	958	0.99851074	0.99915485	0.99819322	0.99871134	0.9976053	0.99926131	0.99889353	0.9950282	0.99734396	0.99786408	0.96691685	0.92434211	1	0.99689529	1
TSNQVAVLY	S	Spike	604	612	0.99935081	0.99967919	0.99898582	0.99956037	0.99985914	1	0.99975015	0.99780466	1	0.99883495	0.99978139	1	0.99788248	0.99887101	1
QVVDMSMTY	ORF1ab	NSP3	764	772	0.99583961	0.99675964	0.99633923	0.98926755	0.99877917	0.99722992	0.99535996	0.99698678	0.99667995	0.99708738	0.99657509	0.99013158	0.99841186	0.99830652	1
KSHKPPISF	ORF1ab	NSP13	73	81	0.57948707	0.99785486	0.00620639	0.98070577	0.99840353	0.99722992	0.99707321	0.99711592	0.99667995	0.99339806	0.99759528	0.99671053	0.99602965	0.99604855	1
TTLPVNVAF	ORF1ab	NSP15	47	55	0.99915112	0.99966802	0.99882585	0.99838161	0.9943654	1	0.99964307	0.99950497	0.99933599	0.99961165	0.99978139	1	0.99973531	0.99802427	1
RTIKVFTTV	ORF1ab	NSP3	748	756	0.99592912	0.99919146	0.99755993	0.98317044	0.99976523	0.99907664	0.99942892	0.99769704	0.99667995	0.99902913	0.99883407	0.99342105	0.99550026	0.02568445	1
IVSTIQRKY	ORF1ab	NSP3	580	588	0.99830637	0.99821786	0.9985846	0.99758753	0.99521059	0.99889197	0.9995003	0.99799836	1	0.99941748	0.99941704	1	0.99682372	0.99971775	0.99837925
RTAPHGHVM	ORF1ab	NSP1	77	85	0.96580352	0.95770712	0.96744447	0.99093539	0.99074987	0.92373038	0.96798372	0.97535621	0.99667995	0.99009709	0.97894046	0.98684211	0.99232398	0.99802427	0.99351702
SSLPSYAAF	ORF1ab	NSP8	7	15	0.99818215	0.99898731	0.99776053	0.99718912	0.99483495	0.99907664	0.99792983	0.99875167	0.99800797	0.98990291	0.98506158	0.96710526	0.99682372	0.99943551	0.97730956
LMDGSIIQF	ORF1ab	NSP4	193	201	0.99916599	0.99969222	0.99868294	0.99925813	0.99976523	0.97506925	0.9995003	0.99905299	0.99933599	0.99805825	0.99810537	0.99342105	0.99682372	0.99887101	1
EYADVFHLY	ORF1ab	NSP12	876	884	0.99659346	0.9993801	0.99290202	0.99893115	0.99769921	0.99593721	0.99964307	0.99948345	1	0.9961165	0.99868833	1	0.99841186	0.99915326	0.99837925
RLYYDSMSY	ORF1ab	NSP12	513	521	0.9985774	0.99874717	0.99890256	0.9994642	0.99953045	0.77820868	0.99935753	0.99950497	0.99933599	0.99980583	0.99963565	1	0.99788248	0.99830652	0.99351702
SFYEDFLEY	ORF8	NS8	103	111	0.99525156	0.99873786	0.99229167	0.99290273	0.97793116	0.98153278	0.9951815	0.99883776	0.99867198	0.99184466	0.99861546	1	0.99258867	0.99604855	0.99837925
YVYSRVKNL	E	E	57	65	0.99566856	0.99785486	0.99275976	0.99740893	0.99849744	0.99796861	0.99878645	0.9975679	0.99468792	0.99165049	0.99890694	0.99013158	0.99761779	0.99604855	1
TILDGISQY	ORF1ab	NSP2	388	396	0.99766627	0.99839843	0.99718624	0.99684016	0.99896699	0.99833795	0.99286148	0.99651328	0.99933599	0.9961165	0.99905269	0.99671053	0.99629434	0.99802427	0.99837925
YLITPVHVM	ORF1ab	NSP4	25	33	0.99710218	0.99722255	0.99680273	0.99768096	0.99845049	0.99870729	0.99778706	0.99799836	0.9940239	0.99825243	0.99351454	1	0.99602965	0.99802427	1
YLFDESGEF	ORF1ab	NSP3	88	96	0.99730711	0.99905495	0.99799458	0.9870117	0.98873081	0.99963066	0.99453903	0.99935431	0.99667995	0.99941748	0.9980325	1	0.99920593	0.99858877	1
TIKPVTYKL	ORF1ab	NSP3	1058	1066	0.99833722	0.99889237	0.99806407	0.99695282	0.9990609	0.99833795	0.9985366	0.99847187	0.99933599	0.99786408	0.9992713	1	0.99814717	0.99435507	1
HLDGEVITF	ORF1ab	NSP3	727	735	0.99735971	0.99853061	0.99592032	0.99783483	0.9985444	0.99907664	0.99839383	0.99804141	0.99734396	0.99728155	0.99934417	1	0.99920593	0.99858877	1
HADQLTPTW	S	Spike	625	633	0.9979574	0.99757873	0.99840563	0.997626	0.99981218	0.99722992	0.99960738	0.99767552	0.9873838	0.9961165	0.99766815	1	0.99841186	0.99915326	1
GVYSVIYLY	ORF1ab	NSP4	340	348	0.99942325	0.99966554	0.99912349	0.99953564	0.99981218	1	0.99967877	0.9994404	0.99933599	0.99902913	0.99992713	1	0.99947062	0.99971775	1
KMKDLSPRW	N	N	100	108	0.99919794	0.99930005	0.99904678	0.99954938	0.9985444	0.99963066	0.99760859	0.99918213	1	0.99805825	0.99941704	1	0.99761779	0.99971775	1
TLKEILVTY	ORF1ab	NSP12	141	149	0.99883465	0.99913065	0.99858329	0.99860143	0.99920177	0.99778393	0.99878645	0.99857948	0.99933599	0.99941748	0.99905269	1	0.99682372	0.99943551	1
QTFSVLACY	ORF1ab	NSP5	110	118	0.99963836	0.99973442	0.99957256	0.99946145	0.99995305	0.99981533	0.999536	0.99954802	0.99933599	1	1	1	1	1	1
QALLKTVQF	ORF1ab	NSP12	184	192	0.97209135	0.99872421	0.93541931	0.99863165	0.99882613	0.99926131	0.99678766	0.99857948	0.99734396	0.99708738	0.99693944	1	0.99655903	0.99858877	1
AMDEFIERY	ORF1ab	NSP15	217	225	0.99610677	0.9990444	0.99529489	0.98564885	0.9994835	0.99778393	0.99832245	0.99866558	0.99667995	0.99631068	0.99861546	0.99342105	0.99867655	0.99661304	0.99837925
SSVELKHFF	ORF1ab	NSP12	433	441	0.99926515	0.99964382	0.99892485	0.99901633	0.99962436	0.99722992	0.9985366	0.99961259	0.99933599	1	0.99941704	1	0.99894124	0.99971775	1
SLDNVLSTF	ORF1ab	NSP3	1807	1815	0.99815984	0.99872607	0.99801555	0.9978101	0.99882613	0.99759926	0.99575258	0.99429641	0.99933599	0.99805825	0.99752241	0.01644737	0.9917946	0.99802427	1
AQLPAPRTL	ORF1ab	NSP13	403	411	0.99962541	0.99958301	0.99967221	0.9997005	0.99976523	0.99889197	0.99960738	0.99959106	1	1	0.99985426	1	0.99047115	1	1
VYDPLQPEL	S	Spike	1137	1145	0.99835347	0.99841767	0.99791066	0.99961532	0.99924872	0.99963066	0.99982154	0.99920365	1	0.99980583	0.99941704	1	0.99973531	0.99915326	0.95461912

Table S11. The full analysis results of CD4 epitope conservation among all genomes (column T) and among genomes within each WHO clade (columns F to S), but with each conservation result denoted with the raw fraction (total conserved genomes/total genomes)

Sequence	Gene	Protein	Start	End	Omicron	Delta	Alpha	Beta	Mu	Epsilon	Gamma	Zeta	Lambda	Iota	Theta	Eta	Kappa	GH/490R	Conservancy
LFTRFFYVLGLAAIMQLFF	ORF1ab	NSP3	1515	1533	1608208/1611548	1518436/1525363	355511/363943	21193/21297	5399/5415	27904/28017	46346/46462	1504/1506	5132/5150	13714/13723	303/304	3738/3778	3533/3543	617/617	3611538/3630666
TERLKLFAAETLKATEE	ORF1ab	NSP13	127	143	1584868/1611548	1521112/1525363	363458/363943	21286/21297	5406/5415	27966/28017	46398/46462	1506/1506	5147/5150	13692/13723	304/304	3769/3778	3522/3543	616/617	3599050/3630666
VTLVFLFVAAIFYLITPVHVM	ORF1ab	NSP4	13	33	1602665/1611548	1511589/1525363	348333/363943	21214/21297	5394/5415	27918/28017	45506/46462	1495/1506	5127/5150	13612/13723	303/304	3750/3778	3504/3543	606/617	3591016/3630666
MPNMLRIMASLVLARKHT	ORF1ab	NSP12	626	643	1608191/1611548	1517780/1525363	363364/363943	21265/21297	4853/5415	27815/28017	46363/46462	1502/1506	5144/5150	13682/13723	301/304	3762/3778	3486/3543	615/617	3618123/3630666
LMWLSYFIASFRLFARTR	M	M	90	107	1609253/1611548	1519909/1525363	361966/363943	21274/21297	5407/5415	27948/28017	46362/46462	1506/1506	5137/5150	13712/13723	304/304	3765/3778	3541/3543	616/617	3620700/3630666
NSWLMWLIINLVQMAPISAM	ORF1ab	NSP3	1543	1562	1608355/1611548	1516208/1525363	363200/363943	21243/21297	5401/5415	27848/28017	46294/46462	1502/1506	5138/5150	13718/13723	304/304	3738/3778	3514/3543	616/617	3617079/3630666
LMIERFVSLAIDAYPLT	ORF1ab	NSP12	854	870	1610728/1611548	1521606/1525363	363784/363943	21291/21297	5412/5415	28001/28017	46451/46462	1506/1506	5148/5150	13718/13723	304/304	3767/3778	3542/3543	617/617	3625875/3630666
HLVDFQVTIAEILLI	ORF6	NS6	3	17	1603372/1611548	1520199/1525363	362475/363943	21252/21297	5393/5415	27965/28017	46304/46462	1503/1506	5077/5150	13690/13723	304/304	3772/3778	3526/3543	617/617	3615449/3630666
VFLLVTLAILTALRLCAY	E	E	25	42	1610717/1611548	1523838/1525363	363483/363943	21260/21297	5405/5415	27991/28017	46426/46462	1505/1506	5147/5150	13710/13723	303/304	3772/3778	3539/3543	617/617	3627713/3630666
IILASFSASTSAFVET	ORF1ab	NSP2	295	310	1607646/1611548	1519756/1525363	362789/363943	21249/21297	5399/5415	27944/28017	46272/46462	1502/1506	5125/5150	13697/13723	301/304	3763/3778	3534/3543	615/617	3619592/3630666
FAWWTAFVTNVNASS	ORF1ab	NSP16	187	201	1609894/1611548	1521766/1525363	363414/363943	21269/21297	5414/5415	27998/28017	46412/46462	1501/1506	5145/5150	13709/13723	303/304	3772/3778	3537/3543	617/617	3624751/3630666
SLFFFLYENAFLPFAM	ORF1ab	NSP6	32	47	1592726/1611548	1473173/1525363	358355/363943	21029/21297	5274/5415	27099/28017	45852/46462	1470/1506	5061/5150	13565/13723	283/304	3727/3778	3455/3543	614/617	3551683/3630666
CTERLKLFAAETLKA	ORF1ab	NSP13	126	140	1611538/1611548	1525350/1525363	363898/363943	21297/21297	5415/5415	28017/28017	46461/46462	1506/1506	5149/5150	13723/13723	304/304	3777/3778	3542/3543	617/617	3630594/3630666
QLIKVTLVFLFVAAIFYL	ORF1ab	NSP4	9	26	1611306/1611548	1524901/1525363	363789/363943	21297/21297	5413/5415	28014/28017	46458/46462	1506/1506	5149/5150	13723/13723	304/304	3778/3778	3543/3543	617/617	3629798/3630666
FLCLFLLPSLATVAY	ORF1ab	NSP6	66	80	1607379/1611548	162335/1525363	362325/363943	21221/21297	5402/5415	27925/28017	46217/46462	1505/1506	5140/5150	13694/13723	304/304	3759/3778	55/3543	616/617	2257877/3630666
FLAFVVFLLVTLAILTAL	E	E	20	37	1610229/1611548	1523238/1525363	363403/363943	21286/21297	5411/5415	27974/28017	46390/46462	1505/1506	5142/5150	13706/13723	303/304	862/3778	3540/3543	617/617	3623606/3630666

Table S12. The full analysis results of CD8 epitope conservation among all genomes (columns T) and among genomes within each WHO clade (columns F to S), but with each conservation result denoted with the raw fraction (total conserved genomes/total genomes).

Sequence	Gene	Protein	Start	End	Omicron	Delta	Alpha	Beta	Mu	Epsilon	Gamma	Zeta	Lambda	Iota	Theta	Eta	Kappa	GH/490R	Conservancy
VVYRGTTTY	ORF1ab	NSP13	209	217	1610305/1611548	1520761/1525363	363481/363943	21270/21297	5409/5415	28004/28017	46401/46462	1505/1506	5128/5150	13714/13723	303/304	3771/3778	3539/3543	616/617	3624207/3630666
KLFDRYFKY	ORF1ab	NSP12	281	289	1610217/1611548	1523618/1525363	363711/363943	21283/21297	5411/5415	27995/28017	46419/46462	1505/1506	5130/5150	13718/13723	304/304	3775/3778	3543/3543	617/617	3627246/3630666
KVNSTLEQY	ORF1ab	NSP13	347	355	1601082/1611548	1519087/1525363	363446/363943	21253/21297	5297/5415	27969/28017	46358/46462	1502/1506	5140/5150	13712/13723	304/304	3764/3778	3525/3543	617/617	3613056/3630666
KQFDTYNLW	ORF1ab	NSP14	512	520	1610549/1611548	1522818/1525363	363696/363943	21265/21297	5410/5415	28001/28017	46379/46462	1500/1506	5146/5150	13718/13723	304/304	3777/3778	3539/3543	617/617	3626719/3630666
STNVTIATY	ORF1ab	NSP3	1455	###	1606674/1611548	1517386/1525363	361793/363943	21275/21297	5401/5415	27724/28017	46377/46462	1502/1506	5136/5150	13704/13723	301/304	3754/3778	3535/3543	617/617	3615179/3630666
KAYNVTQAF	N	N	266	274	1608310/1611548	1515610/1525363	361903/363943	21246/21297	5405/5415	27874/28017	46236/46462	1503/1506	5125/5150	13695/13723	304/304	3738/3778	3527/3543	611/617	3615087/3630666
ATSRTLSYY	M	M	171	179	1610176/1611548	1524614/1525363	363758/363943	21287/21297	5413/5415	28006/28017	46444/46462	1506/1506	5150/5150	13716/13723	304/304	3778/3778	3542/3543	617/617	3628311/3630666
VVIPDYNTY	ORF1ab	NSP8	130	138	1610711/1611548	1521691/1525363	363721/363943	21279/21297	5413/5415	27971/28017	46430/46462	1445/1506	5140/5150	13707/13723	303/304	3776/3778	3528/3543	615/617	3625730/3630666
TTNGDFLHF	ORF1ab	NSP4	113	121	1610603/1611548	1522239/1525363	363544/363943	21289/21297	5411/5415	26803/28017	46400/46462	1506/1506	5144/5150	13720/13723	304/304	3763/3778	3539/3543	617/617	3624882/3630666
VMYMGTLSY	ORF1ab	NSP3	950	958	1610186/1611548	1522607/1525363	363474/363943	21246/21297	5411/5415	27986/28017	46231/46462	1502/1506	5139/5150	13269/13723	281/304	3778/3778	3532/3543	617/617	3625259/3630666
TSNQVAVLY	S	Spike	604	612	1611031/1611548	1523816/1525363	363783/363943	21294/21297	5415/5415	28010/28017	46360/46462	1506/1506	5144/5150	13720/13723	304/304	3770/3778	3539/3543	617/617	3628309/3630666
QVVDMSMTY	ORF1ab	NSP3	764	772	1606326/1611548	1519779/1525363	360037/363943	21271/21297	5400/5415	27887/28017	46322/46462	1501/1506	5135/5150	13676/13723	301/304	3772/3778	3537/3543	617/617	3615561/3630666
KSHKPPISF	ORF1ab	NSP13	73	81	1608091/1611548	9467/1525363	356921/363943	21263/21297	5400/5415	27935/28017	46328/46462	1501/1506	5116/5150	13690/13723	303/304	3763/3778	3529/3543	617/617	2103924/3630666
TTLPVNVAF	ORF1ab	NSP15	47	55	1611013/1611548	1523572/1525363	363354/363943	21177/21297	5415/5415	28007/28017	46439/46462	1505/1506	5148/5150	13720/13723	304/304	3777/3778	3536/3543	617/617	3627584/3630666
RTIKVFTTV	ORF1ab	NSP3	748	756	1610245/1611548	1521641/1525363	357818/363943	21292/21297	5410/5415	28001/28017	46355/46462	1501/1506	5145/5150	13707/13723	302/304	3761/3778	91/3543	617/617	3615886/3630666
IVSTIQRKY	ORF1ab	NSP3	580	588	1608676/1611548	1523204/1525363	363065/363943	21195/21297	5409/5415	28003/28017	46369/46462	1506/1506	5147/5150	13715/13723	304/304	3766/3778	3542/3543	616/617	3624517/3630666
RTAPHGHVM	ORF1ab	NSP1	77	85	1543391/1611548	1475704/1525363	360644/363943	21100/21297	5002/5415	27120/28017	45317/46462	1501/1506	5099/5150	13434/13723	300/304	3749/3778	3536/3543	613/617	3506510/3630666
SSLPSYAAF	ORF1ab	NSP8	7	15	1609916/1611548	1521947/1525363	362920/363943	21187/21297	5410/5415	27959/28017	46404/46462	1503/1506	5098/5150	13518/13723	294/304	3766/3778	3541/3543	603/617	3624066/3630666
LMDGSIIQF	ORF1ab	NSP4	193	201	1611052/1611548	1523354/1525363	363673/363943	21292/21297	5280/5415	28003/28017	46418/46462	1505/1506	5140/5150	13697/13723	302/304	3766/3778	3539/3543	617/617	3627638/3630666
EYADVFHLY	ORF1ab	NSP12	876	884	1610549/1611548	1514536/1525363	363554/363943	21248/21297	5393/5415	28007/28017	46438/46462	1506/1506	5130/5150	13705/13723	304/304	3772/3778	3540/3543	616/617	3618298/3630666
RLYYDSMSY	ORF1ab	NSP12	513	521	1609529/1611548	1523689/1525363	363748/363943	21287/21297	4214/5415	27999/28017	46439/46462	1505/1506	5149/5150	13718/13723	304/304	3770/3778	3537/3543	613/617	3625501/3630666
SFYEDFLEY	ORF8	NS8	103	111	1609514/1611548	1513605/1525363	361360/363943	20827/21297	5315/5415	27882/28017	46408/46462	1504/1506	5108/5150	13704/13723	304/304	3750/3778	3529/3543	616/617	3613426/3630666
YVYSRVKNL	E	E	57	65	1608091/1611548	1514319/1525363	363000/363943	21265/21297	5404/5415	27983/28017	46349/46462	1498/1506	5107/5150	13708/13723	301/304	3769/3778	3529/3543	617/617	3614940/3630666
TILDGISQY	ORF1ab	NSP2	388	396	1608967/1611548	1521071/1525363	362793/363943	21275/21297	5406/5415	27817/28017	46300/46462	1505/1506	5130/5150	13710/13723	303/304	3764/3778	3536/3543	616/617	3622193/3630666
YLITPVHVM	ORF1ab	NSP4	25	33	1607072/1611548	1520486/1525363	363099/363943	21264/21297	5408/5415	27955/28017	46369/46462	1497/1506	5141/5150	13634/13723	304/304	3763/3778	3536/3543	617/617	3620145/3630666
YLFDESGEF	ORF1ab	NSP3	88	96	1610025/1611548	1522304/1525363	359216/363943	21057/21297	5413/5415	27864/28017	46432/46462	1501/1506	5147/5150	13696/13723	304/304	3775/3778	3538/3543	617/617	3620889/3630666
TIKPVTYKL	ORF1ab	NSP3	1058	###	1609763/1611548	1522410/1525363	362834/363943	21277/21297	5406/5415	27976/28017	46391/46462	1505/1506	5139/5150	13713/13723	304/304	3771/3778	3523/3543	617/617	3624629/3630666
HLDGEVITF	ORF1ab	NSP3	727	735	1609180/1611548	1519140/1525363	363155/363943	21266/21297	5410/5415	27972/28017	46371/46462	1502/1506	5136/5150	13714/13723	304/304	3775/3778	3538/3543	617/617	3621080/3630666
HADQLTPTW	S	Spike	625	633	1607646/1611548	1522931/1525363	363079/363943	21293/21297	5400/5415	28006/28017	46354/46462	1487/1506	5130/5150	13691/13723	304/304	3772/3778	3540/3543	617/617	3623250/3630666
GVYSVIYLY	ORF1ab	NSP4	340	348	1611009/1611548	1524026/1525363	363774/363943	21293/21297	5415/5415	28008/28017	46436/46462	1505/1506	5145/5150	13722/13723	304/304	3776/3778	3542/3543	617/617	3628572/3630666
KMKDLSPRW	N	N	100	108	1610420/1611548	1523909/1525363	363779/363943	21266/21297	5413/5415	27950/28017	46424/46462	1506/1506	5140/5150	13715/13723	304/304	3769/3778	3542/3543	617/617	3627754/3630666
TLKEILVTY	ORF1ab	NSP12	141	149	1610147/1611548	1523202/1525363	363434/363943	21280/21297	5403/5415	27983/28017	46396/46462	1505/1506	5147/5150	13710/13723	304/304	3766/3778	3541/3543	617/617	3626435/3630666
QTFSVLACY	ORF1ab	NSP5	110	118	1611120/1611548	1524711/1525363	363747/363943	21296/21297	5414/5415	28004/28017	46441/46462	1505/1506	5150/5150	13723/13723	304/304	3778/3778	3543/3543	617/617	3629353/3630666
QALLKTVQF	ORF1ab	NSP12	184	192	1609492/1611548	1426854/1525363	363445/363943	21272/21297	5411/5415	27927/28017	46396/46462	1502/1506	5135/5150	13681/13723	304/304	3765/3778	3538/3543	617/617	3529339/3630666
AMDEFIERY	ORF1ab	NSP15	217	225	1610008/1611548	1518186/1525363	358720/363943	21286/21297	5403/5415	27970/28017	46400/46462	1501/1506	5131/5150	13704/13723	302/304	3773/3778	3531/3543	616/617	3616531/3630666
SSVELKHFF	ORF1ab	NSP12	433	441	1610974/1611548	1523723/1525363	363585/363943	21289/21297	5400/5415	27976/28017	46444/46462	1505/1506	5150/5150	13715/13723	304/304	3774/3778	3542/3543	617/617	3627998/3630666
SLDNVLSTF	ORF1ab	NSP3	1807	###	1609495/1611548	1522336/1525363	363146/363943	21272/21297	5402/5415	27898/28017	46197/46462	1505/1506	5140/5150	13689/13723	5/304	3747/3778	3536/3543	617/617	3623985/3630666
AQLPAPRTL	ORF1ab	NSP13	403	411	1610876/1611548	1524863/1525363	363834/363943	21292/21297	5409/5415	28006/28017	46443/46462	1506/1506	5150/5150	13721/13723	304/304	3742/3778	3543/3543	617/617	3629306/3630666
VYDPLQPEL	S	Spike	1137	###	1608998/1611548	1522176/1525363	363803/363943	21281/21297	5413/5415	28012/28017	46425/46462	1506/1506	5149/5150	13715/13723	304/304	3777/3778	3540/3543	589/617	3624688/3630666

Table S13. Multiepitope -TLR4 cluspro 753719

Cluster	Members	Representative	Weighted Score
0	39	Center	-1091.6
0	39	Lowest Energy	-1236.5
1	33	Center	-1292.6
1	33	Lowest Energy	-1435.2
2	33	Center	-1231.4
2	33	Lowest Energy	-1526.2
3	31	Center	-1240.6
3	31	Lowest Energy	-1415.4
4	27	Center	-1352.4
4	27	Lowest Energy	-1352.4
5	24	Center	-1099.1
5	24	Lowest Energy	-1276.8
6	23	Center	-1230.5
6	23	Lowest Energy	-1334.1
7	23	Center	-1058.3
7	23	Lowest Energy	-1168.1
8	22	Center	-1164.9
8	22	Lowest Energy	-1195.2
9	20	Center	-1057.2
9	20	Lowest Energy	-1215.5
10	19	Center	-1060.6
10	19	Lowest Energy	-1399.2
11	18	Center	-1063.1
11	18	Lowest Energy	-1155.2
12	15	Center	-1047.9
12	15	Lowest Energy	-1119.6
13	14	Center	-1107
13	14	Lowest Energy	-1196.9
14	14	Center	-1064.3
14	14	Lowest Energy	-1184.4
15	14	Center	-1192
15	14	Lowest Energy	-1192
16	14	Center	-1060.1
16	14	Lowest Energy	-1170.7
17	14	Center	-1025.1
17	14	Lowest Energy	-1165.6
18	13	Center	-1019.4
18	13	Lowest Energy	-1213.7
19	13	Center	-1083
19	13	Lowest Energy	-1141.4
20	13	Center	-1066.4
20	13	Lowest Energy	-1072.8
21	13	Center	-1062.9
21	13	Lowest Energy	-1190.7
22	12	Center	-1085.7
22	12	Lowest Energy	-1226.9

23	12	Center	-1036.3
23	12	Lowest Energy	-1327.1
24	12	Center	-1000.2
24	12	Lowest Energy	-1176.1
25	12	Center	-1182.9
25	12	Lowest Energy	-1224
26	12	Center	-1131.9
26	12	Lowest Energy	-1131.9
27	12	Center	-1007.6
27	12	Lowest Energy	-1215
28	11	Center	-1016
28	11	Lowest Energy	-1103.8
29	11	Center	-1052.6
29	11	Lowest Energy	-1064.1

Table S14. Accession code numbers for each protein of each SARS-CoV-2 strain

SARS-CoV-2 strains	Isolated from	NCBI protein accession number
EWuhan	Wuhan	YP_009724392.1
MWuhan	Wuhan	YP_009724393.1
NWuhan	Wuhan	YP_009724397.2
ORF10Wuhan	Wuhan	YP_009725255.1
ORF1abWuhan	Wuhan	YP_009724389.1
ORF1aWuhan	Wuhan	YP_009725295.1
ORF3aWuhan	Wuhan	YP_009724391.1
ORF6Wuhan	Wuhan	YP_009724394.1
ORF7aWuhan	Wuhan	YP_009724395.1
ORF7bWuhan	Wuhan	YP_009725318.1
ORF8	Wuhan	YP_009724396.1
SWuhan	Wuhan	YP_009724390.1
EAlfaEngland	Alfa	QOS14147.1
MAlfaEngland	Alfa	QOS14148.1
NAlfaEngland	Alfa	QOS14153.1
ORF10AlfaEngland	Alfa	QOS14154.1
ORF1aAlfaEngland	Alfa	QOS14144.1
ORF1abAlfaEngland	Alfa	QOS14143.1
ORF3aAlfaEngland	Alfa	QOS14146.1
ORF6AlfaEngland	Alfa	QOS14149.1
ORF7aAlfaEngland	Alfa	QOS14150.1
ORF7bAlfaEngland	Alfa	QOS14151.1
ORF8AlfaEngland	Alfa	QOS14152.1
SAlfaEngland	Alfa	QOS14145.1
EBetaSouthAfrica	Beta	QWP89167.1
MBetaSouthAfrica	Beta	QWP89168.1
NBetaSouthAfrica	Beta	QWP89173.1
ORF10BetaSouthAfrica	Beta	QWP89174.1
ORF1abBetaSouthAfrica	Beta	QWP89163.1
ORF1aBetaSouthAfrica	Beta	QWP89164.1
ORF3aBetaSouthAfrica	Beta	QWP89166.1
ORF6BetaSouthAfrica	Beta	QWP89169.1
ORF7aBetaSouthAfrica	Beta	QWP89170.1
ORF7bBetaSouthAfrica	Beta	QWP89171.1
ORF8BetaSouthAfrica	Beta	QWP89172.1
SBetaSouthAfrica	Beta	QWP89165.1
EDeltaIndia	Delta	UDF38170.1
MDeltaIndia	Delta	UDF38171.1
NDeltaIndia	Delta	UDF38176.1
ORF10DeltaIndia	Delta	UDF38177.1
ORF1abDeltaIndia	Delta	UDF38166.1

ORF1aDeltaIndia	Delta	UDF38167.1
ORF3aDeltaIndia	Delta	UDF38169.1
ORF6DeltaIndia	Delta	UDF38172.1
ORF7aDeltaIndia	Delta	UDF38173.1
ORF7bDeltaIndia	Delta	UDF38174.1
ORF8DeltaIndia	Delta	UDF38175.1
SDeltaIndia	Delta	UDF38168.1
EGamaBrazil	Gama	QVQ47341.1
MGamaBrazil	Gama	QVQ47342.1
NGamaBrazil	Gama	QVQ47347.1
ORF10GamaBrazil	Gama	QVQ47348.1
ORF1abGamaBrazil	Gama	QVQ47337.1
ORF1aGamaBrazil	Gama	QVQ47338.1
ORF3aGamaBrazil	Gama	QVQ47340.1
ORF6GamaBrazil	Gama	QVQ47343.1
ORF7aGamaBrazil	Gama	QVQ47344.1
ORF7bGamaBrazil	Gama	QVQ47345.1
ORF8GamaBrazil	Gama	QVQ47346.1
SGamaBrazil	Gama	QVQ47339.1
ELambdaPeru	Lambda	UDA75460.1
MLambdaPeru	Lambda	UDA75461.1
NLambdaPeru	Lambda	UDA75466.1
ORF10LambdaPeru	Lambda	UDA75467.1
ORF1abLambdaPeru	Lambda	UDA75456.1
ORF1aLambdaPeru	Lambda	UDA75457.1
ORF3aLambdaPeru	Lambda	UDA75459.1
ORF6LambdaPeru	Lambda	UDA75462.1
ORF7aLambdaPeru	Lambda	UDA75463.1
ORF7aLambdaPeru	Lambda	UDF43259.1
ORF7bLambdaPeru	Lambda	UDA75464.1
ORF8LambdaPeru	Lambda	UDA75465.1
SLambdaPeru	Lambda	UDA75458.1
EMuColombia	Mu	QYK89876.1
MMuColombia	Mu	QYK89877.1
NMuColombia	Mu	QYK89882.1
ORF10	Mu	QYK89883.1
ORF1abMuColombia	Mu	QYK89873.1
ORF1aMuColombia	Mu	QYK89874.1
ORF6MuColombia	Mu	QYK89878.1
ORF7aMuColombia	Mu	QYK89879.1
ORF7bMuColombia	Mu	QYK89880.1
ORF8MuColombia	Mu	QYK89881.1
SMuColombia	Mu	QYK89875.1
EOmicronSouthAfrica	Omicron	UFO69281.1

MOmicronSouthAfrica	Omicron	UFO69282.1
NOmicronSouthAfrica	Omicron	UFO69287.1
ORF10OmicronSouthAfrica	Omicron	UFO69288.1
ORF1abOmicronSouthAfrica	Omicron	UFO69277.1
ORF1aOmicronSouthAfrica	Omicron	UFO69278.1
ORF3aOmicronSouthAfrica	Omicron	UFO69280.1
ORF6OmicronSouthAfrica	Omicron	UFO69283.1
ORF7aOmicronSouthAfrica	Omicron	UFO69284.1
ORF7bOmicronSouthAfrica	Omicron	UFO69285.1
ORF8OmicronSouthAfrica	Omicron	UFO69286.1
SOmicronSouthAfrica	Omicron	UFO69279.1
ER1California	R1	UBZ78999.1
MR1California	R1	UBZ79000.1
NR1California	R1	UBZ79005.1
ORF10R1California	R1	UBZ79006.1
ORF1abR1California	R1	UBZ78995.1
ORF1aR1California	R1	UBZ78996.1
ORF3aR1California	R1	UBZ78998.1
ORF6R1California	R1	UBZ79001.1
ORF7aR1California	R1	UBZ79002.1
ORF7bR1California	R1	UBZ79003.1
ORF8R1California	R1	UBZ79004.1
SR1	R1	UBZ78997.1
EZetaPeru	Zeta	QZB58884.1
MZetaPeru	Zeta	QZB58885.1
NZetaPeru	Zeta	QZB58890.1
ORF10ZetaPeru	Zeta	QZB58891.1
ORF1abZetaPeru	Zeta	QZB58880.1
ORF1aZetaPeru	Zeta	QZB58881.1
ORF3aZetaPeru	Zeta	QZB58883.1
ORF6ZetaPeru	Zeta	QZB58886.1
ORF7bZetaPeru	Zeta	QZB58888.1
ORF8ZetaPeru	Zeta	QZB58889.1
Szeta	Zeta	QZB58882.1

Table S15. A.Full HLA Class II reference list of the IEDB platform (86), which contains 27 DR, DQ and DB alleles

HLA-DRB1*01:01
HLA-DRB1*03:01
HLA-DRB1*04:01
HLA-DRB1*04:05
HLA-DRB1*07:01
HLA-DRB1*08:02
HLA-DRB1*09:01
HLA-DRB1*11:01
HLA-DRB1*12:01
HLA-DRB1*13:02
HLA-DRB1*15:01
HLA-DRB3*01:01
HLA-DRB3*02:02
HLA-DRB4*01:01
HLA-DRB5*01:01
HLA-DQA1*05:01/DQB1*02:01
HLA-DQA1*05:01/DQB1*03:01
HLA-DQA1*03:01/DQB1*03:02
HLA-DQA1*04:01/DQB1*04:02
HLA-DQA1*01:01/DQB1*05:01
HLA-DQA1*01:02/DQB1*06:02
HLA-DPA1*02:01/DPB1*01:01
HLA-DPA1*01:03/DPB1*02:01
HLA-DPA1*01:03/DPB1*04:01
HLA-DPA1*03:01/DPB1*04:02
HLA-DPA1*02:01/DPB1*05:01
HLA-DPA1*02:01/DPB1*14:01

Table S15. B.Full HLA Class I reference list of the IEDB platform [(90)], which contains 27 HLA Class I A and B alleles

HLA-A*01:01
HLA-A*01:01

HLA-A*02:01
HLA-A*02:01
HLA-A*02:03
HLA-A*02:03
HLA-A*02:06
HLA-A*02:06
HLA-A*03:01
HLA-A*03:01
HLA-A*11:01
HLA-A*11:01
HLA-A*23:01
HLA-A*23:01
HLA-A*24:02
HLA-A*24:02
HLA-A*26:01
HLA-A*26:01
HLA-A*30:01
HLA-A*30:01
HLA-A*30:02
HLA-A*30:02
HLA-A*31:01
HLA-A*31:01
HLA-A*32:01
HLA-A*32:01
HLA-A*33:01
HLA-A*33:01
HLA-A*68:01
HLA-A*68:01
HLA-A*68:02
HLA-A*68:02
HLA-B*07:02
HLA-B*07:02
HLA-B*08:01

HLA-B*08:01
HLA-B*15:01
HLA-B*15:01
HLA-B*35:01
HLA-B*35:01
HLA-B*40:01
HLA-B*40:01
HLA-B*44:02
HLA-B*44:02
HLA-B*44:03
HLA-B*44:03
HLA-B*51:01
HLA-B*51:01
HLA-B*53:01
HLA-B*53:01
HLA-B*57:01
HLA-B*57:01
HLA-B*58:01
HLA-B*58:01
